# Multiscale analysis and functional validation of the cellular and genetic determinants of skeletal disease

**DOI:** 10.1038/s41588-026-02640-9

**Published:** 2026-07-10

**Authors:** Ryan C. Chai, Mischa Lundberg, Bernard Freudenthal, James T. Smith, Andrew P. Boughton, Yuandan Zhang, Kaitlyn A. Flynn, Monika Frysz, Alexander P. Corr, Weng Hua Khoo, Davide Komla-Ebri, Michael R. G. Dack, Siobhan E. Guilfoyle, John G. Logan, Natalie C. Butterfield, Victoria D. Leitch, Andrea S. Pollard, Riikka E. Mäkitie, Nathaniel Bradford, Lorenzo Ramos-Mucci, Amaia Vilas-Zornoza, Yunshun Chen, Raymond K. H. Yip, Jeremy Er, Siew Zhuan Tan, Michelle M. McDonald, Scott E. Youlten, C. Marcelo Sergio, Ariel Castro-Martinez, Shelley G. Young, Elena Skorokhodova, David M. Evans, Joseph E. Powell, Christiaan A. de Leeuw, Adam D. Ewing, John A. Eisman, Robert D. Blank, Tri Giang Phan, International Federation of Musculoskeletal Research Societies (IFMRS) Big Data Working Group, Cheryl L. Ackert-Bicknell, Douglas P. Kiel, Fernando Rivadeneira, Jennifer J. Westendorf, David Karasik, Yuuki Imai, Ralph Müller, Jason Flannick, Lynda Bonewald, Noël P. Burtt, Jonathan H. Tobias, Carolina Medina-Gomez, Qing Wu, Maria C. Costanzo, Charles R. Farber, Anne K. Lagendijk, Edwin D. Hawkins, Horng Lii Oh, Rebecca E. McIntyre, Edith M. Hessel, Jake P. Taylor-King, Paul A. Baldock, Emma L. Duncan, Graham R. Williams, J. H. Duncan Bassett, Peter I. Croucher, John P. Kemp

**Affiliations:** 1 Cancer Plasticity and Dormancy Program, Garvan Institute of Medical Research, Sydney, New South Wales Australia; 2 School of Clinical Medicine, Faculty of Medicine and Health, UNSW Sydney, Sydney, New South Wales Australia; 3 Transformational Bioinformatics, Commonwealth Scientific and Industrial Research Organisation, Sydney, New South Wales Australia; 4 UQ Frazer Institute, The University of Queensland, Woolloongabba, Queensland Australia; 5 Institute for Molecular Bioscience, The University of Queensland, St Lucia, Queensland Australia; 6 Molecular Endocrinology Laboratory, Department of Metabolism, Digestion and Reproduction, Imperial College London, London, UK; 7 Department of Biostatistics and the Center for Statistical Genetics, University of Michigan, Ann Arbor, MI USA; 8 Mater Research Institute, The University of Queensland, Translational Research Institute, Woolloongabba, Queensland Australia; 9 Musculoskeletal Research Unit, Translational Health Sciences, Southmead Hospital, University of Bristol, Bristol, UK; 10 Department of Otorhinolaryngology, Head and Neck Surgery, Helsinki University Hospital and University of Helsinki, HUS, Helsinki, Finland; 11 Faculty of Medicine, University of Helsinki, Helsinki, Finland; 12 St. Vincent’s Hospital, Darlinghurst, New South Wales Australia; 13 Relation Therapeutics, London, UK; 14 Walter and Eliza Hall Institute of Medical Research, Melbourne, Victoria Australia; 15 Department of Medical Biology, The University of Melbourne, Parkville, Victoria Australia; 16 School of Medical Sciences, Faculty of Medicine and Health, University of Sydney, Sydney, New South Wales Australia; 17 Department of Genetics, Yale School of Medicine, New Haven, CT USA; 18 MRC Integrative Epidemiology Unit, University of Bristol, Bristol, UK; 19 Translational Genomics Program, Garvan Institute of Medical Research, Sydney, New South Wales Australia; 20 UNSW Cellular Genomics Futures Institute, UNSW Sydney, Kensington, New South Wales Australia; 21 Department of Complex Trait Genetics, Center for Neurogenomics and Cognitive Research, VU University Amsterdam, Amsterdam, the Netherlands; 22 School of Medicine Sydney, University of Notre Dame Australia, Darlinghurst, New South Wales Australia; 23 Division of Endocrinology, Department of Medicine, Medical College of Wisconsin, Milwaukee, WI USA; 24 Precision Immunology Program, Garvan Institute of Medical Research, Sydney, New South Wales Australia; 25 School of Biomedical Science, The University of Queensland, St Lucia, Queensland Australia; 26 UQ Centre for Cardiovascular Health and Research (CCVHR), The University of Queensland, St Lucia, Queensland Australia; 27 School of Life Course & Population Sciences, Faculty of Life Sciences and Medicine, King’s College London, London, UK; 28 Guy’s and St Thomas’ NHS Foundation Trust, London, UK; 29 Department of Orthopedics, University of Colorado Anschutz Medical Campus, Aurora, CO USA; 30 Hinda and Arthur Marcus Institute for Aging Research, Hebrew SeniorLife Department of Medicine, Beth Israel Deaconess Medical Center and Harvard Medical School, Boston, MA USA; 31 Harvard Medical School, Boston, MA USA; 32 Broad Institute of MIT and Harvard, Cambridge, MA US; 33 Erasmus University Medical Center, Rotterdam, the Netherlands; 34 Department of Orthopedic Surgery, Mayo Clinic, Rochester, MN USA; 35 The Musculoskeletal Genetics Laboratory, The Azrieli Faculty of Medicine, Bar-Ilan University, Safed, Israel; 36 Department of Pathophysiology, Ehime University Graduate School of Medicine, Toon, Japan; 37 Division of Integrative Pathophysiology, Proteo-Science Center (PROS), Ehime University, Toon, Japan; 38 Institute for Biomechanics, Department of Health Sciences and Technology, ETH Zürich, Zürich, Switzerland; 39 Division of Genetics and Genomics at Boston Children’s Hospital, Boston, MA USA; 40 Indiana Center for Musculoskeletal Health, Indiana University, Indianapolis, IN USA; 41 Department of Biomedical Informatics, College of Medicine, The Ohio State University, Columbus, OH USA; 42 Department of Genome Sciences, University of Virginia, Charlottesville, VA USA

**Keywords:** Genome-wide association studies, RNA sequencing, Functional genomics

## Abstract

Musculoskeletal diseases are a major health burden. Development of bone-active therapies has been hindered by limited understanding of the cells and genes that regulate the skeleton. We exploited the value of cross-species analysis and developed single-cell methodologies in skeletal tissues to define the critical endosteal compartment that regulates bone turnover. Thirty-four distinct cell types were identified, and disease-relevant cells prioritized using enrichment for rare skeletal disorder genes and bone-mineral-density-associated genes in an extended UK Biobank genome-wide association study. Functional validation was undertaken in over 1,000 genetically modified mouse models. Endothelial cells and vascular smooth muscle cells were identified as new skeletal-disease-relevant cells alongside osteoblast, chondrocyte and osteoclast cell lineages. Hundreds of cell-specific genes with unappreciated roles in skeletal pathophysiology were identified. This comprehensive cellular and molecular framework underpins skeletal physiology and disease and will help prioritize new therapeutic targets to accelerate development of therapies to treat musculoskeletal disease.

## Abstract (web summary)

The study integrates cross-species single-cell and genomic analyses with functional validations to map the endosteal bone compartment, revealing musculoskeletal-disease-relevant cell types and transcriptional programs.

## Main

Musculoskeletal diseases, such as osteoporosis are a major health burden^1,2^. Existing treatments prevent skeletal deterioration but rarely restore integrity. New therapies are needed^3^; however, drug development is hindered by limited understanding of mechanisms that maintain bone mass and strength.

Gene mapping of rare and common diseases has uncovered new mechanisms of skeletal regulation^4,5^. Genome-wide association studies (GWAS) have identified over 500 loci associated with estimated bone mineral density (eBMD), a measure of skeletal integrity^6–9^. Yet at most loci, the underlying effector genes, genes with a functional role in regulating the skeleton, remain unknown. Expression quantitative trait locus (eQTL) analysis of bulk tissues can identify effector genes^10^ but is constrained by limited knowledge of gene function in specific cells^11–13^. Application of eQTL methods to single-cell transcriptomics has the potential to address this^14^. However, obtaining bone cells from healthy individuals is difficult^15^, even more so from the endosteal compartment of bone, which is a key site of bone turnover located at the interface between bone and bone marrow (BM). Consequently, bone cells are poorly represented in the Human Cell Atlas and Genotype Tissue Expression project^16,17^. Given that genes in humans and mice are highly conserved^18,19^, along with recent success in analyzing GWAS with transcriptomic data from mouse tissue^20–22^, we reasoned that isolating cells from the endosteal compartment of mice could address these challenges.

We hypothesized that integrating gene expression data from a map of cells isolated from the endosteal compartment of murine bone with human gene mapping studies of rare and common skeletal disease traits would identify the effector genes that contribute to skeletal disease. We generated a single-cell RNA sequencing (scRNA-seq) map of cells from the endosteal compartment of healthy mice. We integrated this with analyses of: (1) the nosology of rare skeletal disorders^23^; (2) a new and now largest GWAS of eBMD; (3) skeletal phenotyping data from the Mouse Genome Informatics (MGI) database; (4) an in-depth functional analysis of the skeleton in over 1,000 mouse lines with single gene deletions, and (5) validated outcomes in a scRNA-seq dataset from human bone.

Our multiscale approach defined the cellular landscape of the endosteal compartment and identified effector genes in osteoblasts, chondrocytes, osteoclasts, vascular smooth muscle cells (VSMCs) and endothelial cells (ECs) that contribute to skeletal disease. These data were made available via a bespoke web platform (www.musculoskeletal-genomics.org).

## Results

### Cells in the endosteal compartment

**Fig. 1:**
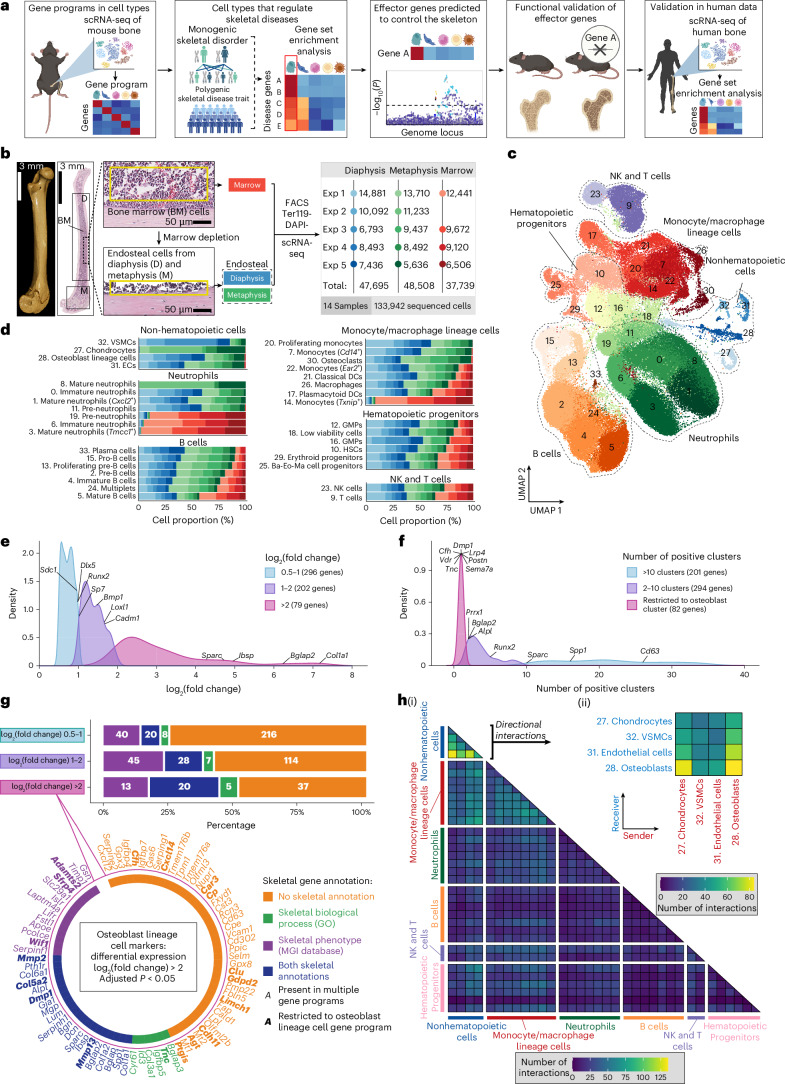
Mapping cell types in the endosteal compartment. **a**, Workflow used to identify and functionally validate effector genes at loci associated with the eBMD. Panel **a** created in BioRender; Chai, R. https://BioRender.com/4m4l20l (2026). **b**, Anatomical regions of the mouse femur from which endosteal bone cells and BM cells were isolated and sequenced for the individual experiments. The numbers in the table indicate the total number of cells present after filtering. **c**, Uniform manifold approximation and projection (UMAP) of scRNA-seq data from **b**, showing 34 clusters across six broad cell categories. **d**, Distribution of cell types in the six cell categories across the diaphysis (blue), metaphysis (green) and marrow (red). Shades denote individual experiments and are colored as in **b**. **e**,**f**, Density plots showing the log_2_(fold change) of the osteoblast lineage cluster marker genes (**e**) and the frequency of these genes across other cell clusters (**f**). **g**, Proportion of osteoblast lineage genes stratified according to log_2_(fold change) magnitude (0.5–1, 1–2 and >2). Genes annotated with skeletal process in the GO database (green), the MGI database (purple), in both databases (blue), or not annotated in either (orange) are shown. The Circos plot highlights genes with log_2_(fold change) > 2; the bold text indicates genes unique to the osteoblast lineage program. **h**, Heatmaps of CellPhoneDB-predicted intercellular interactions showing (i) all pairwise interactions across the dataset and (ii) directional interactions between nonhematopoietic clusters. GMP, granulocyte-monocyte progenitor. Source data

To identify genes that regulate the skeleton and define the cells in which they function, we developed a multiscale framework (Fig. 1a). We isolated cells from the endosteal compartments of metaphysis and diaphysis, and from the BM of femurs of adult male mice for scRNA-seq (Fig. 1b). We then defined a gene program, a set of genes upregulated in a cell cluster relative to all others, for each cell type (Supplementary Table 1).

Analysis of 133,942 cells identified 34 cell clusters (Fig. 1c) including six categories with distinct cell types: nonhematopoietic cells; monocytes and macrophages; neutrophils; hematopoietic progenitors; B cells; and natural killer (NK) and T cells (Supplementary Table 2 and Supplementary Note 1). Nonhematopoietic cells, including chondrocytes, osteoblast lineage cells, ECs and VSMCs originated largely from the endosteal compartments of the diaphysis and metaphysis (Fig. 1d). Chondrocytes were derived exclusively from the metaphysis. Cells of the monocyte and macrophage lineage, including dendritic cells (DCs) and macrophages, which may include osteal macrophages^24^, were found in both endosteal and marrow compartments. *Txnip*^+^ monocytes were found predominantly in the BM, while *Cd14*^+^ monocytes were more abundant in the endosteal compartments (Fig. 1d), an enrichment confirmed using flow cytometry (Supplementary Fig. 1). This lineage also included cells denoted as osteoclasts, which were found in both endosteal compartments. While multinucleated cells are normally excluded from scRNA-seq, these cells express osteoclast genes and may represent mononuclear osteoclasts or osteomorphs^25^. Neutrophils at different stages of maturation, hematopoietic progenitor cells (hematopoietic stem cells (HSCs), basophil-eosinophil-mast cell progenitors and granulomyeloid progenitors), B cells, T and NK cells were also identified.

Osteoblast lineage cells mediate bone formation. Therefore, we determined the gene programs that define osteoblasts and other cell types enriched in the endosteal compartment. The osteoblast gene program consisted of 577 genes (Supplementary Table 2). Seventy-nine genes (13.7%), including *Col1a1*, *Bglap2* and *Ibsp*, were highly upregulated with a log_2_(fold change) greater than 2 (Fig. 1e). While most genes (495 genes, 85.8%) were expressed in at least one other cluster, 82 genes (14.2%) had restricted expression (Supplementary Table 1 and Methods), including *Dmp1*, *Vdr* and *Postn* (Fig. 1f); 387 genes (67.1%) in the osteoblast program were neither annotated in the Gene Ontology (GO) database with skeletal terms nor with abnormal skeletal phenotypes from the MGI mammalian phenotype ontology database (Fig. 1g and Supplementary Table 3).

**Extended Data Fig. 1:**
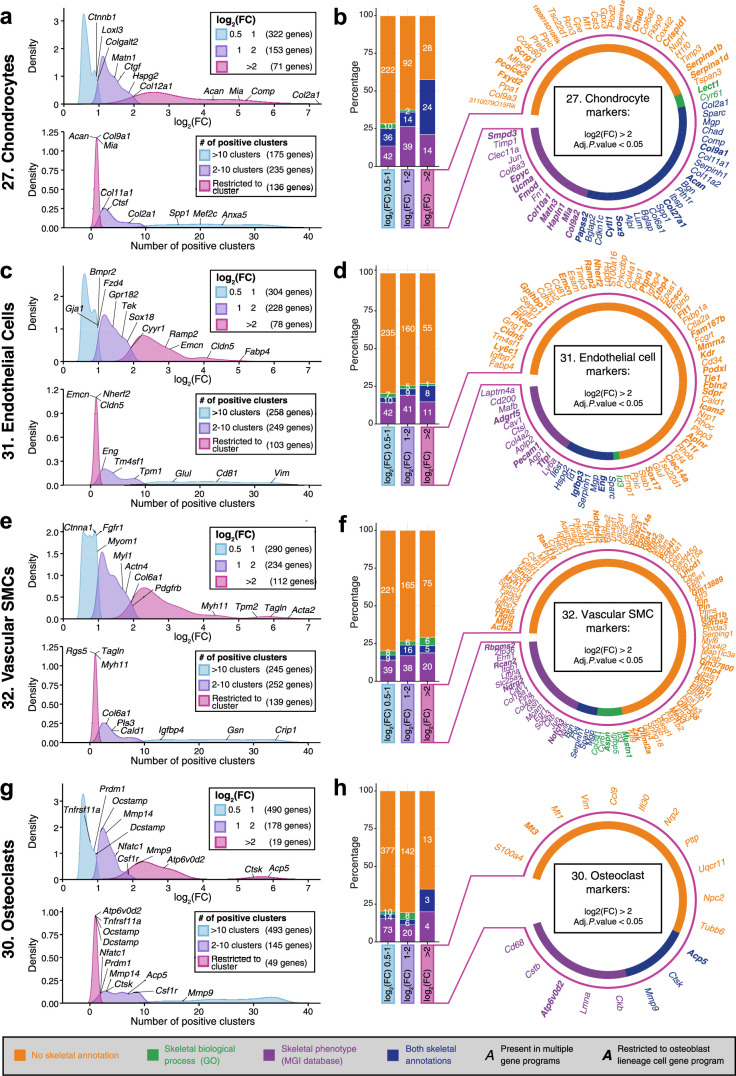
Genes and gene programs that define murine chondrocytes, endothelial cells, vascular smooth muscle cells and osteoclasts. Analysis of gene programs that define the chondrocyte (cluster 27), endothelial cell (cluster 31), VSMC (cluster 32) and osteoclast (cluster 30) clusters from Fig. 1c. (**a, c, e, g**) Density plots for each cell type (chondrocyte, endothelial cell, VSMC, osteoclast, respectively). The top plot in each panel shows the distribution of the log_2_ fold change [log_2_ (FC)] in expression of genes defining the respective gene program. The bottom plot shows the total number of other clusters expressing these genes (restrictedness). (**b, d, f, h**) Waffle plots and Circos plots for each cell type (chondrocyte, endothelial cell, VSMC, osteoclast, respectively). Waffle plots display the proportion of genes in the gene program stratified by log_2_(FC) expression bins (0.5 − 1, 1 − 2, >2). Color coding indicates skeletal annotation status: GO database (green), MGI database (purple), both (blue), or unannotated (orange). Circos plots highlight individual genes with log_2_ (FC) > 2 in expression, with those found only in that cell type’s gene program indicated in bold.

The chondrocyte program included 546 genes, with 71 highly expressed, including *Col2a1*, *Comp* and *Acan*, and 136 genes with restricted expression (Extended Data Fig. 1a,b and Supplementary Table 2). Skeletal vasculature is also important in bone development and remodeling^26^. The EC program included 610 genes, with 78 highly expressed (Extended Data Fig. 1c,d). The VSMC program included 636 genes, with 112 highly expressed (Extended Data Fig. 1e,f). Importantly, 26.9% and 27.7% of highly expressed genes in ECs and VSMCs, respectively, were annotated in the GO database with a skeletal biological process or skeletal phenotypes in the MGI database (Extended Data Fig. 1d,f). Finally, the osteoclast program included 687 genes: 19 (2.8%) were highly expressed and 49 (7.1%) expressed only in osteoclasts (Extended Data Fig. 1g,h), including *Tnfrsf11a*, *Dcstamp* and *Atp6v0d2*.

**Extended Data Fig. 2:**
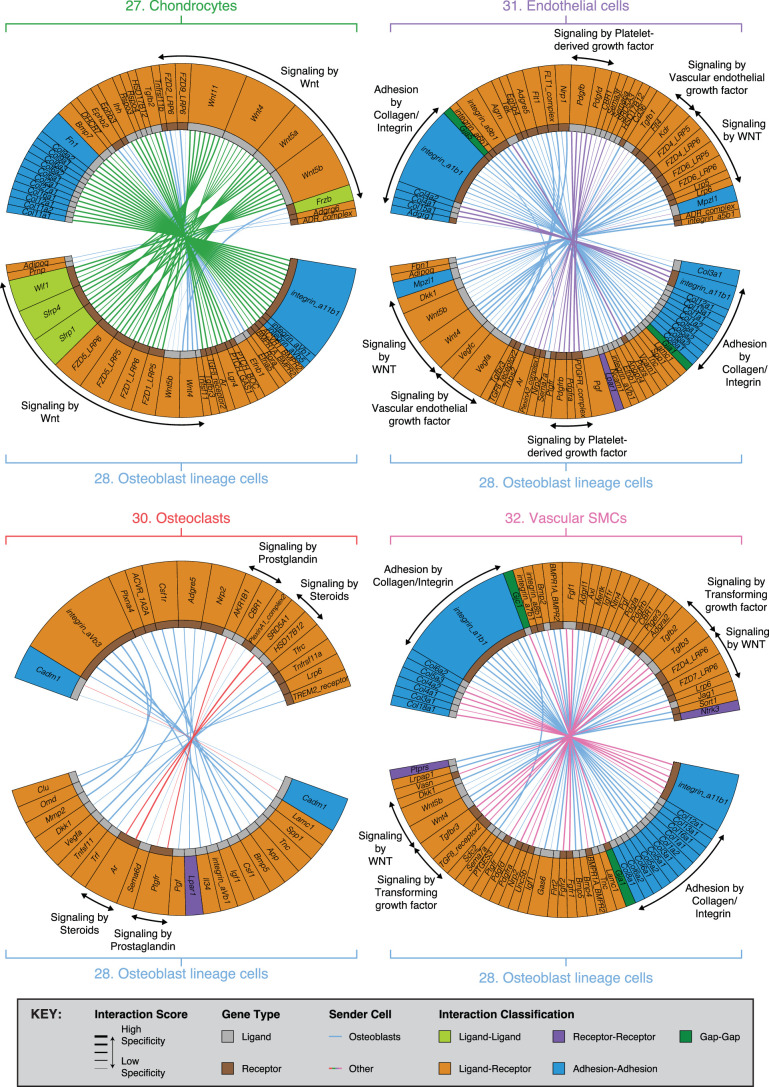
Cell-cell interaction of osteoblast lineage cells with other non-hematopoietic cells and osteoclasts. Circos plots displaying predicted interactions between osteoblast lineage cells and selected cell types. Genes are arranged according to the annotated pathways present within CellPhoneDB and labelled accordingly in the outer layer. Interaction types are indicated by coloured boxes. Line colour indicates which cell type is the “sender” cell type for a given interaction, whilst thickness reflects interaction specificity. For clarity, only interactions with a specificity score ≥50% are included within the plots.

As endosteal cells may regulate bone through direct cell–cell communication, we performed interaction analysis. This revealed the greatest interactions among nonhematopoietic cells, including between osteoblast lineage cells and the vasculature (Fig. 1h, Supplementary Table 4 and Extended Data Fig. 2). Osteoblasts interact with these cells via common (for example, transforming growth factor-β) and distinct pathways (for example, WNT with chondrocytes, NOTCH with ECs, insulin-like growth factor 1 receptor with VSMCs; Supplementary Fig. 2).

Our approach identified the cell types and gene programs that define cells in the endosteal compartment. Osteoclasts, osteoblast lineage cells, chondrocytes, ECs and VSMCs originated almost exclusively (> 98%) from this compartment. Of the 1,886 genes defining the gene programs of these cells, 1,374 (72.9%) had not previously been implicated in skeletal regulation.

### Cell states in the endosteal compartment

**Extended Data Fig. 3:**
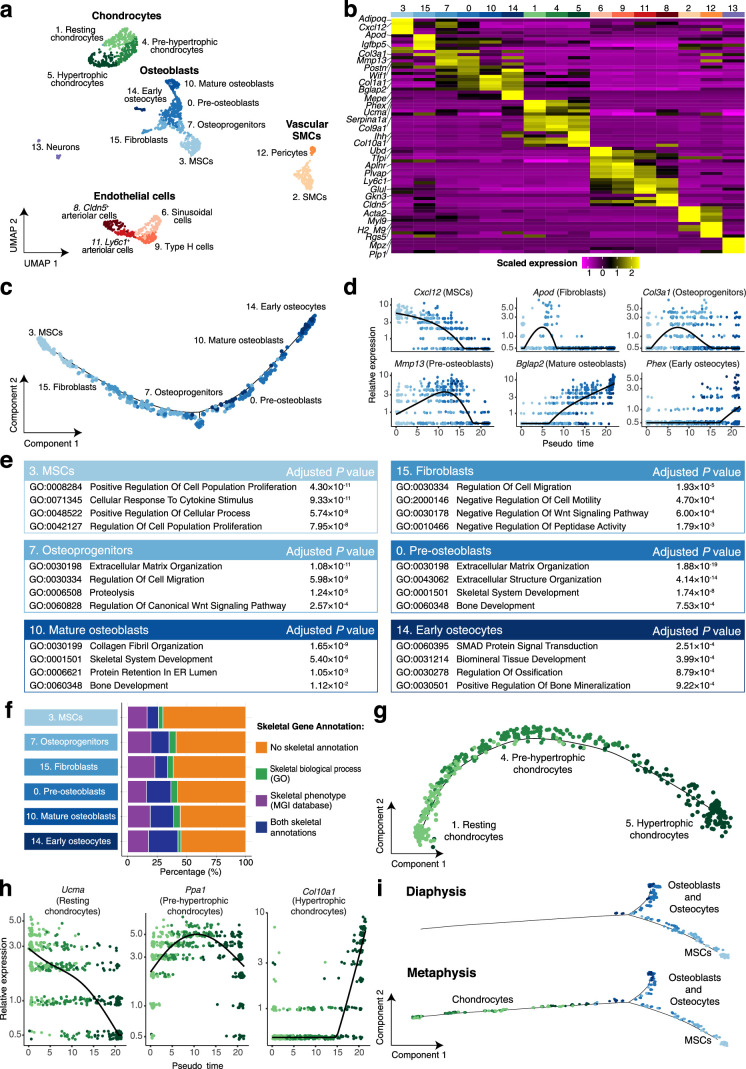
High-resolution clustering of non-hematopoietic cells. (**a**) UMAP of cell clusters identified in osteoblast lineage, chondrocyte, endothelial cell, VSMC and neuronal cell types. (**b**) Heatmap of the top 10 genes expressed by each of the non-hematopoietic sub-clusters ranked by log_2_(FC). Two example genes that define each sub-cluster are annotated. (**c**) Predicted relationship between individual osteoblast sub-clusters determined by pseudo-time trajectory analysis. (**d**) Expression of exemplar genes of each osteoblast sub-cluster in pseudo-time. (**e**) Gene ontology analysis of the gene programs for each osteoblast sub-cluster. (**f**) Bar plot showing the proportion of genes in osteoblast sub-cluster gene programs. Genes annotated with a skeletal process in the GO database (green), MGI database (purple), in both databases (blue) or are unannotated (orange) are shown. (**g**) Predicted relationship between individual chondrocyte sub-clusters determined by pseudo-time trajectory analysis. (**h**) Expression of exemplar genes of each chondrocyte sub-cluster in pseudo-time. (**i**) Predicted relationship between individual osteoblast and chondrocyte sub-clusters in diaphysis and metaphysis determined by pseudo-time trajectory analysis.

As cells are at different stages of differentiation, we used high-resolution subclustering of nonhematopoietic cells to define these stages. Sixteen clusters were identified (Extended Data Fig. 3a,b and Supplementary Table 2), including six clusters within the osteoblast lineage, three chondrocyte, four EC, two VSMC clusters and a single neuronal cluster.

**Extended Data Fig. 4:**
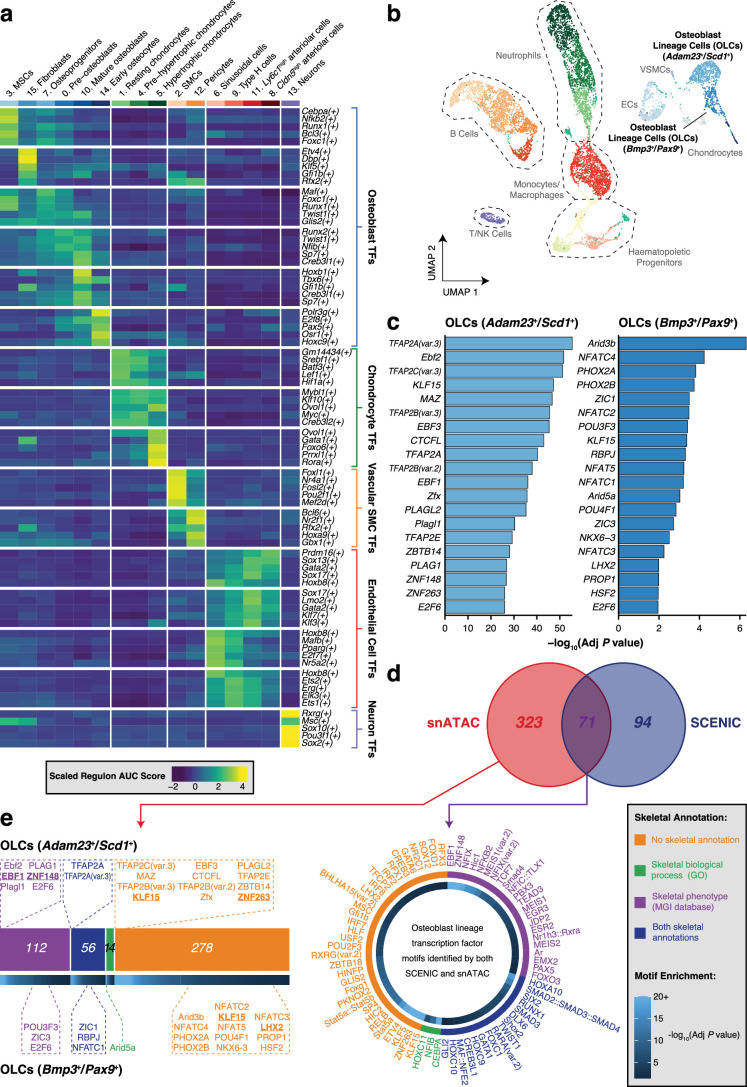
Transcription factors implicated in regulating non-hematopoietic sub-clusters. (**a**) Heatmap displaying the mean activity of the target genes (regulons) for the top 5 transcription factors predicted by SCENIC to regulate each non-hematopoietic sub-cluster. Scale bar represents AUCell scores which indicate relative expression levels of the regulon across different cell types. (**b**) UMAP of cell clusters identified by snATAC-seq. (**c**) Barplots displaying the top enriched transcription factor binding motifs within differentially accessible peaks identified within the 2 osteoblast lineage cell (OLC) clusters. (**d**) Venn diagram showing overlap of identified transcription factors from SCENIC and snATAC-seq analyses. (**e**) Barplot displaying skeletal annotations of all transcription factors identified from snATAC-seq analysis. Top transcription factors for both osteoblast lineage clusters identified from snATAC-seq analysis in (**c**) are annotated. Common transcription factors identified from both snATAC-seq and SCENIC analyses are highlighted in bold and underlined. Circos plot displaying known skeletal annotations of all common transcription factors identified from both methods. Motifs involving genes annotated with a skeletal process in the gene ontology (GO) database (green), the mouse genome informatics (MGI) database (purple), in both databases (blue) or are not annotated in either (orange) are shown. Motifs are ordered by strength of enrichment with snATAC-seq analysis, as indicated by the lower bar in barplot and inner circle of circus plot.

The osteoblast lineage formed a continuum: mesenchymal stromal cells (MSCs) progressed through fibroblasts and osteoprogenitors to pre-osteoblasts, mature osteoblasts and early osteocytes (Extended Data Fig. 3c and Supplementary Table 2). The pre-osteoblast number was higher in the diaphysis relative to metaphysis (Supplementary Fig. 3). Gene expression changed throughout differentiation (Extended Data Fig. 3b,d). *Cxcl12*^+^/*Adipoq*^+^ MSC and *Apod*^+^/*Igfbp5*^+^ fibroblast programs were enriched for GO processes in cell proliferation and migration (Extended Data Fig. 3e). *Postn*^+^/*Col3a1*^+^ osteoprogenitors and *Car3*^+^/*Mmp13*^+^ pre-osteoblasts were associated with extracellular matrix organization, *Bglap2*^+^/*Col1a1*^+^ mature osteoblasts with collagen fibril organization and *Mepe*^+^/*Phex*^+^ early osteocytes with ossification. This trajectory coincided with expression of key transcription factors (TFs) and their target genes identified using SCENIC analysis, including *Cebpa* and *Foxc1* in MSCs, which are associated with adipogenic and osteogenic programs^27,28^, as well as *Runx2* and *Sp7* in committed cells^29,30^ (Extended Data Fig. 4a and Supplementary Table 5). Single-nucleus assay for transposase-accessible chromatin using sequencing (snATAC-seq) identified unique and overlapping TF-binding motifs in osteoblast lineage cells (Extended Data Fig. 4b–e and Supplementary Table 5). snATAC-seq identified *Tfap2a*, *Ebf2* and *Zic1* as having known roles in the skeleton^31–33^, as well as new TFs such as *Ebf3*, *Maz* and *Ctcfl*. TFs identified using both methods included *Runx1* and *Creb3l1*, as well as *Klf15*, *Etv4* and *Glis2*, which have unappreciated roles in bone (Extended Data Fig. 4e).

Critically, 56.3–72.6% of genes within these osteoblast lineage gene programs were not annotated in GO or the MGI database, indicating that many genes involved in skeletal regulation have yet to be studied (Extended Data Fig. 3f).

Chondrocytes transitioned from *Ucma*^+^/*Serpina1b*^+^ resting chondrocytes to *Ppa1*^+^/*Scrg1*^+^ pre-hypertrophic chondrocytes and *Col10a1*^+^/*Ihh*^+^ hypertrophic chondrocytes, forming a distinct trajectory (Extended Data Fig. 3a,b,g–i; Supplementary Fig. 3 and Supplementary Table 2). The EC clusters included *Ubd*^+^/*Tfpi*^+^ sinusoidal cells, two clusters of arteriolar cells (*Cldn5*^+^/*Gkn3*^+^ and *Ly6c1*^+^/*Glul*^+^) and *Plvap*^+^/*Aplnr*^+^ type H cells, which have been implicated in skeletal development^34^ (Extended Data Fig. 3a,b and Supplementary Table 2). VSMC clusters included smooth muscle cells (SMCs) (*Myl9*^+^/*Acta2*^+^) and pericytes expressing *H2-M9* and *Rgs5*. Arteriolar cells (*Cldn5*^+^/*Gkn3*^+^) and SMCs were enriched in the diaphysis, whereas type H ECs were enriched in the metaphysis (Supplementary Fig. 3).

This analysis identified the stages of differentiation in non-hematopoietic cells in the endosteal compartment. Many of the genes in these cells are not known to regulate the skeleton.

### Enrichment of rare skeletal disorder genes

**Fig. 2:**
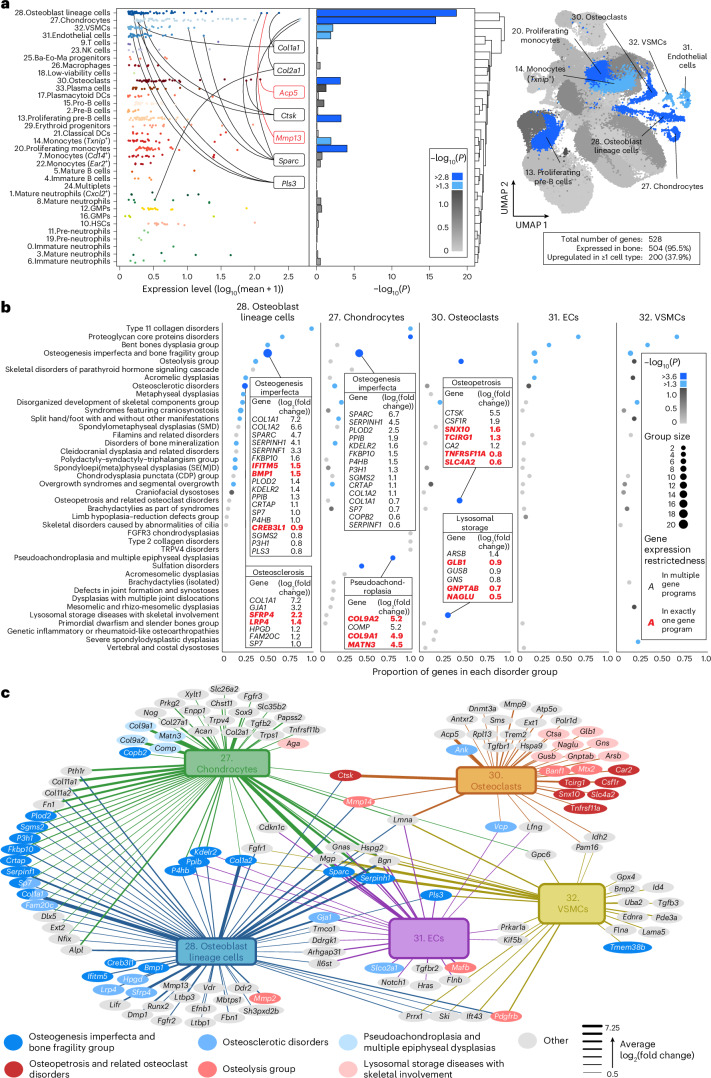
Gene programs of specific cell types are enriched for genes involved in human monogenic skeletal disorders. **a**, Dot plot showing mean expression (log_10_(mean + 1)) of known monogenic skeletal disorder genes across gene programs. Red highlights genes unique to a single program. The bar and UMAP plots indicate enrichment *P* value, as determined using a one-tailed Fisher’s exact test under the hypergeometric distribution; light blue denotes the nominal significance of *P* < 0.05 (−log_10_(*P*) of >1.3), while dark blue denotes the Bonferroni-corrected significance of *P* < 1.5 × 10^−3^ (−log_10_(*P*) of > 2.8). **b**, Bubble plot showing the number and proportion of genes from 40 monogenic skeletal disorder groups within selected cell-type gene programs. Bold indicates groups enriched in at least one cell type. Circle size represents gene count; color intensity reflects the *P* value of enrichment, as determined using a one-tailed Fisher’s exact test under the hypergeometric distribution. Ther light blue circles indicate the nominal significance of *P* < 0.05 (−log_10_(*P*) of > 1.3); dark blue circles indicate *Bonferroni*-corrected significance of *P* < 2.4 × 10^−3^ (−log_10_(*P*) of > 3.6). The boxes identify the causative genes for the indicated disorder group present in a cell-type gene program. Red denotes a gene found only in that gene program and not shared with other gene programs. **c**, Network plot illustrating the expression patterns of monogenic skeletal disorder genes across selected cell types. Nodes are color-coded according to disorder group, with line thickness representing the magnitude of differential expression (log_2_(fold change)).

We used overrepresentation analyses to identify gene programs of endosteal cells that were enriched for rare monogenic skeletal disorder genes. We used the International Skeletal Dysplasia Society nosology, a set of 528 protein-coding genes with pathogenic mutations causing 719 disorders (Supplementary Table 3)^23^. Although multiple cell types expressed causative genes, only some were highly enriched for causative genes, including osteoblast lineage cells, chondrocytes, osteoclasts, proliferating monocytes and proliferating pre-B cells (Fig. 2a and Supplementary Table 6).

**Extended Data Fig. 5:**
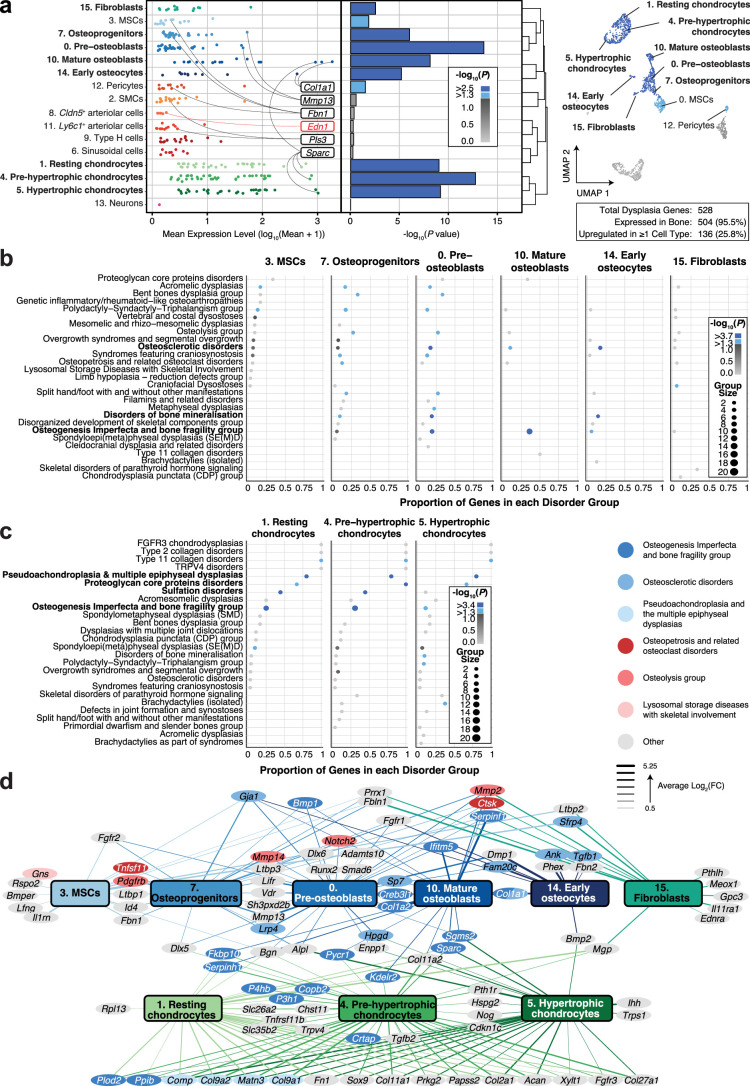
Gene programs of non-hematopoietic cells and osteoclasts are enriched for monogenic skeletal disorder genes. (**a**) Dot plot displaying the mean expression level [log_10_(mean+1)] of causative genes of rare monogenic skeletal disorders within individual non-hematopoietic sub-cluster gene programs. Boxes highlight exemplar causative genes, with red text denoting genes restricted to a single gene program. Colour shading indicates the *P* value of enrichment as determined by one-tailed Fisher’s exact test under the hypergeometric distribution: Dark blue signifies robust enrichment [*P* < 3.1×10^−3^, −log_10_(*P*) > 2.5] after *Bonferroni* correction, while light blue indicates nominal enrichment [*P* < 0.05, −log_10_(*P*) > 1.3]. (**b, c**) Bubble plots showing the number and proportion of causative genes within rare disorder groups found in gene programs for (**b**) osteoblast sub-clusters and (**c**) chondrocyte sub-clusters. Circle size represents the number of genes from a disorder group present within the gene program. The scale bar and colour shading indicate the *P* value of enrichment as determined by one-tailed Fisher’s exact test under the hypergeometric distribution. Dark blue dots denote robust *Bonferroni*-corrected enrichment [*P* < 2×10^−4^, −log_10_(*P*) > 3.7 for (**b**); *P* < 4.1×10^−4^, −log_10_(*P*) > 3.4 for (**c**)], and light blue dots denote nominal enrichment [*P* < 0.05, −log_10_(*P*) > 1.3]. (**d**) Network plot showing the unique and shared causative genes identified in the gene programs of osteoblast lineage and chondrocyte sub-clusters. Genes are colour-coded based on disorder group, and the thickness of the connecting lines indicates the magnitude of differential expression [log_2_(FC)].

Osteoblast lineage cells were enriched, with 61 genes of 553 human orthologs known to cause rare skeletal disorders (Supplementary Table 6). All differentiation states except MSCs were enriched (Extended Data Fig. 5a). Osteoblast lineage cells were primarily enriched for genes causing osteogenesis imperfecta (OI) and other bone fragility disorders, and osteosclerotic disorders (Fig. 2b,c). Eighteen of 36 known OI and bone fragility genes were present, with causative genes overrepresented in pre-osteoblasts and mature osteoblasts (Extended Data Fig. 5b,d and Supplementary Table 6). Genes causing osteosclerotic and bone mineralization disorders were enriched in pre-osteoblasts and early osteocytes.

Chondrocytes were enriched for causative genes for proteoglycan core protein disorders, sulfation disorders, pseudoachondroplasia, multiple epiphyseal dysplasias and OI and bone fragility (Fig. 2b,c). Enrichment varied among cell states (Extended Data Fig. 5c,d). Osteoclasts were enriched for genes that cause osteopetrosis and osteolysis and lysosomal storage disorders (Fig. 2b,c). Proliferating monocytes and pre-B cells were enriched for genes causing dwarfism, potentially reflecting expression of cell cycle genes^35^ (Supplementary Table 6). ECs and VSMCs showed nominal evidence of enrichment (*P* < 0.05) for proteoglycan core protein disorders and bent bone disease, possibly because of genes shared with osteoblasts (Fig. 2b,c).

### Enrichment of genes associated with eBMD

We then investigated whether gene programs of endosteal cells were enriched for eBMD-associated genes. We conducted (1) GWAS to identify genetic variants associated with eBMD; (2) gene-based tests to identify genes associated with eBMD; and (3) competitive gene set analysis (GSA) to identify gene programs enriched for eBMD-associated genes.

**Fig. 3:**
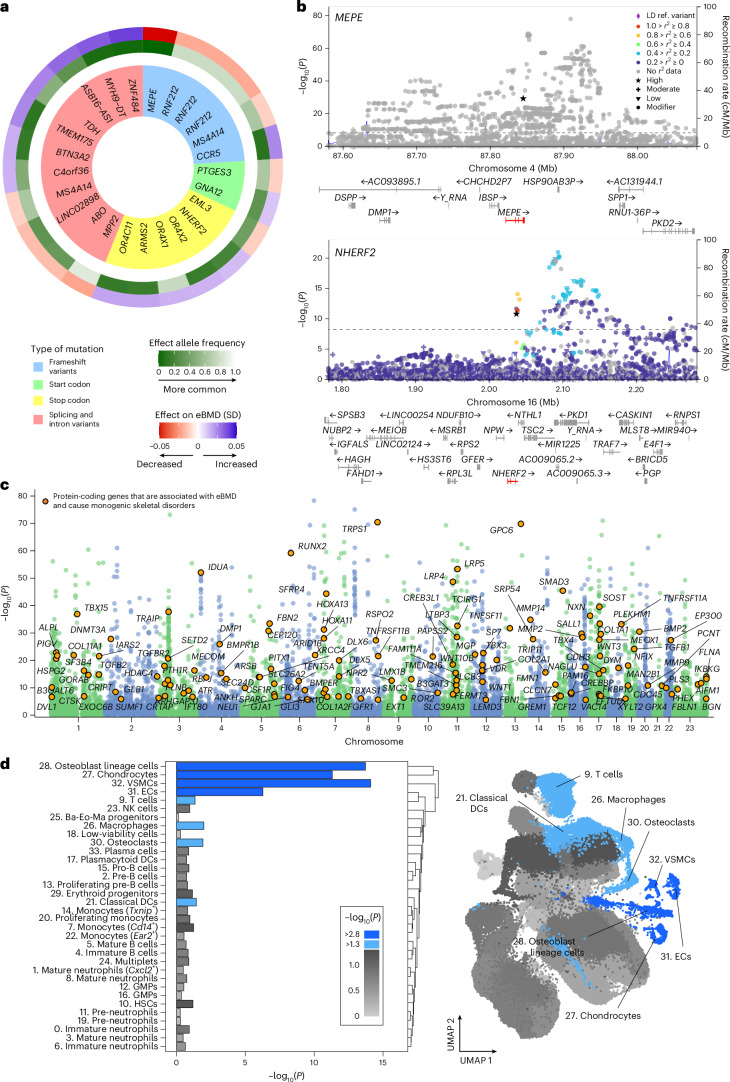
Gene programs of specific cell types are enriched for genes associated with eBMD. **a**, Circos plot of high-impact variants that are predicted to alter gene function. The inner ring denotes the type of variant, the middle ring denotes the frequency of the alternate allele and the outer ring denotes the effect of the alternate allele on eBMD. **b**, LocusZoom plots showing eBMD-associated variants mapping closest to *MEPE* and *NHERF2*. High-impact variants are represented by stars. Pairwise linkage disequilibrium (LD) could not be estimated for the lead eBMD-associated variant closest to *MEPE*. **c**, Manhattan plot summarizing the results of the genome-wide gene-based tests of eBMD conducted in the UKB study using MAGMA. The dotted line denotes the threshold of statistical significance (*P* < 2.5 × 10^−6^). Orange circles indicate genes that are associated with eBMD and are monogenic skeletal disorder genes. **d**, Bar and UMAP plots showing gene programs of different cell types that are enriched for eBMD-associated genes. The scale bar in the bar and UMAP plots indicates the *P* value, as determined using MAGMA GSA with a one-sided test for positive association. The light blue bars in the bar plot and the light blue clusters in the UMAP plot correspond to observations that have nominal evidence of enrichment: *P* < 0.05 (−log_10_(*P*) > 1.3). The dark blue bars in the bar plot and the dark blue clusters in the UMAP plot correspond to observations that have robust evidence of enrichment and meet the Bonferroni-corrected significance threshold: *P* < 1.5 × 10^−3^ (−log_10_(*P*) > 2.8).

GWAS involved 448,010 UK Biobank (UKB) participants with eBMD measures (Supplementary Table 7). This included 21,186 additional individuals, analysis of 36% more genetic variants and more detailed analysis of the X chromosome than previously performed^8^. A total of 169,684 variants were associated at genome-wide significance (*P* < 6.6 × 10^−9^)^7^. Twenty-five were high-impact variants and predicted to alter gene function (Fig. 3a and Supplementary Table 7). This included known variants in *MEPE* associated with lower eBMD^36^, and variants associated with higher eBMD, including a ‘stop/lost’ variant in the NHERF family PDZ scaffold protein 2 (*NHERF2*), a regulator of sodium transport^37^ (Fig. 3a,b and Supplementary Table 7).

**Extended Data Fig. 6:**
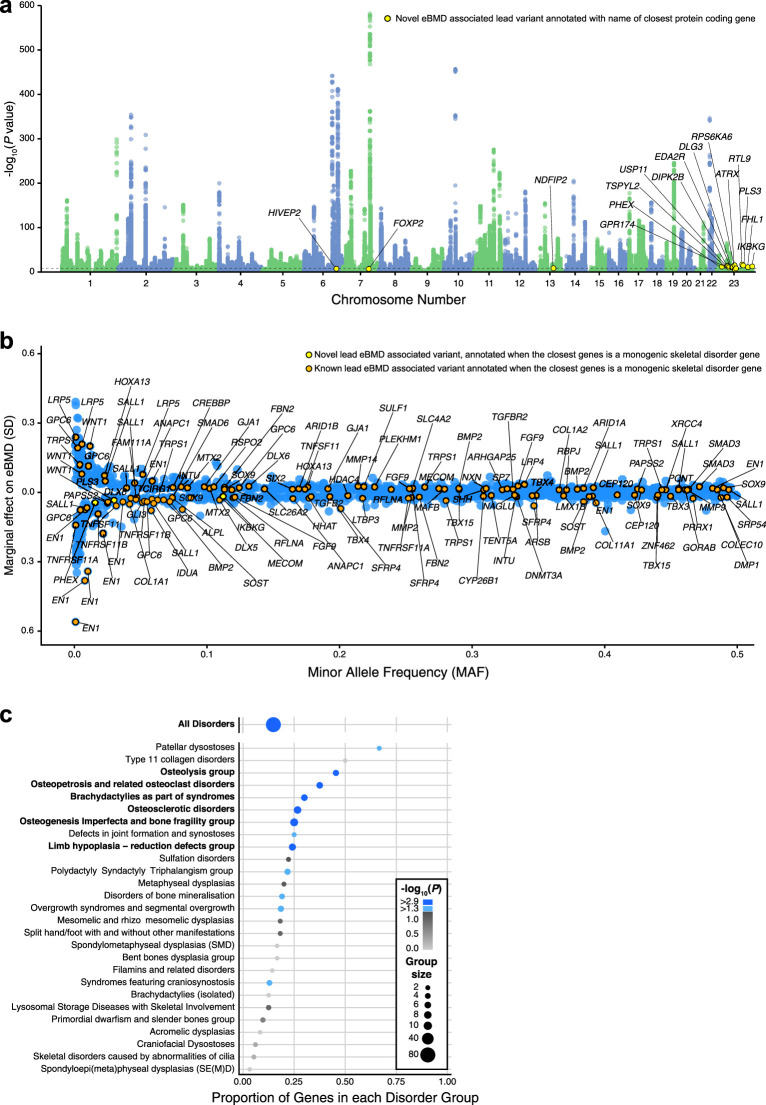
Genome-wide association study of eBMD in the UK Biobank study. (**a**) Manhattan plot summarising the results of eBMD GWAS performed on 448,010 participants in the UK Biobank study. The dashed line represents the genome-wide significance threshold (*P* < 6.6×10^−9^). Novel lead eBMD-associated variants are coloured yellow and annotated with the closest protein coding gene. The y-axis is capped at −log_10_(*P*) = 600, excluding 81 variants at the *WNT16*/*CPED1* locus on chromosome 7. Variants with *P* > 5.5×10^−5^ are also excluded. (**b**) Bivariate scatter plot contrasting the magnitude of the genetic effect on eBMD and the minor allele frequency of lead eBMD-associated variants. Orange circles correspond to lead variants mapping to known BMD-associated loci, and yellow circles correspond to lead variants mapping to novel loci. Gene symbols of monogenic skeletal disorder genes are annotated if they are located closest to a lead eBMD-associated variant. (**c**) Bubble plot showing the number and proportion of monogenic skeletal disorder genes from each disorder group present in the set of 901 protein coding genes located closest to lead eBMD-associated variants. Circle size represents the number of genes in each disorder group present. The scale bar indicates the *P* value of enrichment as determined by one-tailed Fisher’s exact test under the hypergeometric distribution. Dark blue dots denote robust enrichment [*P* < 1.2×10^−3^, −log_10_(*P*) > 2.9], and light blue dots indicate nominal evidence of enrichment [*P* < 0.05, −log_10_(*P*) > 1.3].

Fine-mapping identified 1,294 independent lead variants, defined as variants most strongly associated with eBMD that mapped closest to 903 unique protein-coding genes (Extended Data Fig. 6a and Supplementary Table 7). Sixteen lead variants represented previously unreported associations and 13 mapped to the X chromosome (Extended Data Fig. 6a). Three were closest to genes involved in monogenic disorders with abnormal BMD, including *PLS3*, which causes X-linked juvenile osteoporosis^38^, *IKBKG*, which causes osteopetrosis^39^, and *PHEX*, which causes abnormal mineralization and X-linked hypophosphatemic rickets (Extended Data Fig. 6b and Supplementary Table 7)^40^. The set of protein-coding genes closest to lead variants was enriched for genes that cause rare skeletal disorders (*P* = 2.1 × 10^−19^) (Extended Data Fig. 6c and Supplementary Table 7). These included genes involved in disorders with abnormal BMD, such as OI. Thus, our GWAS extends eBMD-associated variants from 1,103 (518 loci)^8^ to 1,294 (533 loci) and highlights variants and new genes that may regulate skeletal integrity.

As gene-based tests of association offer a powerful approach to estimate gene-trait associations, we conducted a MAGMA analysis^41^. We detected eBMD associations for 3,883 of 19,695 protein-coding genes (*P* = 2.5 × 10^−6^; Fig. 3c and Supplementary Table 7). A total of 788 eBMD-associated genes were among the 903 genes located closest to lead variants identified using GWAS (Supplementary Table 7). eBMD-associated genes were enriched for rare monogenic skeletal disorder genes (Supplementary Fig. 4a and Supplementary Table 7), including causative genes for high and low BMD disorders. Analysis of pulse rate, an unrelated trait, showed no enrichment for causative genes, suggesting that enrichment was not by chance (Supplementary Fig. 4b).

**Extended Data Fig. 7:**
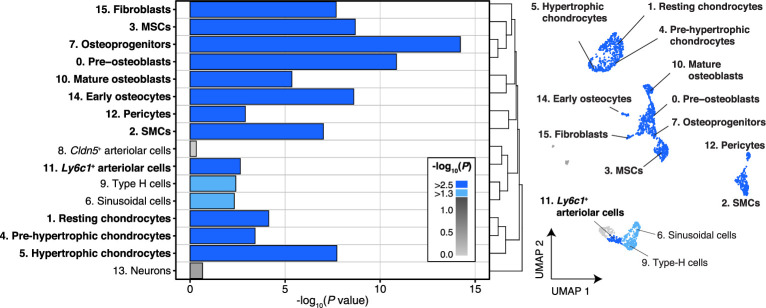
Gene programs of non-hematopoietic cell sub-clusters are enriched for eBMD-associated genes. Bar plot and UMAP plot showing enrichment of eBMD-associated genes in gene programs for different cell types identified in scRNA-seq as determined by MAGMA competitive gene set analyses using a one-sided test for positive association. Scale bar in bar plot and UMAP plot indicates the *P* value. Light blue bars in bar plot and light blue dots in UMAP correspond to observations that have nominal evidence of enrichment: *P* < 0.05 [−log_10_(*P*) of >1.3]. Dark blue bars in bar plot and dark blue dots in UMAP correspond to observations that have robust evidence of enrichment and meet the *Bonferroni*-corrected significance threshold: *P* < 3 ×10^−3^ [−log_10_(*P*) > 2.5].

GSA showed the gene programs of osteoblast lineage cells, chondrocytes, ECs and VSMCs, were enriched for eBMD-associated genes (Fig. 3d and Supplementary Table 7). All stages of osteoblast, chondrocyte, VSMC differentiation, and one of four EC states were enriched for eBMD-associated genes (Extended Data Fig. 7 and Supplementary Table 7). As 57% of genes in nonhematopoietic cell gene programs were present in at least one other program, we performed pairwise conditional GSA to determine whether enrichment was confounded by shared genes (Supplementary Fig. 5a,b and Supplementary Table 7). Osteoblast lineage cells, chondrocytes, ECs and VSMCs remained enriched for genes associated with eBMD (*P*_conditional_ < 0.05; Supplementary Fig. 5b and Supplementary Table 7). Given that a subset of genes could influence enrichment, we also performed a post-hoc permutation analysis; 93–99% of genes contributed to enrichment, suggesting that small numbers of genes were not unduly influential (Supplementary Fig. 5c).

Together, this demonstrates that gene programs of osteoblast lineage cells, chondrocytes, ECs and VSMCs are enriched for genes associated with eBMD.

### Enrichment of eBMD effector genes known to regulate bone

**Extended Data Fig. 8:**
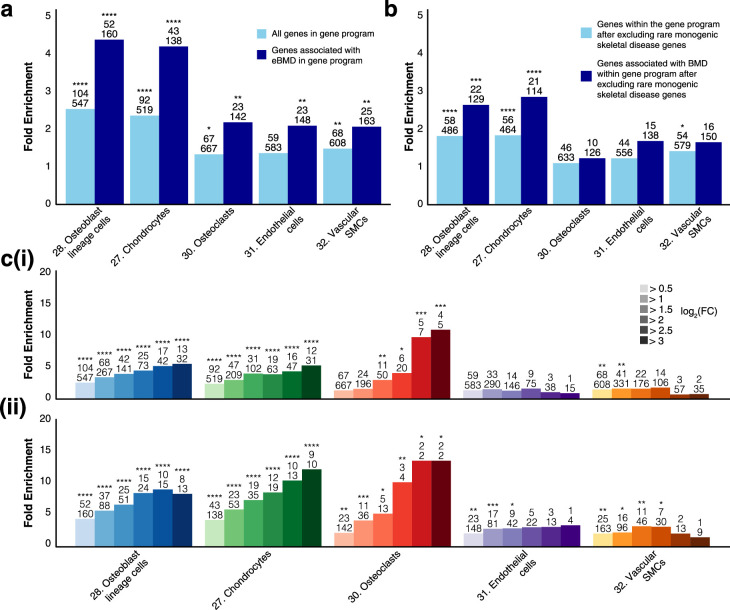
Gene programs of non-hematopoietic cells and osteoclasts are enriched for genes that cause abnormal bone structure when mutated in mice. This figure details the enrichment of cell type gene programs for genes associated with abnormal bone structure in the MGI database. (**a**) Enrichment of gene programs for MGI abnormal bone structure genes. Light blue bars represent all genes in the gene program; dark blue bars represent the subset of these genes also associated with eBMD. Numbers indicate gene counts (top: MGI genes in gene program; bottom: total genes in gene program). Enrichment was determined by hypergeometric over-representation testing. *Bonferroni*-corrected significance thresholds are indicated. (**b**) Enrichment analysis excluding genes that cause monogenic skeletal disorders. Light blue bars represent non-causative genes; dark blue bars represent non-causative genes associated with eBMD. Numbers show gene counts. (**c**) Fold-change stratified enrichment for genes causing abnormal BMD in mice. Genes are allocated to six nested groups based on magnitude of differential expression [log_2_(FC), increasing left to right]. Panel (i) includes all genes in gene program, while panel (ii) includes only eBMD-associated genes. *Bonferroni*-corrected significance is indicated. **** *P* < 0.001, *** *P* < 0.005, ** *P* < 0.01, * *P* < 0.05. All statistics are determined by one-tailed Fisher’s exact tests under the hypergeometric distribution.

We next investigated whether eBMD-associated genes in nonhematopoietic cell and osteoclast programs influence skeletal structure. We identified 1,347 protein-coding genes in the MGI mouse phenotype ontology database with human orthologs that when mutated cause ‘abnormal bone structure’ (Supplementary Table 3). Osteoblasts, chondrocytes, osteoclasts and VSMCs, but not ECs, were highly enriched (Extended Data Fig. 8a and Supplementary Table 8). Enrichment increased when the analysis was restricted to eBMD-associated genes (Extended Data Fig. 8a, Supplementary Fig. 6a and Supplementary Table 8). Sensitivity analyses excluding monogenic disorder genes indicated that enrichment persisted for osteoblasts, chondrocytes and VSMCs (Extended Data Fig. 8b and Supplementary Table 8). Enrichment increased for genes with the greatest expression in osteoblast, chondrocyte and osteoclast gene programs and increased further when restricted to genes associated with eBMD (Extended Data Fig. 8c, Supplementary Fig. 6b and Supplementary Table 8).

### New eBMD effector genes regulate structure and function

**Fig. 4:**
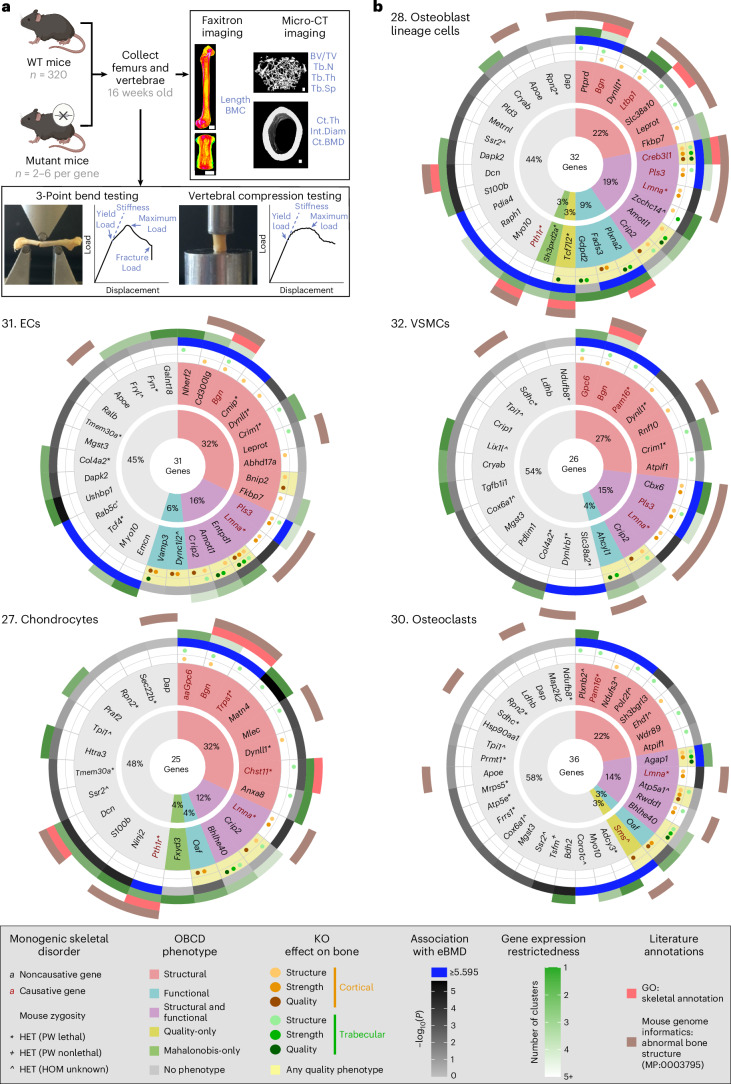
Genes that cause abnormal skeletal phenotypes when deleted in mice. **a**, The OBCD phenotyping pipeline. Panel **a** created in BioRender; Chai, R. https://BioRender.com/4m4l20l (2026). **b**, Skeletal effects of deleting genes identified in nonhematopoietic cell and osteoclast gene programs. Circos plots represent individual cell clusters identified in Fig. 1, with total gene counts indicated at the center of each plot. From inner to outer rings: (1) percentage of KO mice with skeletal phenotypes; (2) individual deleted genes (red indicates monogenic disorder gene orthologs; symbols denote homozygous lethality for heterozygous models); (3) effects on cortical bone; (4) effects on trabecular bone; (5) MAGMA-gene-based eBMD association (blue/gray); (6) cell-type-restricted expression (green); (7) GO skeletal process annotation; and (8) MGI abnormal bone structure annotation. The key summarizes the color coding for each phenotype, association with monogenic disorders and eBMD, gene expression restrictedness and skeleton-related annotations. **a**, Scale bar in Faxitron images of femur and vertebra, 1 mm; scale bars in micro-CT images, 100 μm. Ct.BMD, cortical BMD; Int.Diam, internal diameter. Source data

We next determined whether eBMD-associated genes expressed in nonhematopoietic cells and osteoclasts have functional roles in bone and are effector genes. We screened adult female mice from more than 1,000 mouse lines with gene deletions that have undergone structural and functional skeletal phenotyping in our Origin of Bone and Cartilage Disease (OBCD) study (Fig. 4a; see Methods and Supplementary Table 1 for definitions). This extends the original 100 lines^42^ and the 596 lines reported in 2019 (ref. ^8^).

Of the 1,886 genes in the five programs, 101 (5.3%) had deletions in the OBCD study (Fig. 4b); 32 in the osteoblast program; 25 in chondrocytes; 31 in ECs; 26 in VSMCs; and 36 in osteoclasts. Fifty-one (50.5%) exhibited abnormal structural, functional or bone quality phenotypes (Fig. 4b and Supplementary Table 9).

Some genes had established skeletal roles, including *Bgn* (biglycan)^43^, *Creb3l1* (cAMP responsive element binding protein 3 like 1)^44^, *Gpc6* (glypican 6)^7^ and *Pls3* (plastin 3)^38^. Many genes identified were not annotated in the GO database or had no or limited skeletal impact in the MGI database, suggesting they are unknown regulators of bone. These included cell-type-restricted genes like *Ptprd*, *Nherf2* (ECs), *Cbx6* (VSMCs) and *Agap1* (osteoclasts) (Fig. 4b and Supplementary Table 9). This suggests that the gene programs defining cells in the endosteal compartment include new eBMD effector genes.

#### PLS3

**Fig. 5:**
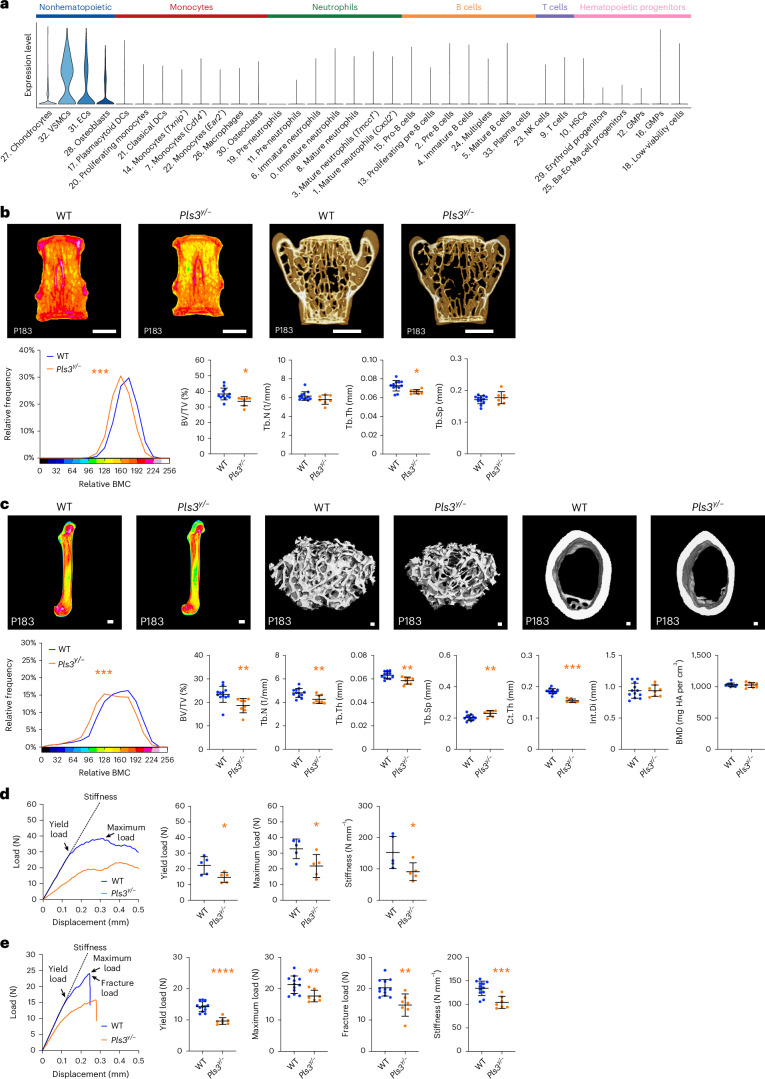
Skeletal phenotype of male *Pls3*^*y*/^^−^mice. **a**, Violin plots of *Pls3* expression in the endosteal bone and BM scRNA-seq dataset. **b**, Lumbar vertebra (L5) analysis from P183 mice (WT *n* = 12; *Pls3*^*y*/−^ *n* = 8). Left: Pseudocolored X-ray microradiography and BMC relative frequency histograms (Kolmogorov–Smirnov test, *P* < 0.001). Low BMC is shown in blue/green; high BMC is shown in pink. Right: Mid-coronal micro-CT sections and quantification of bone volume as a proportion of tissue volume (BV/TV), Tb.N, Tb.Th and Tb.Sp. **c**, Femur analysis from P183 mice (WT *n* = 12; *Pls3*^*y*/−^ *n* = 8). Left: Pseudocolored X-ray microradiography and BMC histograms **(***P* < 0.001). Low BMC is shown in blue/green; high BMC is shown in pink. Middle: Distal femur micro-CT and trabecular parameters. Right: Mid-shaft cortical micro-CT and Ct.Th, Int.Di and Ct.BMD. **d**, Compression testing of L5 vertebrae (WT *n* = 5, *Pls3*^*y*/−^ n = 5). Representative load displacement curves and quantification of yield load, maximum load and stiffness. **e**, Three-point bend testing of femurs (WT *n* = 12; *Pls3*^*y*/−^ *n* = 7). Representative load displacement curves and quantification of yield load, maximum load, fracture load and stiffness. All dot plots represent the mean ± s.d.; two-tailed Student’s *t*-test; *P < 0.05, **P < 0.01, ***P < 0.001. **b**, Scale bar, 1 mm. **c**, Scale bars, 1 mm and 100 μm (mid-shaft cortical micro-CT). Source data

To explore a widely expressed effector gene, we performed studies on *Pls3*. While *PLS3* loss-of-function mutations cause OI and X-linked osteoporosis in children^38^, we also found that the common genetic variation is associated with eBMD in adults (Fig. 3c, Extended Data Fig. 6a and Supplementary Table 7). *Pls3* was expressed in osteoblast lineage cells, ECs and VSMCs in bone (Fig. 5a), and in epithelial, mesenchymal and vascular cells outside bone (Supplementary Fig. 7 and Supplementary Table 10). It is unclear which cells it acts through to affect bone integrity^45–48^.

As X-linked osteoporosis is characterized by vertebral fractures in young males, we characterized lumbar vertebrae from *Pls3*-deficient male mice (*Pls3*^*y*/−^). Bone mineral content (BMC) was reduced in vertebrae in *Pls3*^*y*/−^ mice and micro-computed tomography (CT) analysis demonstrated decreased bone volume (BV/TV) and trabecular thickness (Tb.Th) (Fig. 5b and Supplementary Fig. 8a). In long bones, *Pls3*^*y*/−^ mice also showed decreased BMC, reduced BV/TV, trabecular number (Tb.N) and Tb.Th, and increased trabecular spacing (Tb.Sp) (Fig. 5c and Supplementary Fig. 8c,d). Cortical thickness (Ct.Th) was reduced (Fig. 5c and Supplementary Fig. 8e). Biomechanical testing demonstrated that maximum load was impaired in vertebrae at P70; yield load, maximum load and stiffness were all reduced (Fig. 5d and Supplementary Fig. 8b). Long bones also exhibited a reduced yield load, maximum load and stiffness in *Pls3*^*y*/−^ mice (Fig. 5e and Supplementary Fig. 8f). Female *Pls3*-deficient (*Pls3*^−/−^) mice demonstrated a similar phenotype (Supplementary Fig. 9).

**Extended Data Fig. 9:**
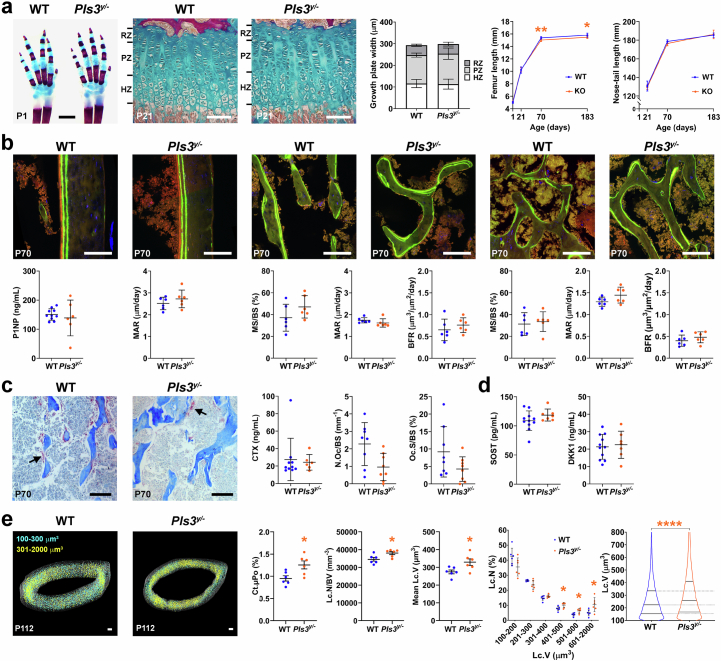
Cellular mechanism of skeletal phenotype in *Pls3* deficient mice. (**a**) Left panel: Alcian blue and Alizarin red-stained upper limb paws from P1 mice; scale bar = 1 mm. Second panel: P21 proximal tibia growth plate sections stained with Alcian blue and van Gieson; scale bars = 100μm. Bar plot shows widths of the growth plate zones (resting zone, RZ; proliferating zone, PZ; hypertrophic zone, HZ). Line plots show femur and nose-to-tail length measured across various ages. Mean±s.d; two-tailed Student’s *t*-test; *P < 0.05; **P < 0.01. (**b**) Confocal images of P70 mid-femur cortical, distal femur trabecular, and lumbar vertebral trabecular bone (L1-L3) following calcein double-labelling; scale bars=100μm. Graphs show serum P1NP levels (P70) and dynamic histomorphometry parameters: MAR (mineral apposition rate), MS/BS (mineralising surface per bone surface), and BFR (bone formation rate) for both femur and lumbar vertebrae (P70). Mean±s.d. (**c**) Decalcified proximal tibia sections from P70 mice stained for TRAP and aniline blue. Black arrows indicate osteoclasts. Scale bars = 100μm. Graphs show serum CTX levels (P70) and osteoclast parameters: osteoclast number (OcN/BS) and osteoclast surface (OcS/BS) (P70). Mean±s.d. (**d**) Graphs show serum levels (mean±s.d) of Wnt inhibitors sclerostin (SOST) and dickkopf-related protein 1 (DKK1) in P183 mice. (**e**) μCT images (P112) of a 250μm mid-femur ROI, showing small (blue) and large (yellow) osteocyte lacunae. Scale bars = 100μm. Graphs quantify cortical microporosity (Ct.μPo), lacuna number (Lc.N/BV), mean lacuna volume (Mean Lc.V), and lacuna number by volume (mean± s.d; two-tailed Student’s *t*-test; *P < 0.05). Violin plot shows the distribution of Lc.V (Kolmogorov-Smirnov test; ****P < 0.0001). Source data

To investigate the cellular mechanism, we analyzed the major bone cell lineages. Analysis of the postnatal day 1 (P1) skeleton revealed no differences in endochondral ossification (Extended Data Fig. 9a). There were no differences in growth plate reserve and proliferative and hypertrophic zones at P21; linear growth did not differ (Extended Data Fig. 9a). Static and dynamic histomorphometry and serum measures of osteoclastic resorption, osteoblastic bone formation and osteocyte-derived proteins were unaffected in *Pls3*^y/−^ mice (Extended Data Fig. 9b–d). Cortical porosity and osteocyte lacuna size and number were increased in *Pls3*^*y*/−^ mice (Extended Data Fig. 9e).

**Fig. 6:**
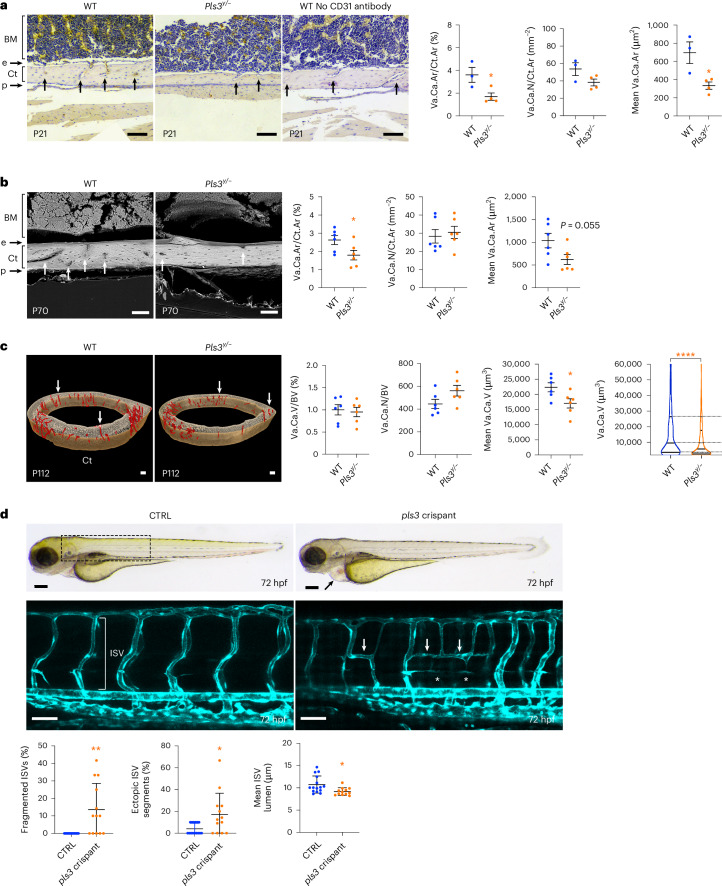
Vascular phenotype of male *Pls3*^*y*^^/^^−^ mice and *pls3* crispant zebrafish. **a**, Anti-CD31 immunohistochemistry of P21 mid-femur sections (WT *n* = 3; *Pls3*^*y*/−^ *n* = 4). The arrows indicate the cortical vascular canals. Graphs quantify the vascular canal area as a proportion of the cortical area (Va.Ca.Ar/Ct.Ar), vascular canal number per cortical area (Va.Ca.N/Ct.Ar) and mean vascular canal area (Va.Ca.Ar). **b**, Backscattered SEM images of triiodide-stained P70 mid-femur sections (WT *n* = 6, *Pls3*^*y*/−^ *n* = 6). The arrows indicate the vascular canals. Graphs quantify Va.Ca.Ar/Ct.Ar, Va.Ca.N/Ct.Ar and mean Va.Ca.Ar. **c**, Micro-CT of P112 mid-femur region of interest (ROI) (250 μm) showing cortical vessels in red (WT *n* = 6, *Pls3*^*y*/−^ *n* = 6). Graphs quantify vascular canal volume as a proportion of bone volume (Va.Ca.V/BV), vascular canal number per bone volume (Va.Ca.N/BV) and mean vascular canal volume (Va.Ca.Ar). The violin plot shows the Va.Ca.V distribution (Kolmogorov–Smirnov test, ****P < 0.0001). **d**, Top: Morphology of CTRL (*n* = 15) and *pls3* crispant (*n* = 13) zebrafish at 72 h post fertilization (hpf). The arrow indicates pericardial edema; the box denotes the ROI. Bottom: Representative maximum intensity projections of *Tg(kdr-l:eGFP)* expression in the trunk vasculature. The asterisks indicate incomplete ISVs, disconnected from the dorsal aorta or posterior cardinal vein; the white arrows show ectopic ISV segments connecting neighboring ISVs in *pls3* crispants. Graphs quantify the percentage of fragmented ISVs, the percentage of ectopic segments and the mean ISV luminal diameter. Unless otherwise stated, all dot plots represent the mean ± s.d; two-tailed Student’s *t*-test; *P < 0.05, **P < 0.01. **a**, Scale bar, 100 μm. **b**,**c**, Scale bar, 100 μm. **d**, Scale bar, 50 μm. ct, cortical bone; e, endosteum; p, periosteum. Source data

As *Pls3* was most highly expressed in VSMCs and ECs (Fig. 5a), we examined the vasculature in bone. *Pls3*^*y*/−^ mice showed smaller blood vessels in bone compared to controls (CTRLs) (Fig. 6a), which was confirmed by backscattered scanning electron microscopy (SEM) and micro-CT analysis (Fig. 6b,c and Supplementary Fig. 10). Knockdown of *pls3* in zebrafish embryos^49^ resulted in mild cardiac edema and defects in intersegmental vessel (ISV) formation, including disconnections from the dorsal aorta or posterior cardinal vein, ectopic vessel connections and smaller lumens (Fig. 6d). The absence of bone cells at this developmental stage indicates that *pls3* might act directly to control angiogenesis and vascular patterning. These data suggest that X-linked osteoporosis might result from abnormalities in bone vasculature.

#### Restricted eBMD genes

**Fig. 7:**
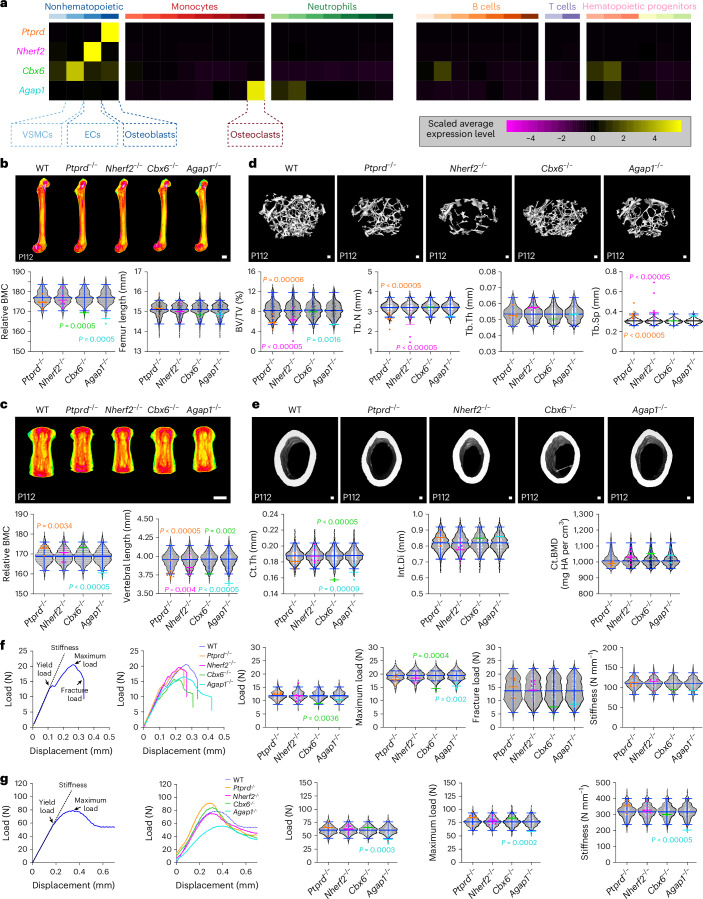
Skeletal phenotypes of female *Ptprd*^−/−^, *Cbx6*^−/−^, *Nherf2*^−/−^ and *Agap1*^−/−^ mice. **a**, Heatmaps of *Ptprd*, *Nherf2*, *Cbx6* and *Agap1* expression in the endosteal bone and BM scRNA-seq dataset. **b**,**c**, Pseudocolored X-ray microradiography of femurs (**b**) and caudal vertebrae (**c**) from P112 WT and KO mice. Graphs show relative BMC (mean ± s.d.) and length (femur: median ± 2.5th and 97.5th percentiles; vertebrae: mean ± s.d.). WT reference ranges (*n* = 320) are gray violins with individual white dots. Individual values and mean or median for *Ptprd*^−/−^ (orange, *n* = 6), *Nherf2*^−/−^ (pink, *n* = 6), *Cbx6*^−/−^ (green, *n* = 2) and *Agap1*^−/−^ (cyan, *n* = 2) are shown. **d**,**e**, Micro-CT images and quantification of distal femur trabecular bone (**d**) (BV/TV, Tb.N, Tb.Th: mean ± s.d.; Tb.Sp: median ± 2.5th and 97.5th percentiles) and mid-shaft cortical bone (**e**) (Ct.Th, Int.Di: mean ± s.d.; Ct.BMD: median ± 2.5th and 97.5th percentiles). **f**,**g**, Example (left) and representative (right) load displacement curves from femur three-point bending (**f**) and caudal vertebrae compression (**g**) for WT (dotted blue), *Ptprd*^−/−^ (orange), *Nherf2*^−/−^ (pink), *Cbx6*^−/−^ (green) and *Agap1*^−/−^ (cyan). Graphs show yield load (mean ± s.d), maximum load (mean ± s.d), fracture load (median ± 2.5th and 97.5th percentiles) and stiffness (mean ± s.d). For all graphs, significant *P* values after permutation testing as detailed in the Methods are indicated. **b**,**c**, Scale bar, 1 mm. **d**,**e**, Scale bar, 100 μm. Source data

To understand eBMD-associated genes with restricted expression, we investigated the impact of deleting *Ptprd*, *Nherf2*, *Cbx6* and *Agap1* on bone structure and function. *Ptprd* was restricted to osteoblast lineage cells in bone (Fig. 7a) and expressed in mesenchymal, epithelial and glial cells in lung, brain and ovary (Supplementary Fig. 7 and Supplementary Table 10). Female *Ptprd*^−/−^ mice had normal femoral BMC and length but increased vertebral BMC and decreased vertebral length compared to the wild-type (WT) mice reference range of 320 female mice from an identical genetic background (Fig. 7b,c). Femurs from *Ptprd*^−/−^ mice showed decreased BV/TV and Tb.N with increased Tb.Sp but normal cortical bone (Fig. 7d,e). Femoral and vertebral bone strength were unchanged (Fig. 7f,g). *Nherf2* expression was restricted to ECs in the endosteal compartment (Fig. 7a) and expressed in ECs in other tissues (Supplementary Fig. 7 and Supplementary Table 10), suggesting that *Nherf2* is important in endothelial function. *Nherf2*^−/−^ mice had decreased vertebral length (Fig. 7c) and reduced femoral BV/TV and Tb.N with increased Tb.Sp (Fig. 7d). Bone strength and stiffness parameters were unchanged (Fig. 7f,g). *Cbx6* expression was restricted to VSMCs in the skeleton (Fig. 7a) but also expressed in the lung, brain, pancreas and stomach (Supplementary Fig. 7 and Supplementary Table 10). *Cbx6*^−/−^ mice exhibited decreased femoral BMC and vertebral length (Fig. 7b,c) and reduced femoral cortical thickness (Ct.Th) (Fig. 7e). Femur yield load and maximum load were decreased compared to WT mice (Fig. 7f,g). Finally, *Agap1* expression was restricted to osteoclasts in bone but also expressed in the heart, brain and liver (Supplementary Fig. 7 and Supplementary Table 10). *Agap1*^−/−^ mice had decreased femur BMC and decreased vertebral BMC and length (Fig. 7b,c). Femurs also had decreased BV/TV and Ct.Th (Fig. 7d,e). Femur maximum load and vertebral yield load, maximum load and stiffness were all decreased (Fig. 7f,g).

### Cells and genes involved in skeletal diseases in human bone

**Fig. 8:**
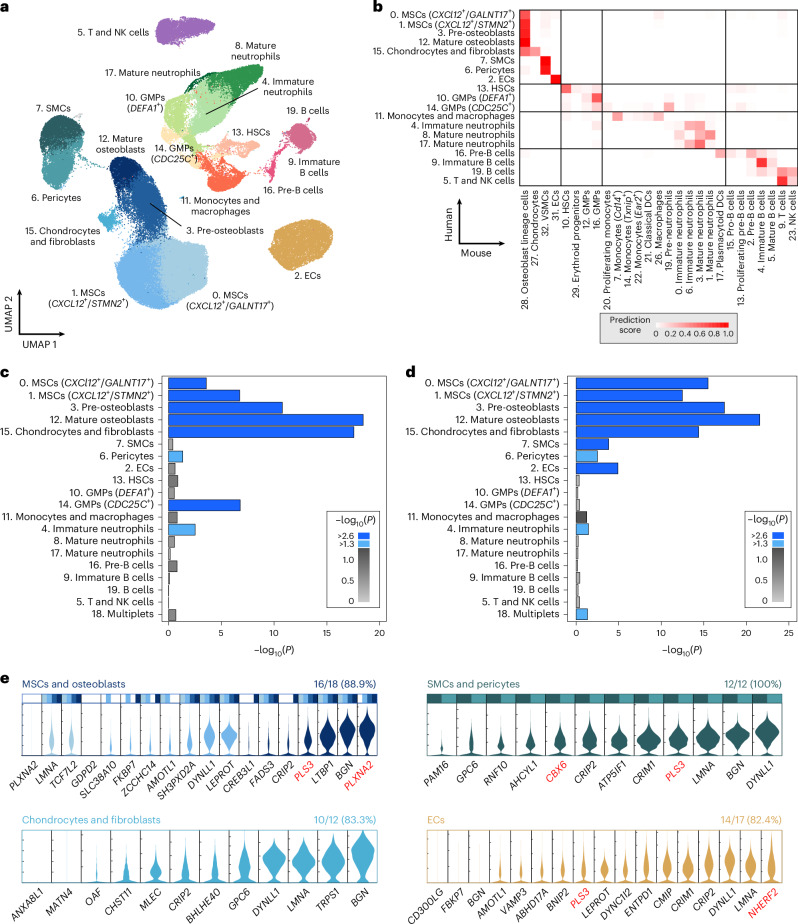
Mapping the cell types and genes involved in skeletal diseases in human bone. **a**, UMAP plot derived from scRNA-seq data of cells isolated from human femoral head. **b**, Label transfer analysis using mouse scRNA-seq data from Fig. 1 as a reference. Prediction scores reflect the probability assigned to a transferred label for a given query cell cluster. **c**,**d**, Bar plots showing enrichment of gene programs for causative genes of monogenic skeletal disorders (**c**) and eBMD-associated genes (**d**). The scale bars in the bar plots indicate the *P* value. The light blue bars correspond to observations that have nominal evidence of enrichment: *P* value < 0.05 (−log_10_(*P*) > 1.3). The dark blue bars correspond to observations that meet the Bonferroni-corrected significance threshold: *P* < 2.5 × 10^−3^ (−log_10_(*P*) > 2.6) as determined using a one-tailed Fisher’s exact test under the hypergeometric distribution (**c**) or MAGMA GSA using a one-sided test for positive association (**d**). **e**, Violin plots showing the expression of exemplar genes (cell-type-specific genes that are eBMD-associated and functionally validated in mice) in human bone. Where multiple clusters are present for a lineage, a single violin is shown corresponding to the cluster with the highest expression, with the bars above indicating which clusters pass the threshold for active expression of the gene (Methods), both colored according to the UMAP in **a**. The numbers above the plots for each lineage indicate the proportion of genes that pass the threshold for active expression. Selected exemplars from Figs. 5 and 7 are highlighted in red.

To investigate the translational importance of our findings, we isolated and sequenced 125,063 cells from adult human femoral bone (Fig. 8a). Human bone included similar populations of MSCs, pre-osteoblasts, mature osteoblasts, chondrocytes, fibroblasts, ECs and VSMCs to mice (Fig. 8a,b and Supplementary Table 2).

The gene programs that define human MSCs, pre-osteoblasts, mature osteoblasts, chondrocytes and fibroblasts were enriched for genes that cause monogenic skeletal disorders and associated with eBMD (Fig. 8c,d and Supplementary Tables 6 and 7). Human VSMCs and ECs were also enriched for eBMD-associated genes (Fig. 8d); 82–100% of eBMD effector genes that we validated in mice were also found in their cellular counterparts in human bone (Fig. 8e). This included *PLS3* in MSCs, osteoblasts, SMCs, pericytes and ECs; *PTPRD* in MSCs and osteoblasts; *CBX6* in SMCs and pericytes; and *NHERF2* in ECs (Fig. 8e).

**Extended Data Fig. 10:**
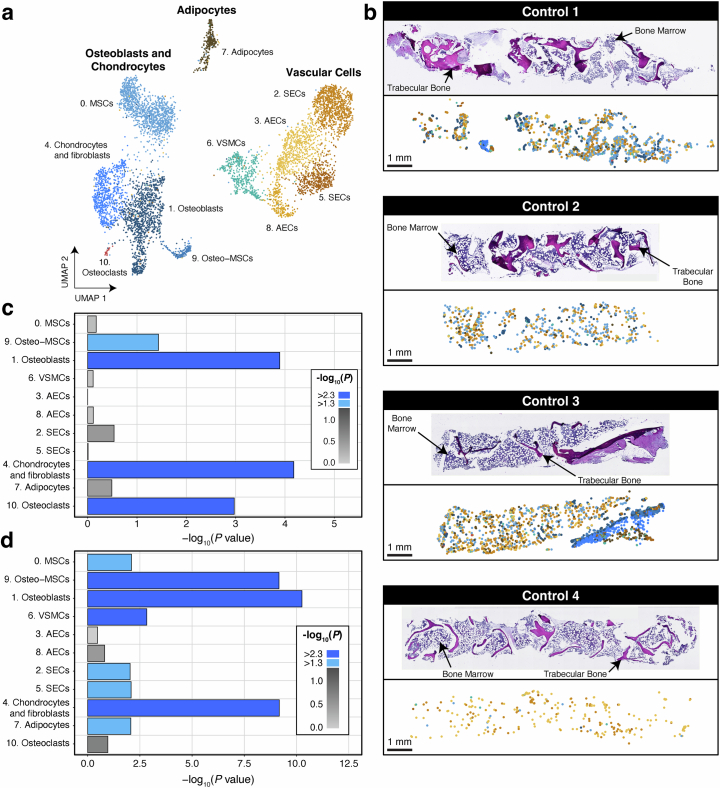
Spatial transcriptomics of human bone samples. (**a**) UMAP plot of non-hematopoietic cell types and osteoclasts identified in trephine biopsies of 4 control patients as identified by Yip and Er *et al*.^50^. MSCs = mesenchymal stromal cells; VSMCs = vascular smooth muscle cells; SECs = sinusoidal endothelial cells; AECs = arterial endothelial cells. (**b**) Haematoxylin and eosin (H&E) images and spatial plots of individual samples used for spatial transcriptomics. Scale bars = 1 mm. Spatial plots display only non-hematopoietic cells and osteoclasts for clarity and are coloured according to (**a**). Bar plots showing enrichment of gene programs as determined by one-tailed Fisher’s exact test under the hypergeometric distribution. for (**c**) causative genes of monogenic skeletal disorders and (**d**) eBMD-associated genes. Scale bars in bar plots indicate the *P* value. Light blue bars in bar plot correspond to observations that have nominal evidence of enrichment: *P* < 0.05, −log_10_(*P*) of >1.3. Dark blue bars in bar plot correspond to observations that have robust evidence of enrichment and meet the *Bonferroni*-corrected significance threshold: *P* < 1.6 ×10^−3^, −log_10_(*P*) > 2.3.

To validate our human scRNA-seq data we interrogated a spatial transcriptomic dataset of adult human bone^50^. In addition to osteoblasts, chondrocytes, cells of the vasculature and osteoclasts, this also identified a population of adipocytes (Extended Data Fig. 10a,b and Supplementary Table 2). The gene programs that define osteoblasts, chondrocytes and osteoclasts were all enriched for genes that caused monogenic skeletal disorders and eBMD-associated genes (Extended Data Fig. 10c,d and Supplementary Tables 6 and 7). In common with the scRNA-seq data, VSMCs were also enriched for eBMD-associated genes (Extended Data Fig. 10d and Supplementary Table 7). Adipocytes were not enriched for monogenic skeletal disorders genes or eBMD-associated genes. Together this demonstrated our approach has important translational potential.

### A platform for data interrogation

Our analysis provides a systematic approach to identify the cells and genes with functional roles in the skeleton. We developed a platform (www.musculoskeletal-genomics.org) that integrates these data and enables users to identify effector genes and define the cells in which they may function.

## Discussion

Advances in understanding the pathophysiology of many diseases has benefited from single-cell technologies. Despite the burden of skeletal disease, the challenges of working with mineralized tissues has meant that skeletal research has lagged behind other fields. We addressed this by developing single-cell methodologies to define the critical endosteal compartment that regulates bone turnover. We then exploited cross-species analysis to identify new cellular and genetic determinants of bone structure and function.

This study identified the cells within the endosteal compartment and defined the gene programs that control them. Cell types enriched for rare monogenic human disorder genes and genes associated with eBMD were considered disease-relevant and investigated for evidence of functional involvement. Our analysis validated the key role of osteoblast, chondrocyte and monocyte/macrophage/osteoclast lineages but also identified a role for underappreciated cell types, including ECs and VSMCs. Many of these cell types were localized to the endosteal compartment illustrating that this is a distinct location within bone.

A role for ECs and VSMCs is exemplified by the finding of a new vascular phenotype in the cortical bone of *Pls3*^*y*/−^ mice and during *pls3*-deficient zebrafish embryo development. The strength of our approach is highlighted by the fact that mutations in *PLS3* are known to cause X-linked osteoporosis, but the cellular and molecular mechanisms have remained elusive^38^. Furthermore, most genes defining disease-relevant cells have underappreciated roles in skeletal pathophysiology. We demonstrate causation for exemplar genes by characterizing skeletal phenotypes in knockout (KO) mice. Our results suggest that pathological mechanisms may be lineage-restricted, involve contributions from multiple cell types or may reflect complex systemic effects. To investigate further, we characterized the interactions between cells and showed that ECs and VSMCs can interact directly with osteoblast lineage cells. This provides a potential explanation for how genes expressed in ECs and VSMCs, such as *Pls3*, *Nherf2* and *Cbx6*, regulate bone.

Our approach generated a comprehensive map of cell types across the endosteal compartment and expands the repertoire of cells that regulate bone to include ECs and VSMCs. This complements analyses of specific bone and marrow cell populations^51–54^.

Our study has limitations. We focused on the endosteal compartment, and this does not include the periosteum. Nevertheless, mesenchymal cells from these locations are similar^55^. The single-cell methodology may not capture fragile cells such as adipocytes, osteoclasts and cells embedded within bone, such as mature osteocytes. Of note, some cells can fragment during disaggregation and remnants can be associated with unrelated cells^56,57^. Despite this, cells with canonical osteoclast markers, probably mononuclear osteoclasts or osteomorphs were identified^25^. Early osteocytes were also isolated, which probably represent cells before entombment. Moreover, the application of spatial transcriptomics validated our approach and enabled identification of adipocytes, although these cells were not enriched for disease-causing genes. We only studied the endosteal compartment in the femur and while different bones have different developmental patterns, osteoblastic cells and osteocytes from different bones express similar gene programs^22,58^. The eBMD GWAS was restricted to individuals with European ancestry and has not been replicated in an independent cohort. Finally, while we phenotyped more than 1,000 KO mouse lines, this did not include all candidate genes. Moreover, because of the scale, outlier phenotypes were determined by comparison of 2–6 mice from each KO line with data from 320 WT littermates. Outliers were independently identified using permutation testing and all lines selected for further study replicated the phenotype, confirming the strength of this approach. Future studies could explore cell specificity and sexual dimorphism using inducible conditional gene targeting.

In summary, our approach integrates genetic, transcriptional and functional data from humans, mice and zebrafish, and identifies a new cellular and molecular framework through which to understand skeletal physiology and disease. These data have implications for skeletal development, rare bone disorders, aging and skeletal responses to systemic diseases, including malignancy and inflammation. This foundational knowledge will help identify therapeutic targets to treat musculoskeletal diseases.

## Methods

Full details of methods, reagents and protocols are provided in Supplementary Note 2.

### Ethical approval

All studies were approved by the appropriate institutional ethics committees and comply with the ARRIVE^59^ and the 2024 Declaration of Helsinki^60^ guidelines.

### Samples for scRNA-seq

Mouse endosteal and BM cells were isolated from femurs of 9–10-week-old WT C57BL/6J male mice obtained from Australian BioResources. Animal experiments were approved by the Garvan Institute of Medical Research Animal Ethics Committee (ARA16/01, ARA19/09 and ARA22/12). Marrow was flushed with PBS; marrow-depleted bone was digested with 2 mg ml^−1^ collagenase A (cat. no. 10103586001, Merck) and 2.5 mg ml^−1^ trypsin (cat. no. T1426, Merck) for 30 min at 37 °C to retrieve endosteal cells. Human femoral head samples were obtained from patients undergoing surgery for osteoarthritis at St. Vincent’s Hospital Sydney. All participants provided written informed consent; the study was conducted with approval from the St. Vincent’s Hospital Human Research Ethics Committee (no. 2022/ETH00475). Bone cores and fragments were excised and digested using 2 mg ml^−1^ collagenase A or collagenase IV (cat. no. LS004186, Worthington Biochemical) and 2.5 mg ml^−1^ dispase (cat. no. D4693 Merck) for 30 min at 37 °C to release cells. Patient and sample characteristics are shown in Supplementary Table 11.

### Fluorescence-activated cell sorting enrichment

Fluorescence-activated cell sorting was performed on mouse and human samples. Viable mouse cells (4′,6-diamidino-2-phenylindole (DAPI)^−^; cat. no. D1306, Thermo Fisher Scientific) negative for erythroid marker Ter119 (cat. no. 116208, BioLegend) were sorted. Viable human cells (DAPI^−^) negative for erythroid marker CD235a (cat. no. 563810, BD Biosciences) were sorted into CD45^+^ (hematopoietic; cat. no. 641408, BD Biosciences) and CD45^−^ (nonhematopoietic) populations. Gating is shown in Supplementary Note 3.

### scRNA-seq and data analysis

Cells were processed using 10X Chromium and Chromium Single Cell 3’ v2 Reagent Kit (target 10,000 cells per channel), and sequenced on the Illumina NovaSeq 6000 sequencing system. Raw data were processed with CellRanger (v.2-7) and analyzed using Seurat (v.2-5)^61^. Log normalization was performed and low-quality cells were filtered (genes < 300 or mitochondrial unique molecular identifier (UMI) > 10%). Dimensionality reduction used principal component analysis (mouse: 40 principal components; human: 20 principal components) on the top 3,000 variable genes. Human data were batch-corrected using Harmony^62^. Clustering used the Louvain method on shared nearest neighbor graphs and visualized using UMAP. Cell types were annotated using canonical markers and cluster-specific gene programs (Supplementary Note 1).

### Defining cell-type gene programs

Differentially expressed genes (gene programs) were identified using Seurat FindAllMarkers, requiring log_2_(fold change) > 0.5 and Bonferroni-adjusted *P* < 0.05 (Supplementary Table 2). The restrictedness of gene expression was calculated by determining the number of clusters expressing a given gene. A cluster was deemed to express a gene if one or more UMIs for that gene were detected in at least 25% of cells within the cluster. Gene programs are shown in Supplementary Table 2.

### Cell–cell interaction analysis

Cell–cell interactions were identified using CellPhoneDB (v.5.0.1)^63^ with default settings. Signaling pathways were analyzed using interrogating ReactomeDB^64^ for enriched terms using ReactomePA^65^.

### Reconstruction of cell differentiation trajectories

Differentiation trajectories were reconstructed using the Monocle package (v.2) (discriminative dimensionality reduction tree)^66^ on the top 1,000 differentially expressed genes.

### Comparing mouse and human scRNA-seq datasets

Label transfer analysis (Seurat v.5.3.0) was performed to compare mouse and human datasets. TransferData with 20 principal components was used; prediction scores > 0.5 indicated high-confidence transfers^67^.

### Identifying TFs regulating gene programs

TFs and their target genes (regulons) were identified using pySCENIC (v.0.11.2)^68^. Gene regulatory network inference used GRNBoost2 and cisTarget; regulon activity was scored using AUCell.

### snATAC-seq

Endosteal nuclei were isolated from mouse bone and processed using the 10X Genomics Chromium Single Cell ATAC v2 platform. Sequencing was performed on an Illumina NovaSeq 6000 (∼25,000–50,000 fragments per nucleus). Raw data were processed with CellRanger ATAC (v.2.1.0) and analyzed using Signac (v.1.15.0)^69^ and Seurat. Low-quality nuclei were filtered. Data were normalized (term frequency-inverse document frequency), reduced (singular value decomposition) and clustered (shared nearest neighbor/UMAP). Gene activity scores were computed and integrated with scRNA-seq via canonical correlation analysis. Differential accessibility was assessed using logistic regression and enriched TF-binding motifs identified using FindMotifs.

### Flow cytometry validation

Flow cytometry analysis was performed to validate enrichment of endosteal populations. Endosteal cells were isolated using flushing and enzymatic digestion, stained with Zombie NIR Viability Stain (cat. no. 423106, BioLegend), B220-BV510 (cat. no. 103248, BioLegend), TCRβ-BV510 (cat. no. 118131, BioLegend), CD45-BV650 (cat. no. 103151, BioLegend), CD11b-BUV395 (cat. no. 565976, BD Biosciences), Ly6C-BUV737 (cat. no. 755201, BD Biosciences), Ly6G-APC (cat. no. 560599, BD Biosciences) and CD14-PE (cat. no. 569968, BD Biosciences) and acquired on a BD FACSymphony. Analysis was performed using FlowJo v.10.10.0 (FlowJo LLC).

### Skeletal gene lists

Two foundational lists of skeletal-relevant genes were curated (Supplementary Table 3). GO Biological Processes^70^: genes associated with GO biological processes related to skeletal function. MGI abnormal skeletal phenotype genes: genes identified from the MGI database^71^ (MP:0005390) causing abnormal skeletal phenotypes when mutated in mice.

### Hypergeometric overrepresentation testing

Overrepresentation analyses were performed using a Fisher’s exact test under the hypergeometric distribution (Supplementary Table 12). *P* values were adjusted via Bonferroni correction. All tests used the RITAN package^72^ and standardized human Ensembl IDs (BioMart).

### Gene programs enriched for monogenic skeletal disorder genes

A list of 528 protein-coding genes derived from the International Skeletal Dysplasia Society Nosology database^23^ (Supplementary Table 3) was used with Fisher’s exact tests. Network plots were generated in Cytoscape (v.3.10.0)^73^ to visualize cell-gene expression.

### Genome-wide association analysis of eBMD

UKB participants with European ancestry were identified: genotypes were projected onto the first 20 principal components of the 1000 Genomes Project phase 3 (1KG) individuals using GCTA (v.1.93.2). Principal components 1–20 of UKB and 1KG individuals were projected into three UMAP components with the following parameters: min_dist = 0.000001, n_components = 3, n_neighbors = 35, random_state = 10293082. Parameters were defined manually and informed by clustering 1KG individuals with the same ancestry. A total of 461,920 UKB participants clustered with 1KG individuals with European ancestry. Individuals were mapped back to the principal component analysis plots to ensure they co-localized with 1KG individuals with European ancestry.

As described previously^8^, eBMD was derived in 481,380 UKB participants. A total of 448,010 individuals with European ancestry and rank-based inverse normal transformed eBMD were analyzed using a linear mixed noninfinitesimal model implemented in BOLT-LMM v.2.3.4 with age, sex, genotyping array and principal components 1–20 as covariates. Variants with a minor allele frequency > 0.05% and imputation quality score > 0.3 were analyzed. Manhattan plots were generated using ggplot2 (ref. ^74^).

Population stratification and other latent sources of confounding were investigated using stratified LD score regression implemented in the LDSC Baseline LD model (v.1.0.1) together with the --chisq-max 5,000 flag. The LDSC attenuation ratio statistic (LDSC_RPS_ = 0.02 (s.e. = 0.0195)) suggested that polygenicity accounted for genomic inflation (λ_GC_ = 2.27). The large single-nucleotide polymorphism (SNP) heritability of eBMD (h^2^_SNP_ = 0.39, s.e. = 0.027) and sample size probably resulted in the LDSC intercept exceeding 1 (LD score regression = 1.08, s.e. = 0.073).

SNPTracker (v.1.0) updated reference SNP cluster IDs (rsIDs) and genomic positions from Hg19 to GRCh38 using dbSNP release 151 (ref. ^75^). SnpEff 5.1d^76^ using the GRCh38.p14 database predicted the functional impact of genetic variants. High-impact variants were validated with the variant effect predictor^77^.

eBMD-associated lead variants were identified using GCTA-COJO^78^ (v.1.94.1)^79^. A genome-wide significance threshold of *P* < 6.6 × 10^−9^ was used; variants with high collinearity (*R*^2^ > 0.8) were ignored; those more than 10 MB away were assumed to be in complete linkage equilibrium. Approximately 50,000 unrelated individuals of European ancestry from the UKB were used to model LD^79^.

Lead variants were binned to ‘loci’ using a 1-MB sliding window. Loci were deemed ‘new’ if located more than 1 MB from any variant in the NHGRI-EBI GWAS Catalog associated with bone density ‘EFO_000392’ at *P* < 5 × 10^−8^. Data were downloaded on 5 March 2024; the Hg38 coordinates of variants were obtained using SnpTracker. Genomic distances were calculated using BEDTools (v.2.29.2)^80^. Each lead variants was annotated to the closest protein-coding gene using BEDTools and the Ensemble Genes 105 (GRCh38) dataset from BioMart^81^.

### Gene-based tests of association

MAGMA (v.1.10) gene-based tests were conducted using the eBMD GWAS summary statistics of variants with an info score greater than 0.6 and minor allele frequency > 0.5%. LD was modeled with ~50,000 unrelated UKB individuals of European ancestry. Variants were annotated to protein-coding genes using coordinates from Ensemble Genes 105 (GRCh38). Gene annotations included variants ≤ 2 kb upstream of the transcriptional start site and ≤ 1 kb downstream of the stop site. A multi-model approach was used to derive aggregate *P* values (*P*_Multi_) for gene-based tests. A gene-wide significance threshold was determined using Bonferroni correction: the error rate of one test divided by number of genes tested = 0.05/19,695 = *P* < 2.54 × 10^−6^.

### Enrichment analysis involving eBMD-associated genes

Hypergeometric tests were performed as described above to investigate whether rare skeletal disorder genes were overrepresented among genes located closest to the eBMD lead variants, and separately, among genes associated with eBMD (Supplementary Table 7). Hypergeometric tests were also used to determine if enrichment was attributable to genes that caused individual skeletal dysplasia groups.

### Gene programs enriched for eBMD-associated genes

MAGMA competitive GSA was used to determine whether gene programs were enriched for eBMD-associated genes. GSA accounted for gene size, gene density and the inverse of the mean minor allele count in the gene, as well as the log value of these three factors. The statistical significance threshold was determined using Bonferroni correction: the error rate of a single test divided by the number of gene programs in each experiment (Supplementary Table 7). A post-hoc permutation analysis was conducted using MAGMA. Pairwise conditional GSA was conducted to account for genes that were shared between gene programs of enriched cell clusters and cell subclusters. A conditional *P* value < 0.05 was used to reject the null hypothesis of no enrichment after correcting for shared genes.

### Pulse rate as a negative control for gene enrichment

We analyzed pulse rate as a negative control in the same individuals from the UKB using the same workflow.

### Enrichment in the MGI database

Hypergeometric tests were used to investigate whether genes regulating bone structure were overrepresented among gene programs of cell types that were enriched for monogenic disorder genes or eBMD-associated genes. Genes causing bone structural phenotypes when mutated in mice were identified from the MGI database (MP:0003795, accessed 21 May 2024 (ref. ^71^)). A total of 1,347 protein-coding genes and genes with human orthologs were extracted using BioMart implemented in R^81^ (Supplementary Table 3).

To determine whether genes more highly expressed in each gene program were more strongly enriched for genes that regulate bone structural integrity, we allocated genes to six nested groups based on the magnitude of fold change in gene expression. Enrichment analysis using all genes, and only eBMD-associated genes from each group, was performed.

### Genetically modified mice

Skeletal phenotyping was undertaken at Imperial College (PPL70/8785 and PP1540664) and the Wellcome Trust Sanger Institute (WTSI) Mouse Genetics Project (MGP) as part of the International Mouse Phenotyping Consortium (IMPC) (PPL nos. 80/2485 and P77453634).

### *Pls3* KO mouse line

C57BL/6N mice carrying a *Pls3*^*tm1a(EUCOMM)Wtsi*^ KO first allele (MGI:104807) were obtained from the WTSI EMMA mouse repository and rederived from frozen embryos. The skeletal phenotype was studied in hemizygous male (*Pls3*^*y*/−^) mice compared to WT (*Pls3*^*y*/+^) male littermates, and in female *Pls3*^−/−^ mice.

### WT and mutant mice generated by the IMPC

Samples from 16-week-old WT and genetically modified mice on C57BL/6 backgrounds were generated by the WTSI MGP. All mice underwent a primary phenotype screen including X-ray skeletal survey and biochemical measures of mineral metabolism.

### OBCD bone phenotyping

Female 16-week-old mice from WTSI MGP lines were euthanized and phenotyped to determine 19 parameters of bone mass and strength^7,8,42^. Phenotype definitions were based on structural (difference in X-ray or micro-CT parameters), functional (difference in biomechanical strength), structural and functional (difference in both), bone quality (functional outlier not correlating with BMC) or Mahalanobis (significant outlier because of small differences in multiple parameters).

### Digital X-ray microradiography

Digital X-ray images were recorded for fixed hindlimbs and caudal vertebrae (Ca6, Ca7) at 10-μm resolution using a Faxitron MX20 variable kV point projection X-ray source and digital imaging system (Qados, Cross Technologies) to determine relative BMC, a two-dimensional parameter similar to areal BMD^82^.

### Micro-CT

The three-dimensional cortical and trabecular structural parameters of femurs were scanned using a SCANCO μCT50 (SCANCO Medical) at 70 kV, 200 μA with a 0.5-mm aluminum filter. Cortical parameters were calculated from 10-μm voxel resolution scans in the mid-shaft (ROI centered at 56% length). Trabecular parameters were calculated from 5-μm voxel scans of a 1-mm region proximal to the distal femoral growth plate. For male *Pls3*^*y*/−^ mice, femurs and L5 lumbar vertebrae were imaged. High-resolution 1-μm scans were performed on the mid-shaft to determine cortical porosity and canal diameter^22^. Analysis was performed using the SCANCO Medical software suite. Quantification of vascular and osteocyte lacunae was performed on P112 males. Volume distributions were compared using Kolmogorov–Smirnov analysis.

### Biomechanical testing

Destructive three-point bend tests were performed on femurs; two-point compression tests were performed on caudal vertebrae (Ca6, Ca7) using an Instron 5543 as described^7,83^. Load displacement curves were used to calculate yield, maximum and fracture loads, and stiffness.

### Histology and histomorphometry

Whole-mount skeletal staining of P1 neonates was performed using Alizarin red (mineralized bone) and Alcian blue (cartilage)^84^.

Growth plate histomorphometry was conducted on Alcian-blue-stained and van Gieson-stained sections of decalcified tibias^85^, measuring the width of the reserve, proliferative and hypertrophic zones using ImageJ.

Osteoclast static histomorphometry was performed on decalcified proximal humeri stained for tartrate-resistant acid phosphatase^86^, and histomorphometry analysis performed using the TrapHisto software (www.liverpool.ac.uk/ageing-and-chronic-disease/bone-hist/trap-hist/) in a 750 × 750 µm area 250 µm distal to the proximal humeral growth plate^86,87^.

Osteoblast dynamic histomorphometry was performed on mice double-labeled with calcein (cat. no. C0875, Sigma-Aldrich) at 6 and 2 days before collection. Calcein fluorescence was imaged using confocal scanning light microscopy on methacrylate-embedded femurs and lumbar vertebrae to quantify total bone surfaces, labeled surfaces and separation between double-labels according to the American Society for Bone and Mineral Research nomenclature^87,88^.

CD31 immunohistochemistry was performed on decalcified P21 femurs using a primary rabbit anti-CD31 antibody (cat. no. ab182981, Abcam) to quantify vascular canals in cortical bone.

### Iodine-contrast-enhanced backscattered electron-SEM

Formalin-fixed P70 male femurs were embedded in polymethyl methacrylate and stained with Lugol’s Iodine solution. Blocks were carbon-coated and imaged using SEM (Tescan) at high vacuum using a four-quadrant backscattered electron detector. Cortical bone ROIs were analyzed blind using ImageJ to determine the total cortical area and the number and area of vascular canals.

### Serum analysis

Serum markers for bone resorption (CTX) and formation (P1NP) were determined using enzyme-linked immunosorbent assay (AC-06F1 and AC-33F1, Immunodiagnostic Systems) at P70. Wnt inhibitors (DKK-1, SOST; cat. nos. MKK100 and MSST00, R&D Systems) were determined at P183.

### Statistical analysis of skeletal phenotypes

IMPC lines were compared to WT reference ranges from two different genetic backgrounds (320 C57BL/6N and C57BL/6NTac mice; 80 C57BL/6Brd-Tyrc-Brd and C57BL/6Dnk mice) using reference range analysis and permutation testing. Multivariate analysis was performed using squared robust Mahalanobis distances (MDi2) to identify significant abnormal phenotypes resulting from simultaneous small variances across the 19 skeletal parameters. Bone quality outliers were identified using 95% prediction intervals from BMC versus strength linear regression. *Pls3*^*y*/−^ data were compared using an unpaired Student’s *t*-test (*P* < 0.05). The Kolmogorov–Smirnov test was used to compare cumulative frequency distributions of BMC^42,89^.

### Zebrafish husbandry and genome editing

Zebrafish experimentation adhered to guidelines of the animal ethics committee of the University of Queensland (permit no. 2022/AE000091). The *Tg(kdrl:EGFP)*^*s843*^ zebrafish transgenic line was used for all studies^90^. Embryos were obtained through natural paired matings and incubated at 28 °C in a dark-phase incubator. Embryos were maintained in 10-cm Petri dishes containing 1× E3 media at a maximum density of *n* = 60. At 24 hpf, all embryos were changed to E3 medium supplemented with 0.0003% phenylthiourea to prevent pigmentation.

### Genome editing of *pls3* mutants in zebrafish

G0 mosaic crispants were generated by injecting ∼1 nl of ribonucleoprotein mix containing Alt-R Cas9 Nuclease and three CRISPR RNAs (details in Supplementary Note 2 and Supplementary Table 13) targeting *pls3* at the one-cell stage.

### Confocal imaging

Live zebrafish embryos were mounted laterally and imaged on a Zeiss LSM 710 confocal microscope. ISV luminal diameter was quantified using a VasoMetrics macro in ImageJ^91^.

### Non-osseous tissue gene expression analysis

Gene expression across non-osseous tissues was interrogated using processed scRNA-seq data from the TabulaMuris^92^ and TabulaSapiens^93^ databases. Cell clusters with fewer than ten cells were excluded. Remaining clusters were manually assigned to a cell group using the Human Protein Atlas classification system. Mean raw expression of selected exemplar genes within each cluster was calculated and visualized.

### Statistics and reproducibility

Details for individual experiments are included in each corresponding section in the Methods.

For the scRNA-seq analysis, no statistical method was used to predetermine sample size. Cells were isolated from five independent experiments, each with five biological replicates and combined for subsequent analysis. For snATAC-seq, a single experiment was performed.

For the genetic association analyses, participants were excluded if they had low-quality genomic and outcome data or were missing outcomes and covariates pertinent to the association testing. Obvious outliers were manually filtered by observing the distributions of the data and removing individuals far exceeding the tail ends of the data. Low-quality SNPs were excluded from the analysis in accordance with standard quality control criteria. Multiple-hypothesis correction was performed using Bonferroni correction.

In the OBCD mouse KO studies, sample size was determined as detailed in Supplementary Note 2. The coefficient of variation for each of the OBCD skeletal phenotyping parameters was defined using 400 WT mouse samples from two different genetic backgrounds. Power calculations indicate an 80% power to detect an outlier phenotype of greater or equal to 2 s.d. with a sample size of two for each line in the OBCD screen. Data for all mice were included, including those with and without skeletal phenotypes; no data were excluded. For the analysis of *Pls3*^*y*/−^ mice, mice with gene deletion were compared to littermate controls. Power calculations using WT data together with the magnitude of the skeletal phenotype identified in female *Pls3*^−/−^ mice in the OBCD screen (*n* = 2) demonstrated that *n* = 7–8 was required. Investigators were blinded to genotypes during analysis of skeletal phenotypes. Unblinding occurred at the point of statistical analysis.

### Reporting summary

Further information on research design is available in the Nature Portfolio Reporting Summary linked to this article.

## Online content

Any methods, additional references, Nature Portfolio reporting summaries, source data, extended data, supplementary information, acknowledgements, peer review information; details of author contributions and competing interests; and statements of data and code availability are available at 10.1038/s41588-026-02640-9.

## Supplementary information

Supplementary Information Supplementary Table 1, Figs. 1–10 and Notes 1–3.

Reporting Summary

Peer Review File

Supplementary Table 2–13 All supplementary tables

## Source data

Source Data for Figs. 1 and 4–7, and Extended Data Fig. 9.

## Data Availability

The scRNA-seq and snATAC-seq raw data are available on the Gene Expression Omnibus (GEO) under accession no. GSE317069. Human spatial transcriptomic data generated by Yip et al.^50^ are available on the GEO under accession no. GSE299207. Genome-wide association summary result statistics are available via the NHGRI-EBI GWAS Catalog (https://www.ebi.ac.uk/gwas) under accession no. GCST90726625. The interactive web portal can be accessed at www.musculoskeletal-genomics.org. Source data are provided with this paper.

## Code availability

All single-cell transcriptomic and genomics analyses were performed using publicly available software as described in the Methods and Supplementary Note 2. Custom scripts are available at GitHub (https://github.com/ryacha83/Single-cell-RNA-seq-analysis-of-mouse-and-human-bone-and-genomic-analysis-of-eBMD) and Zenodo (10.5281/zenodo.19624990)^94^.
