# Supplementary material for: Multiscale analysis and functional validation of the cellular and genetic determinants of skeletal disease
Source: Nat Genet. 2026 Jul 10;58(7):1530–45. doi: 10.1038/s41588-026-02640-9 (PMC13364720; doi:10.1038/s41588-026-02640-9)
Supplement: Supplementary file 1 — Supplementary Table 1, Figs. 1–10 and Notes 1–3. [file 41588_2026_2640_MOESM1_ESM.pdf]

nature genetics

Supplementary information https://doi.org/10.1038/s41588-026-02640-9

# Multiscale analysis and functional validation of the cellular and genetic determinants of skeletal disease

In the format provided by the authors and unedited

**SUPPLEMENTARY INFORMATION**

Supplementary Table 1

Supplementary Figures 1-10

Supplementary Notes 1-3

**Supplementary Table 1 - Glossary of Terms and Definitions:**

This glossary defines key terms used in this manuscript. Detailed technical information is provided in Supplementary Note 2.

| Term | Definition |
|---|---|
| Endosteal bone compartment | The layer of cells located at the interface between bone and the bone marrow. Includes cells that are adjacent to bone and those up to 10 cells away from the bone surface. |
| Bone marrow compartment | The cells in the bone marrow space that are not adjacent to bone and are not part of the endosteal bone compartment. |
| Cluster | A group of cells that share similar patterns of gene expression. Cluster membership is based on the magnitude of expression and restrictedness of ~3000 highly variable genes |
| Restrictedness | A term used to describe whether a gene is expressed in a single cluster or found in more than one cluster. |
| Gene program | The set genes that are differentially upregulated in a given cluster relative to all other cells in the dataset. |
| Pathogenic variant | A genetic variant that changes the function of a gene in a way that causes a monogenetic disorder. |
| Monogenic | A trait or disorder resulting from pathogenic variants that occur within a single gene. |
| Causative gene | A gene that causes a rare monogenetic disorder when its function is altered by a pathogenic variant. |
| Nosology | A classification of individual monogenetic disorders caused by pathogenic variants in single genes. |
| Causal variant | A genetic variant that changes the function of a gene in a way that alters a polygenetic trait and/or susceptibility to disease. |
| Polygenic | A trait or disorder that result from the contribution of many independently acting or interacting causal genetic variants. |
| Effector gene | A gene that causes a change in a polygenetic trait and/or susceptibility to disease when its function is altered by causal variants. |
| Abnormal bone structure from MGI | Anomaly in the composite material or the layered arrangement of the bony endoskeleton of the body |

| | |
|---|---|
| Gene set enrichment analysis | A family of statistical methods that can be used to identify whether a set of genes are over-represented in a gene list e.g. gene program. |
| High impact variant | A genetic variant that is predicted to have a disruptive impact on the protein, causing protein truncation, loss of function or triggering nonsense mediated decay. |
| Moderate impact variant | A genetic variant that is non-disruptive, but likely to change protein expression level and/or function. |
| Low impact variant | A genetic variant predicted to be mostly non-deleterious or unlikely to change protein expression level and/or function. |
| Locus | A region in the genome that contains genetic variants that are associated with variation of a polygenetic trait and/or susceptibility to disease. |
| Lead variant | The genetic variant that is most strongly associated at a locus after accounting for all other associated genetic variants. |
| Structural phenotypes | Significant difference in bone structural parameters measured by digital X-ray microradiography or micro-computerised tomography. |
| Functional phenotypes | Significant differences in mechanical strength parameters measured by biomechanical testing |
| Structural and functional phenotypes | Significant differences in at least one structural and one functional parameter |
| Bone quality phenotypes | A term that encompasses various structural parameters of bone that affect fragility, including bone microarchitecture, mineralization, and material properties [Grynpas, M. D. (2003). The role of bone quality on bone loss and bone fragility. In Bone loss and osteoporosis: An anthropological perspective (pp. 33-44). Boston, MA: Springer US.]. In the OBCD study, it refers to lines with outlier functional phenotypes that did not correlate with bone mineral content |
| Mahalanobis phenotypes | Lines that were significant outliers due to smaller differences in multiple skeletal parameters. |

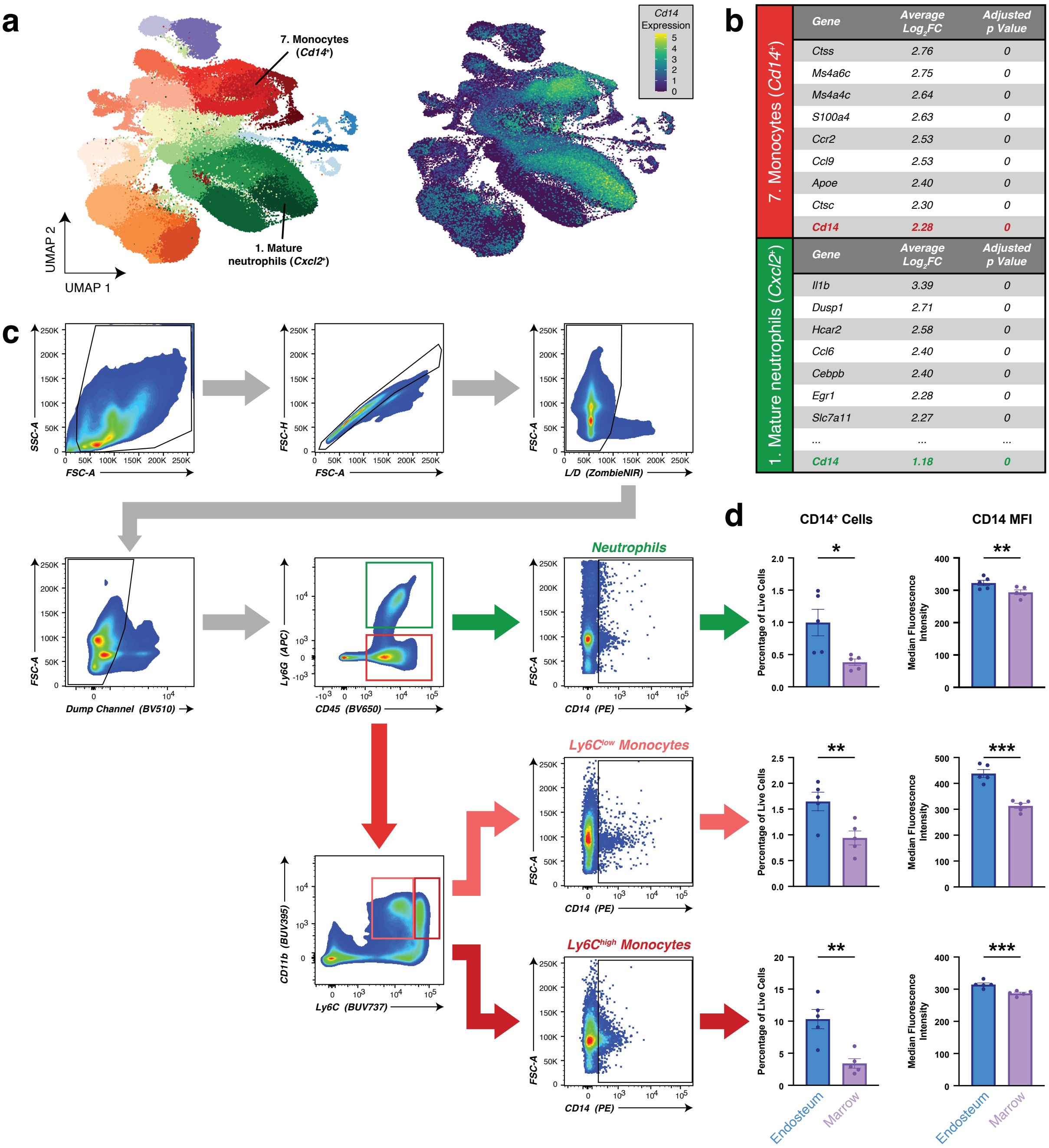


**Supplementary Fig. 1. Validation of cell populations enriched in the endosteal compartment of mouse bone.** (a) UMAP plots displaying expression of *Cd14* within the mouse scRNA-seq dataset. Clusters “7. Monocytes (*Cd14*+)” and “1. Mature neutrophils (*Cxcl2*+)” are indicated. (b) Top marker genes for clusters “7. Monocytes (*Cd14*+)” and “1. Mature neutrophils (*Cxcl2*+)”. *Cd14* is highlighted as a strong marker for both clusters. (c) Gating strategy used to identify neutrophils and monocyte populations in mouse bone marrow and endosteal samples and to determine CD14 expression within these populations. (d) Quantification of CD14 expression in selected populations. Left bar plots show the proportion of cells with detectable CD14 expression. Right bar plots show median fluorescence intensity of CD14 within selected populations. Mean±s.e.m are shown; two-tailed Students’ *t*-test; *** $P<0.001$, ** $P<0.01$, * $P<0.05$.

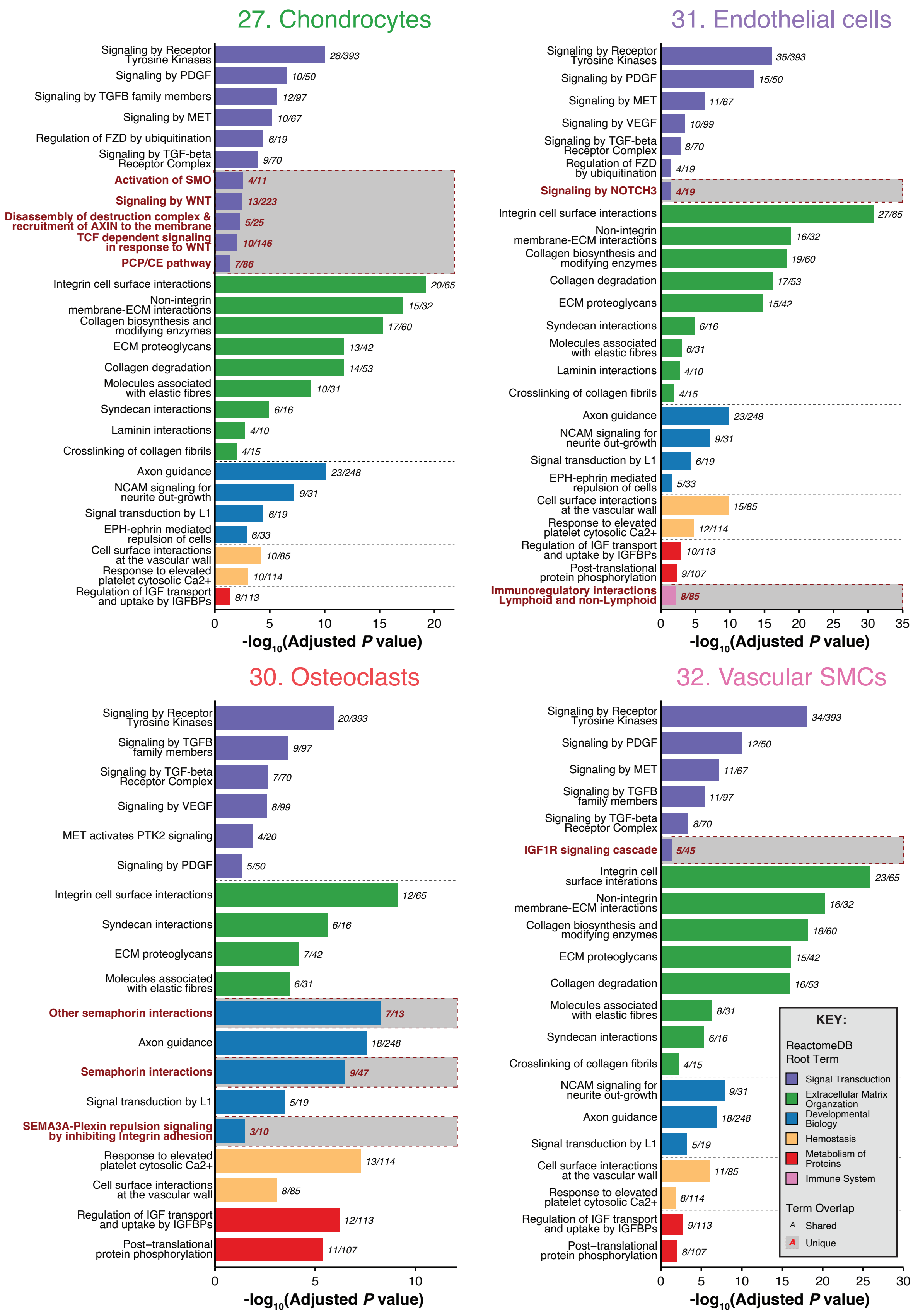


**Supplementary Fig. 2. Cell-cell interaction of osteoblast lineage cells with other non-haematopoietic cells.** Bar plots displaying enriched ReactomeDB pathways identified amongst interactions between osteoblast lineage cells and selected cell types. Pathways are

grouped according to their root term within the ReactomeDB database (see Methods). Adjoining numbers indicate the proportion of genes associated with a term that were present within the results. Terms that are uniquely enriched for one cell type are indicated in grey boxes and red outlines/text.

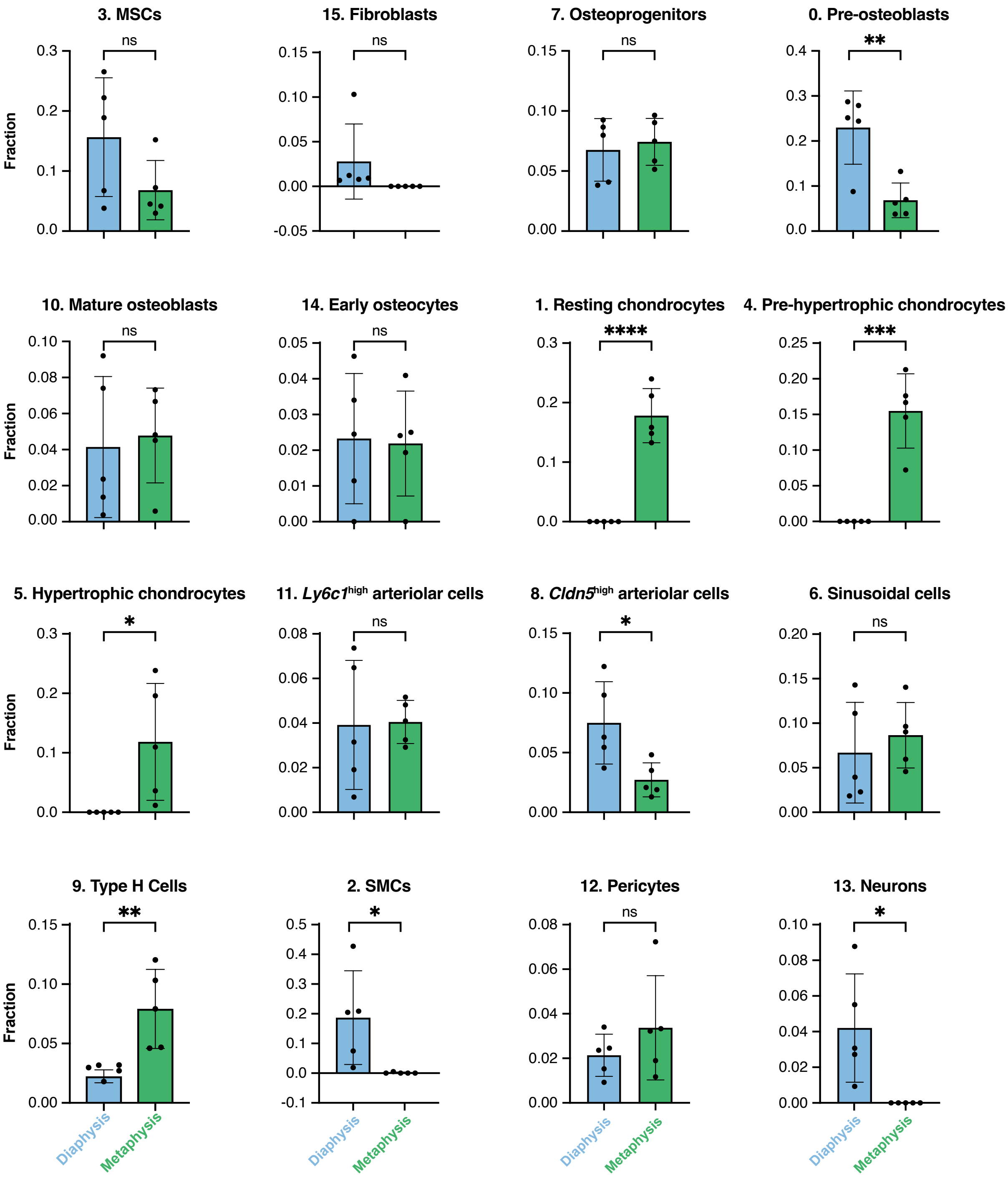


**Supplementary Fig. 3. Distribution of non-haematopoietic cell sub-clusters in diaphysis and metaphysis.** Bar plots showing the fraction of all non-haematopoietic sub-clusters in diaphysis and metaphysis. Mean +/- s.e.m are shown; two-tailed Students' *t*-test; **** $P$<0.0001, *** $P$<0.001, ** $P$<0.01, * $P$<0.05.

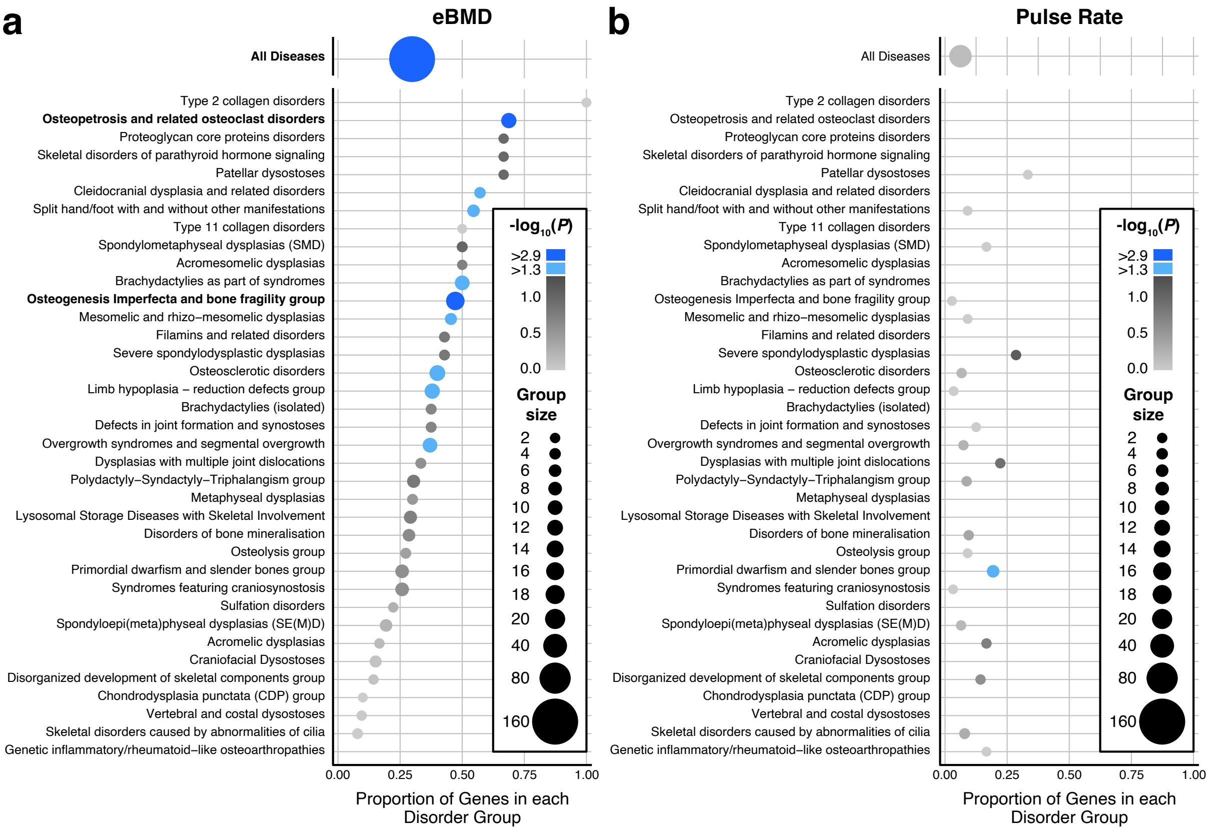


**Supplementary Fig. 4. Genes associated with eBMD, but not pulse rate, are enriched for monogenic skeletal disorder genes.** Bubble plot showing the number, and proportion of monogenic skeletal disorder genes from each disorder group that are present in the set of protein-coding genes associated with (a) eBMD and (b) pulse rate. Size of the circles represent the number of genes in each disorder group present within the gene list. Scale bar indicates the *P* value of enrichment. Light blue dots indicate nominal evidence of enrichment: *P* value of <0.05 [-$\log_{10}$(*P* value) of >1.3]. Dark blue dots denote robust evidence of enrichment meeting the *Bonferroni*-corrected threshold of $P<1.2 \times 10^{-3}$ [-$\log_{10}$(*P* value) of >2.9]. All statistics are determined by one-tailed Fisher's exact tests under the hypergeometric distribution.

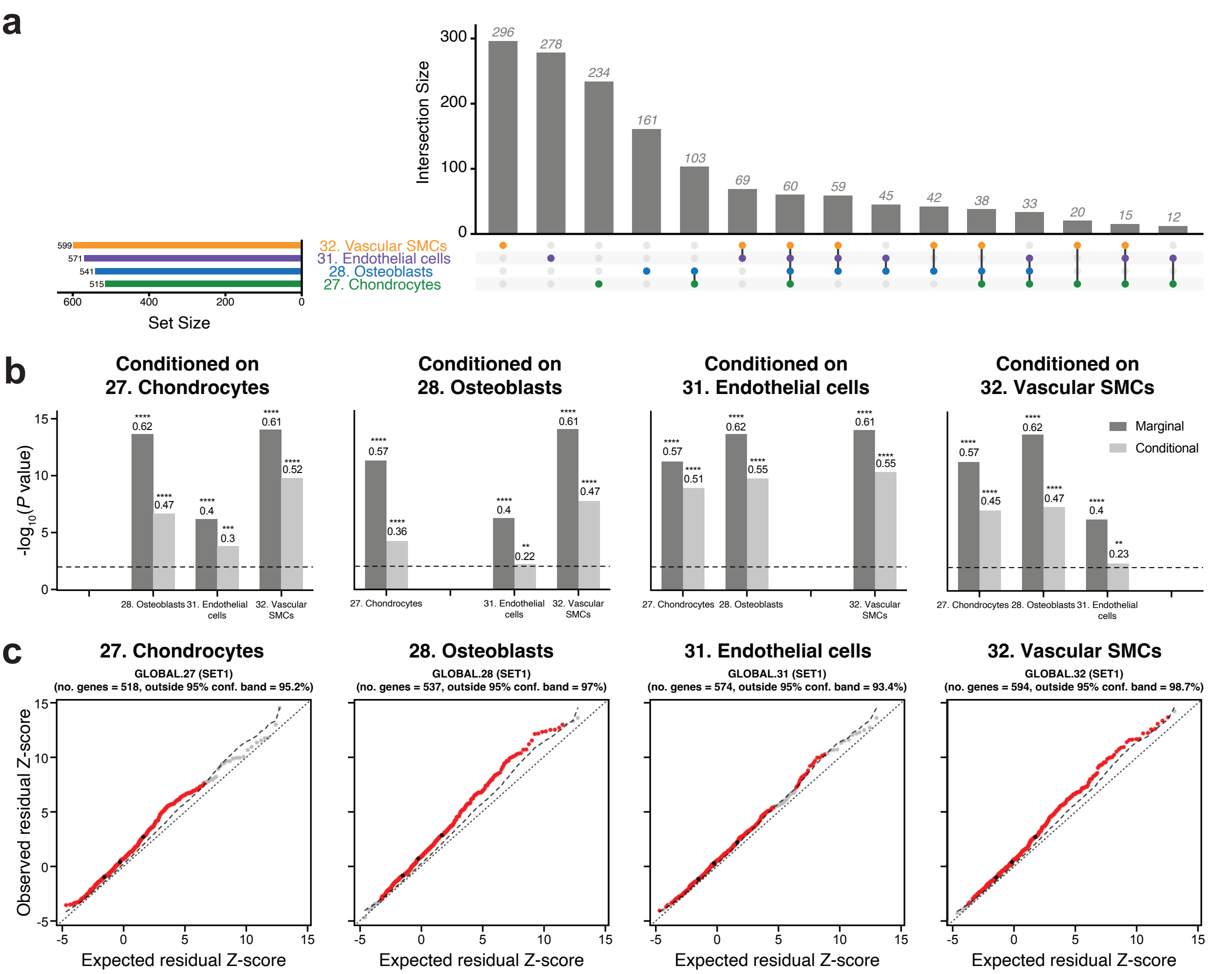

**Supplementary Fig. 5. Conditional GSA analysis to determine whether enrichment was confounded by shared sets of genes.** (a) UpSet plot showing the number of restricted and shared genes associated with eBMD in non-haematopoietic cell types. (b) Histograms showing a pairwise conditional GSA analysis used to determine whether enrichment was confounded by shared sets of genes. Each histogram quantifies the strength of evidence of enrichment. Dark grey bars correspond to strength of evidence of enrichment in marginal (original) analyses and light grey bars correspond to the strength of evidence of enrichment after adjusting for the effect of genes that are shared between two cell types (conditional analyses). Numbers above each bar correspond to the GSA point estimate (i.e. β). **** *P*<0.001, *** *P*<0.005, ** *P*<0.01, * *P*<0.05. Dotted line corresponds to the threshold of statistical significance (*P*<0.05). (c) Post-hoc permutation analyses showing QQ-plots of Z-scores of genes in each gene program of different cell clusters. Plots show residualised Z-scores from the null model for each gene program, with the expected values based on the quantiles across all genes in the data. The black points denote the 25th, 50th and 75th percentile. The dashed black line represents the one-sided (upper) 95% confidence band. Genes are coloured red if they exceed the confidence band, and grey if they do not. The proportion of genes in each program exceeding the confidence band is indicated on each plot.

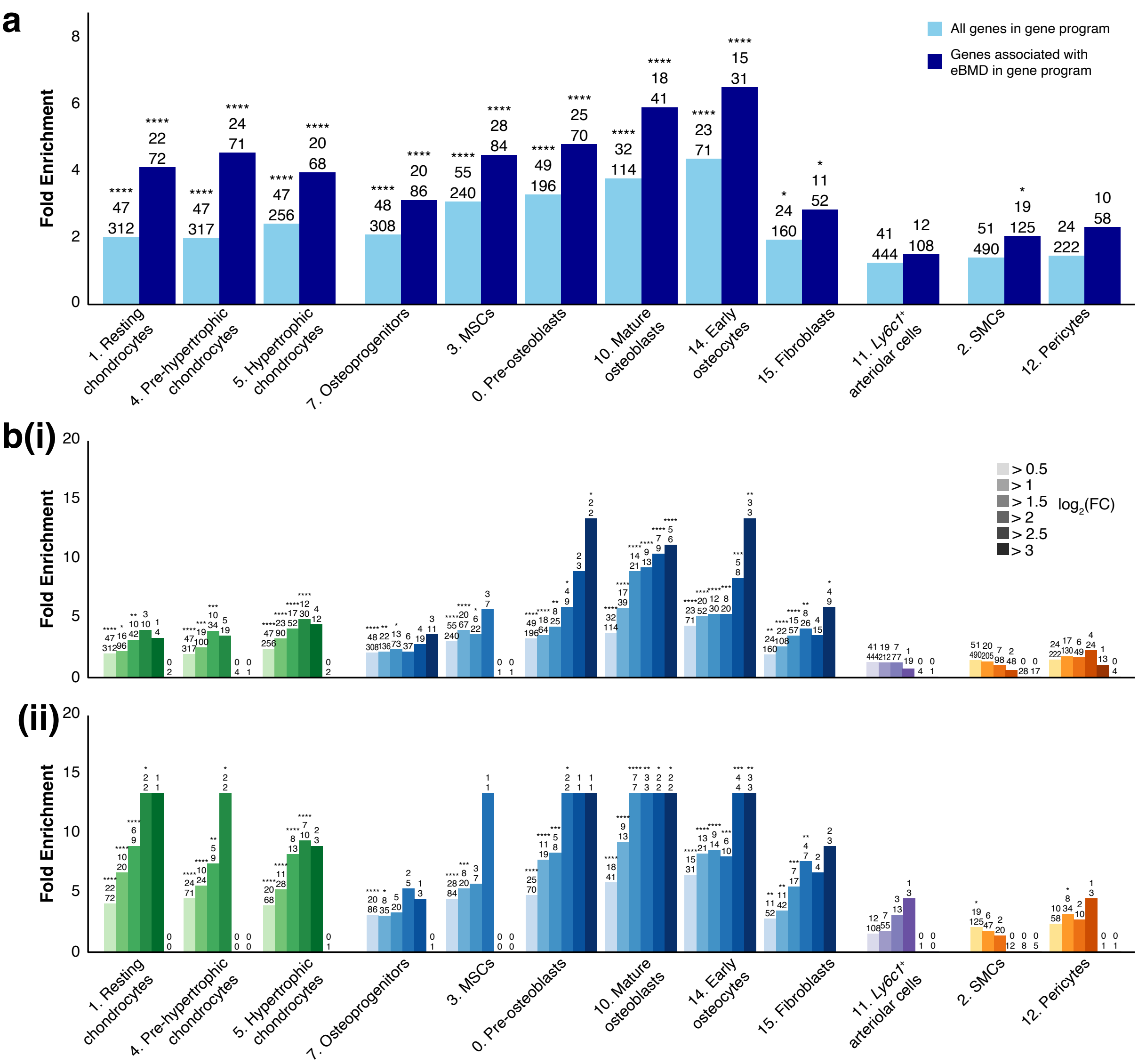


**Supplementary Fig. 6. Gene programs of non-haematopoietic sub-clusters are enriched for genes that cause abnormal bone structure when mutated in mice.** (a) Enrichment of sub-clusters for abnormal bone structure genes defined by MGI. Light blue bars represent all genes in the sub-cluster gene program; dark blue bars represent the subset associated with eBMD. (b) Sub-cluster enrichment stratified by magnitude of gene expression. Panel (i) includes all genes in the sub-cluster gene program, and panel (ii) includes the subset of eBMD-associated genes. *Bonferroni*-corrected significance is indicated. **** $P<0.001$, *** $P<0.005$, ** $P<0.01$, * $P<0.05$. All statistics are determined by one-tailed Fisher's exact tests under the hypergeometric distribution.

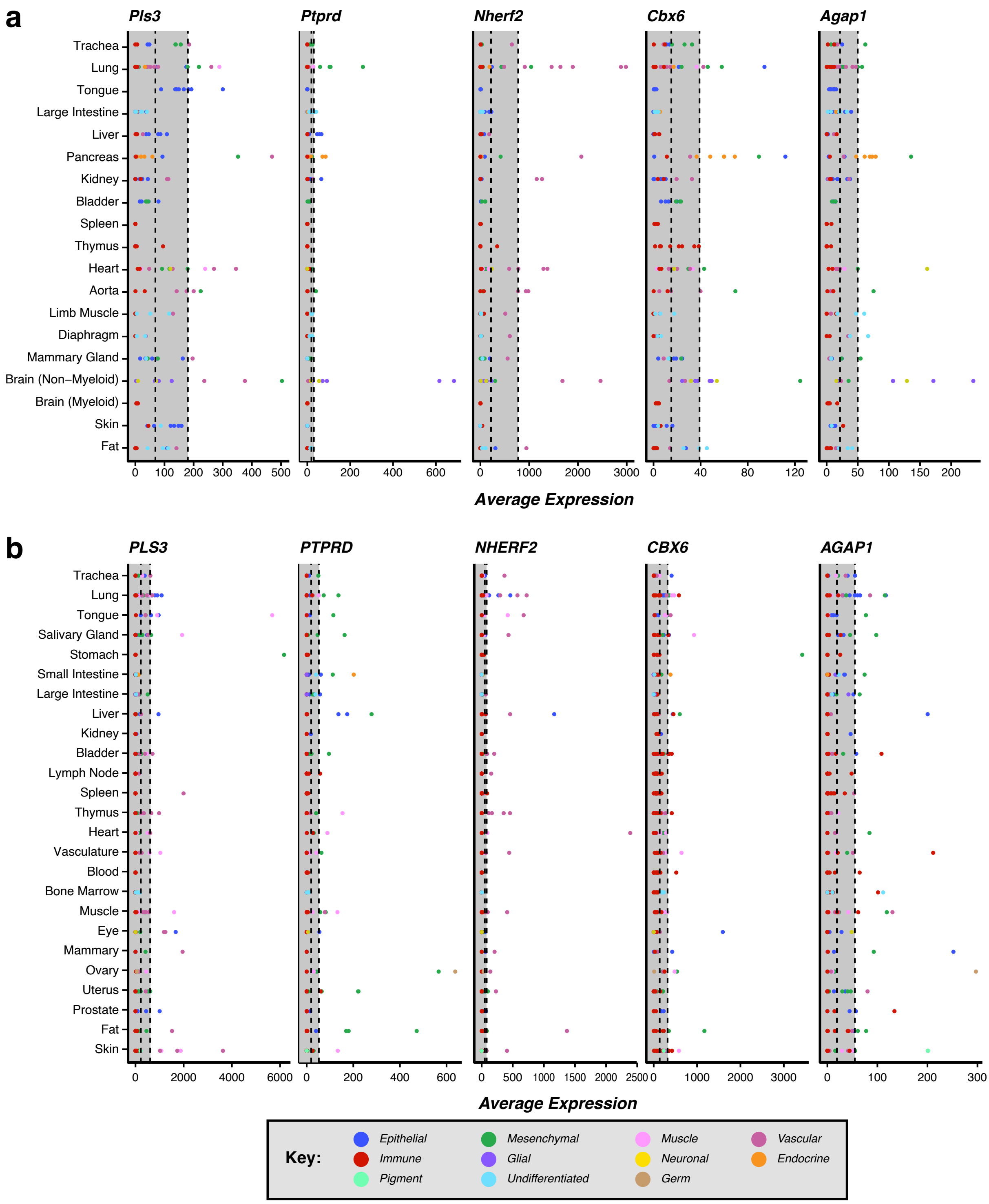


**Supplementary Fig. 7. Expression of exemplar genes in tissues outside of the skeleton.** Dotplots showing expression of exemplar genes across cell types isolated from different mouse (a) and human (b) tissues, using the TabulaMuris and TabulaSapiens datasets respectively. Individual cell types were manually assigned a classification using the Human Protein Atlas[93] and coloured accordingly. Left dotted lines indicate the mean expression value of a gene across the whole dataset; right dotted lines indicate the 90th percentile value.

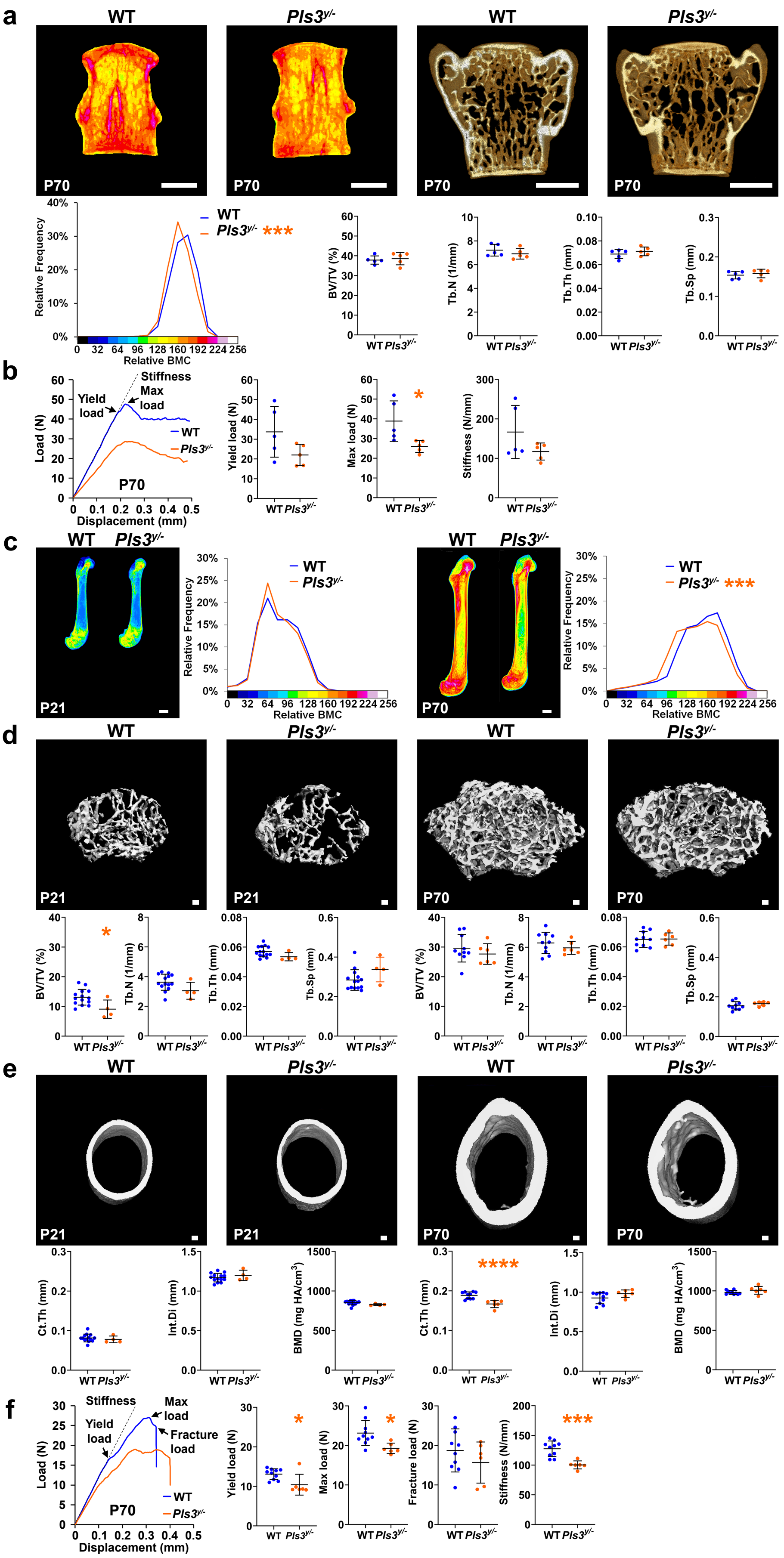
a
WT
Pls3y/-
P70
Relative Frequency
Relative BMC
BV/TV (%)
Tb.N (1/mm)
Tb.Th (mm)
Tb.Sp (mm)
b
Stiffness
Yield load
Max load
Load (N)
Displacement (mm)
Yield load (N)
Max load (N)
Stiffness (N/mm)
c
P21
d
e
Ct.Th (mm)
Int.Di (mm)
BMD (mg HA/cm3)
f
Fracture load
Fracture load (N)

**Supplementary Fig. 8. Skeletal phenotype of younger male *Pls3*$^{y/-}$ mice.**

(a) Pseudocoloured X-ray microradiography of P70 lumbar vertebrae (L5). Scale bars = 1mm. Relative frequency histogram shows BMC distribution (Kolmogorov-Smirnov test, ****P*<0.001). µCT images show mid-coronal sections. Scale bars = 1mm. Graphs show trabecular parameters (BV/TV, Tb.N, Tb.Th, Tb.Sp), analysed using mean±s.d (two-tailed Student's *t*-test). (b) Representative load displacement curves and graphs for P70 L5 compression testing (yield load, maximum load, stiffness), analysed using mean±s.d (two-tailed Student's *t*-test; **P*<0.05). (c) Pseudocoloured X-ray microradiography of P21 and P70 femurs. Low BMC is blue/green and high BMC is pink. Scale bars = 1mm. Relative frequency histograms show BMC distribution for each age comparison (Kolmogorov-Smirnov test, ****P*<0.001). (d) µCT images of distal femur trabecular bone from P21 and P70 mice. Scale bar = 100µm. Graphs show trabecular parameters (BV/TV, Tb.N, Tb.Th, Tb.Sp), analysed using mean±s.d (two-tailed Student's *t*-test; **P*<0.05). (e) µCT images of femur mid-shaft cortical bone from P21 and P70 mice. Scale bar = 100µm. Graphs show cortical parameters (Ct.Th, Int.Di, Ct.BMD), analysed using mean±s.d. (two-tailed Student's *t*-test; *****P*<0.0001). (f) Representative load displacement curves and graphs for P70 femur three-point bend testing (yield load, maximum load, fracture load, stiffness), analysed using mean±s.d (two-tailed Student's *t*-test; **P*<0.05, ****P*<0.001).

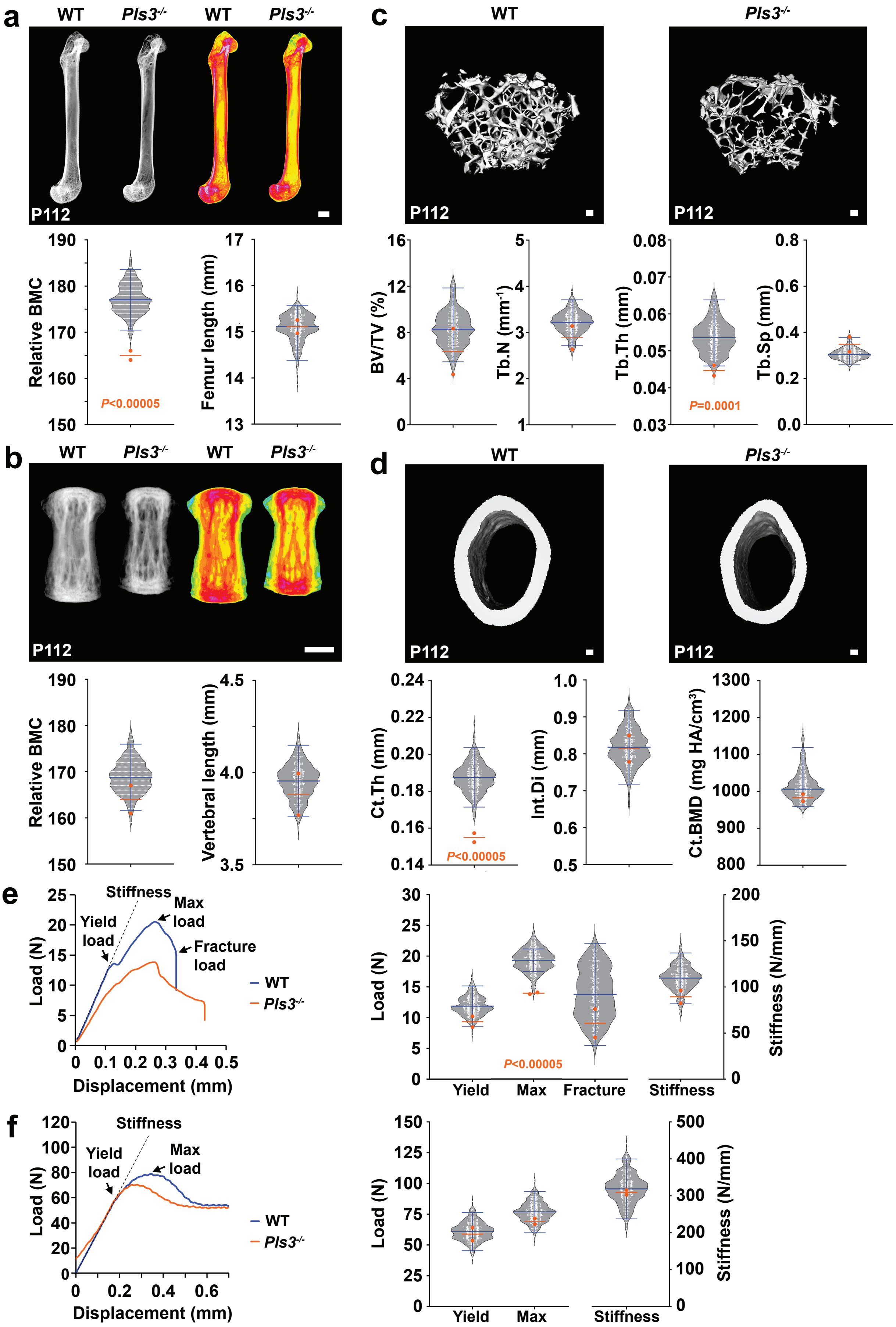

a
WT
Pls3-/-
WT
Pls3-/-
P112
Relative BMC
P<0.00005
Femur length (mm)
c
WT
Pls3-/-
P112
P112
BV/TV (%)
Tb.N (mm-1)
Tb.Th (mm)
P=0.0001
Tb.Sp (mm)
b
WT
Pls3-/-
WT
Pls3-/-
P112
Relative BMC
Vertebral length (mm)
d
WT
Pls3-/-
P112
P112
Ct.Th (mm)
P<0.00005
Int.Di (mm)
Ct.BMD (mg HA/cm3)
e
Stiffness
Max load
Yield load
Fracture load
Load (N)
Displacement (mm)
WT
Pls3-/-
Load (N)
Stiffness (N/mm)
P<0.00005
Yield
Max
Fracture
Stiffness
f
Stiffness
Yield load
Max load
Load (N)
Displacement (mm)
WT
Pls3-/-
Load (N)
Stiffness (N/mm)
Yield
Max
Stiffness

**Supplementary Fig. 9. Skeletal phenotype of female *Pls3*$^{-/-}$ mice.**

(a) Greyscale and pseudocoloured X-ray microradiography of P112 femurs from WT and *Pls3*$^{-/-}$ mice. Low BMC is blue/green and high BMC is pink. Scale bar = 1mm. Graphs quantify relative bone mineral content (BMC) and femur length. WT reference ranges are shown as grey violin plots (n=320), with individual *Pls3*$^{-/-}$ values (orange) overlaid. Significant *P* values after permutation testing are indicated. (b) Greyscale X-ray microradiography of P112 caudal vertebrae. Scale bar = 1mm. Graphs quantify BMC and vertebral length, displayed alongside the WT reference range violin plots. (c) µCT images of P112 distal femur trabecular bone. Scale bar = 100µm. Graphs quantify trabecular parameters: BV/TV, Tb.N, Tb.Th, and Tb.Sp. Significant P values after permutation testing are indicated. (d) µCT images of P112 femur mid-shaft cortical bone. Scale bar = 100µm. Graphs quantify cortical parameters: Ct.Th, Int.Di, and Ct.BMD. Significant *P* values after permutation testing are indicated. (e) Representative load displacement curves and graphs for P112 femur 3-point bend testing (yield load, maximum load, fracture load, stiffness). Significant *P* values after permutation testing are indicated. (f) Representative load displacement curves and graphs for P112 caudal vertebrae compression testing (yield load, maximum load, stiffness). All graphs in (b) to (f) indicate mean or median±s.d or percentiles.

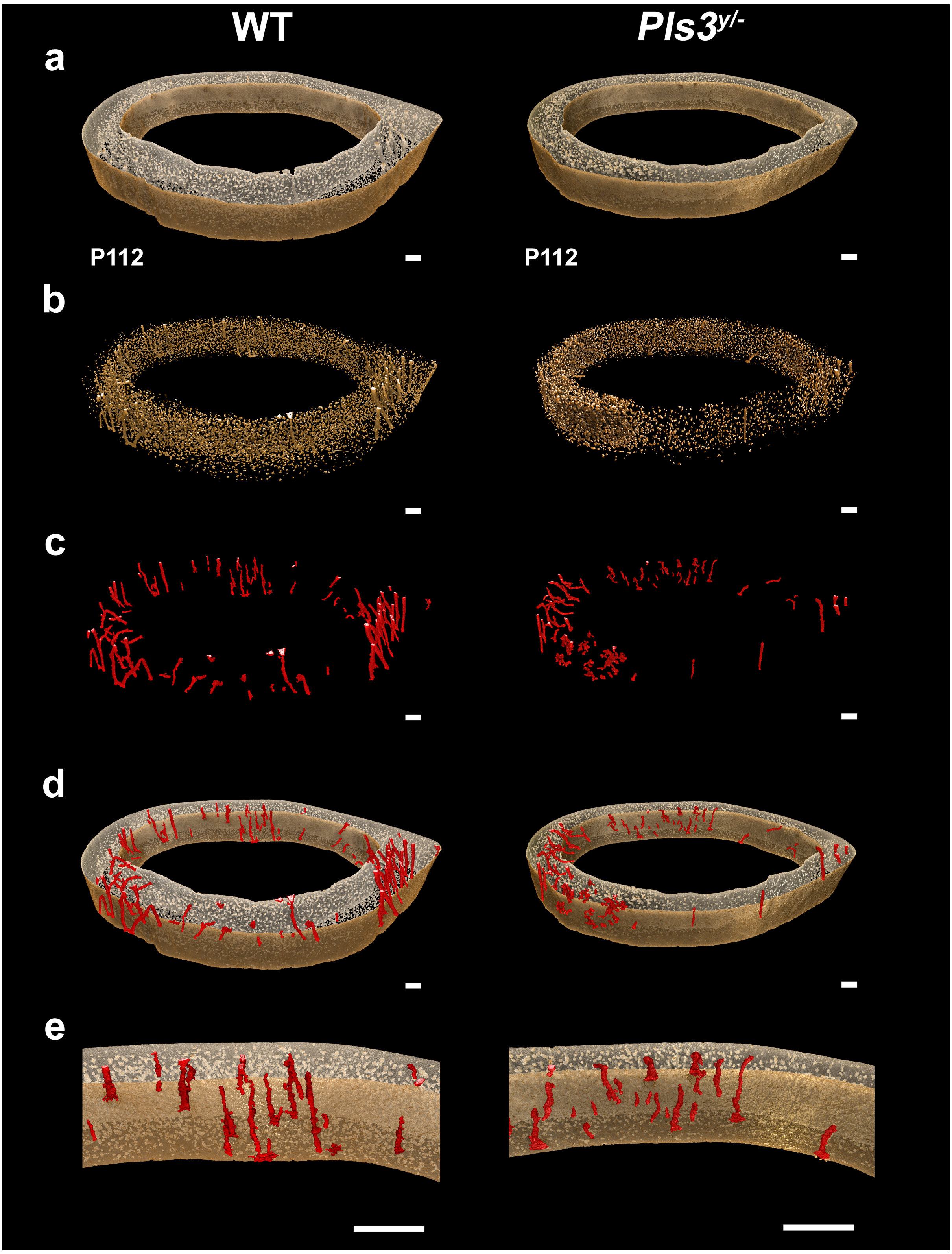


**Supplementary Fig. 10. Skeletal vascular phenotype of male *Pls3*$^{y/-}$ mice.**
(a) Micro-CT images (1.0 $\mu m^3$ voxel resolution) of mid-femur cortical bone (250 $\mu$m long ROI) from P112 (WT n=6; *Pls3*$^{y/-}$ n=6) mice. (b) Osteocyte lacunae and cortical vascular canals, within the cortical bone ROI, with a volume greater than 100$\mu m^3$ identified using BoneJ Particle Analyser. (c) Cortical vascular canals (Red), within the cortical bone ROI,

with a volume greater than 2000$\mu m^3$ identified using BoneJ Particle Analyser. (d) Overlay of mid-femur cortical bone ROI and vascular canals. (e) Higher power image of cortical bone and vascular canals. All scale bars = 100µm.

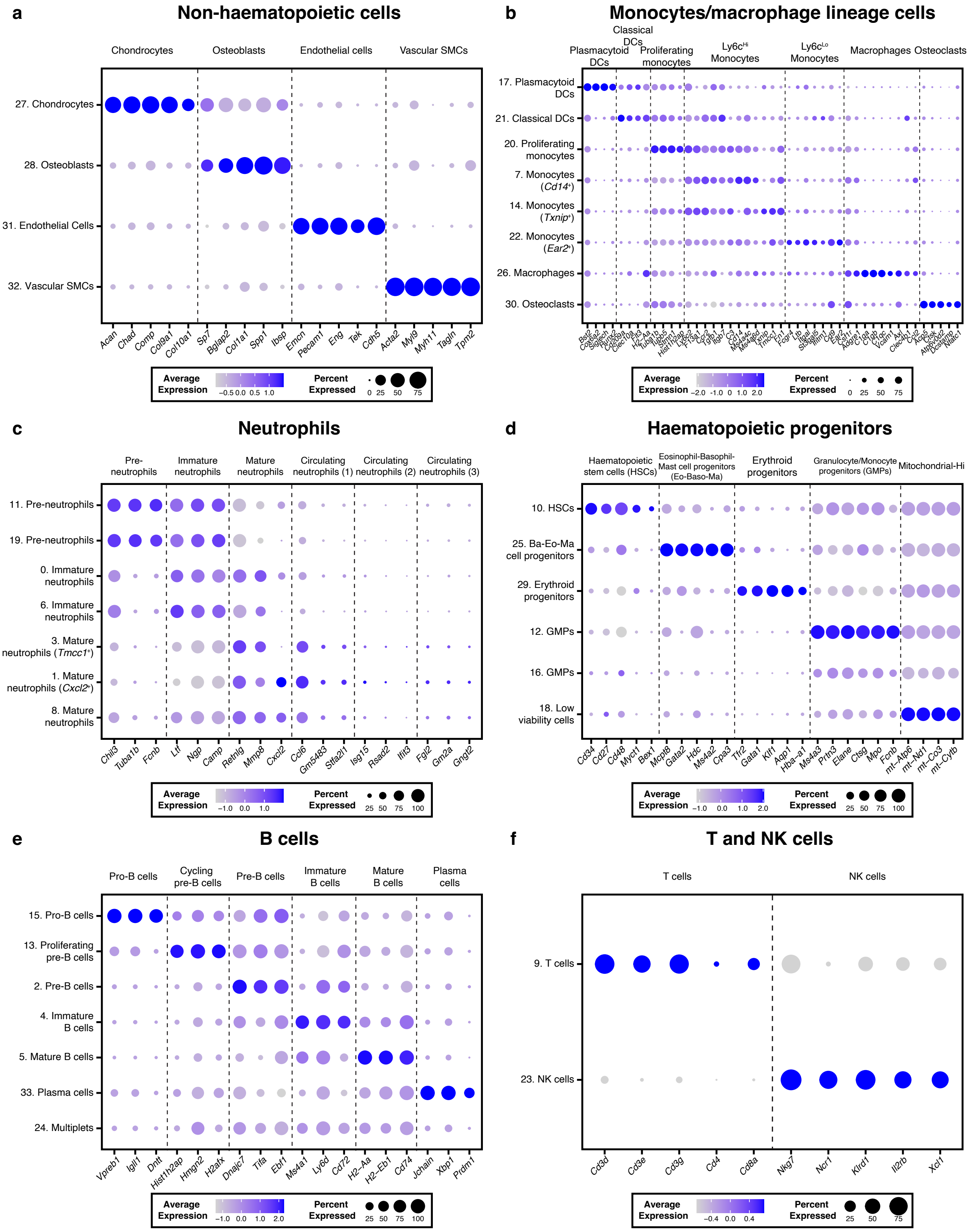


**Supplementary Note 1. Cell types were defined based on canonical marker genes and markers from published transcriptomics data.** Labels along y axes indicate identities of clusters within this dataset. Labels along X axes indicate reference cell types (either canonical cell types or specific clusters from published datasets) that are known to express the indicated genes. Annotation of: (a) Non-haematopoietic cells based on manually curated classical markers; (b) Cells of the monocyte and macrophage lineage based on microarray data from Menezes *et al*[1] and osteoclasts based on manually curated classical markers;

(c) Neutrophils using scRNA-seq data from Xie *et al*[2]; (d) Haematopoietic progenitor cells based on data from the Azimuth database[3]; (e) B cells using data from Lee *et al*[4]; (f) T cells and NK cells based on data from the Azimuth database[3]. Size of the circles represent the proportion of cells in each cluster expressing a gene. Scale bar indicates the average expression level of genes in a cell cluster.

**Supplementary Note 2. Detailed methods and materials**

**Mice for the isolation of bone cells and single-cell RNAseq (scRNA-seq)**

9–10-week-old male wildtype C57BL/6J mice were sourced from Australian BioResources and used in the isolation of cells from different bone compartments for scRNA-seq. Animal experiments were approved by the Garvan Institute of Medical Research Animal Ethics Committee (ARA16/01, ARA19/09 and ARA22/12).

**Isolation of cells from the endosteal and bone marrow compartments in mice**

To obtain endosteal compartment and bone marrow cells for scRNA-seq, mice were sacrificed via $CO_2$ asphyxia. Femurs were harvested from 5 groups of 5 mice. Soft tissue and epiphysis were removed from the femurs before being separated into diaphysis and metaphysis. Marrow cells were collected by flushing the diaphysis with PBS. Marrow-depleted diaphyseal and metaphyseal bone were crushed and cut up gently, then cells that are adherent to the endosteal surface were removed by digestion using 2mg/ml of collagenase A (Merck, 10103586001) and 2.5mg/ml of trypsin (Merck, T1426) for 30 mins at 37°C. After digestion, bone fragments were vortexed for 10s and the supernatant containing digested endosteal cells was filtered through a 100µm filter into collection tubes containing 10% fetal calf serum (FCS; Bovogen Biologicals, SFBS-AU). Marrow cells and endosteal cells were collected by centrifugation at 400x g for 5 mins and resuspended in 200µl PBS supplemented with 2% FCS prior to staining for FACS sorting.

**Isolation of cells from human femoral head bone**

Femoral head bone samples were obtained from patients with osteoarthritis undergoing total hip arthroplasty with approval from the St. Vincent's Hospital Sydney Human Research Ethics Committee (2022/ETH00475). Data for the patients and samples are provided in Supplementary Table 11. Femoral head samples were collected in RPMI medium and stored at 4°C for up to 18 hours prior to cell isolation. Bone cores were isolated from the femoral head using an electric core drill. Bone fragments from the femoral neck regions were obtained by cutting using secateurs. Bone cores and fragments were cut up into smaller bone chips using surgical blades and scissors. Cells were isolated from the bone chips using 2mg/ml of collagenase A (Merck) or collagenase IV (Worthington Biochemical, LS004186), and 2.5mg/ml of dispase (Merck, D4693) for 30 mins at 37°C. After digestion, bone chips were vortexed for 10s and the supernatant containing digested cells was filtered

through a 100μm filter into collection tubes containing 10% fetal calf serum (FCS; Bovogen Biologicals).

**FACS enrichment of mouse and human cells**

Mouse cells were stained for Ter119-PE (Biolegend, 116208) at 4ºC for 30 mins and rinsed with PBS supplemented with 2% FCS. Dead cells and debris were excluded by FSC, SSC and DAPI (ThermoFisher Scientific, D1306). Cells that were viable (DAPI-negative) and negative for erythroid marker (Ter119) were sorted into PBS supplemented with 2% FCS. The gating strategy is shown in Supplementary Note 3.

Human cells were stained for CD235-BUV395 (BD Biosciences, 563810) and CD45-APC-H7 (BD Biosciences, 641408) at 4ºC for 30 mins and rinsed with PBS supplemented with 2% FCS. Dead cells and debris were excluded by FSC, SSC and DAPI (ThermoFisher Scientific). Cells that were viable (DAPI-negative) and negative for erythroid marker (CD235a) were then sorted based on CD45 for haematopoietic (CD45-positive) and non-haematopoietic (CD45-negative) cells into PBS supplemented with 2% FCS. Gating strategy is shown in Supplementary Note 3.

**scRNA-seq**

Single cells were encapsulated into emulsion droplets using the 10x Chromium platform (10x Genomics). scRNA-seq libraries were constructed using the Chromium Single Cell 3' v2 Reagent Kit according to the manufacturer's protocol. Briefly, FACS sorted cells were examined under a microscope and counted with a cell counter (Thermo Fisher Scientific). Cells were loaded into each channel with a target output of 10,000 cells. Reverse transcription and library preparation were performed on a C1000 Touch Thermal cycler with 96-Deep Well Reaction Module (Bio-Rad). Amplified cDNA and final libraries were evaluated on an Agilent Tapestation using a High Sensitivity D1000 ScreenTape (Agilent Technologies, 5067-5584). Individual libraries were diluted to 4nM and pooled for sequencing. Pools were sequenced with 75 cycle run kits (26bp Read1, 8bp Index1 and 55bp Read2) on the Novaseq Sequencing System (Illumina) to 80-90% saturation level. scRNA-seq services were provided by the Garvan Genomics Platform at the Garvan Institute of Medical Research.

**Pre-processing of 10x scRNA-seq data**

Raw sequencing data were processed using the CellRanger pipeline (versions 2-7, 10x Genomics). Count matrices were loaded into R (version 4.5.1) and further processed using Seurat (versions 2-5)[3]. For scRNA-seq data we removed all cells with fewer than 300 distinct genes or cells with more than 10% unique molecular identifiers stemming from mitochondrial genes. Erythroid cells and plasma cells were excluded from the human scRNA-seq analysis to improve resolution of rarer populations and to mitigate donor-specific batch effects potentially associated with incidental monoclonal gammopathy.

Dimensionality reduction, clustering and sub-clustering of 10x scRNA-seq data

Dimensionality reduction was performed using gene expression data for the top 3000 variable genes. The variable genes were selected based on dispersion of binned variance to mean expression ratios using the FindVariableGenes function of the Seurat package. Next, principal component analysis (PCA) was conducted, and the first 40 principal components were included for the mouse scRNA-seq data, and the first 20 principal components were included for the human scRNA-seq data, for subsequent clustering and UMAP analysis based on manual inspection of a principal component variance plot ('PC elbow plot'). Human scRNA-seq data was batch corrected based on donor using Harmony[5]. Graph-based clustering of the PCA reduced data with the Louvain Method was performed after computing a shared nearest neighbour graph. The clusters were visualized on a 2D map produced with Uniform Manifold Approximation and Projection (UMAP). Multiple resolutions of clustering were conducted and the selected resolution represents clusters that best approximate the cell types of interest based on well characterised markers (Supplementary Note 1). For high resolution sub-clustering of the non-haematopoietic cells, the same procedure of finding variable genes, dimensionality reduction, and clustering was applied to the restricted set of data.

**Cell type annotation**

Cell types were identified using the Seurat clustering algorithm as described above, using both discrete and continuous variations in gene expression across cell clusters. Cell types were annotated based on canonical markers and markers identified in published bulk RNAseq and scRNA-seq datasets (Supplementary Note 1)[1-4]. For cell clusters without previous annotations from the literature, we used cluster-specific gene programs detailed below to annotate cells at the resolution of Leiden clusters.

Defining gene programs of each cell type using differential gene expression analysis

Differentially expressed genes for each cluster were identified using the FindAllMarkers function of Seurat and ROC-based test statistics for differential expression between every cluster versus all other clusters within the dataset. Genes with 1 or more UMI in at least 25% of cells within the two populations in the comparison (cluster-of-interest and all other clusters) were included in the analysis. Gene programs for each cluster included differentially expressed genes with $\log_2$fold-change [$\log_2$(FC)] of > 0.5 with a Bonferroni-adjusted *P* value of < 0.05. Gene programs can be found in Supplementary Table 2.

**Flow cytometry validation of enriched populations within the endosteal compartment**

Flow cytometric analysis was used to investigate the enrichment of cell populations within the endosteal compartment relative to the bone marrow. 9-week-old male C57BL/6J mice were sacrificed via $CO_2$ asphyxiation and both femora and tibiae were collected. Soft tissue and the epiphyses were removed and marrow collected by flushing with PBS. Marrow-depleted bones were then crushed gently. To liberate adherent cells, marrow cells and bone fragments were subjected to enzymatic digestion with 2mg/ml collagenase A, 2mg/ml dispase and 0.1mg/ml DNaseI for 30 mins at 37ºC. Following digestion, cells were filtered through a 100μm filter into collection tubes containing FCS (Bovogen Biologicals). Cells were collected via centrifugation at 400x g for 5 mins. Red blood cell lysis was then performed (Roche Diagnostics) and cells again collected by centrifugation. Cells were counted and incubated with Fc block (Biolegend; 101302) for 5 mins on ice. Cells were then stained with Zombie NIR Viability Stain (Biolegend; 423106), B220-BV510 (Biolegend; 103248), TCRβ-BV510 (Biolegend; 118131), CD45-BV650 (Biolegend; 103151), CD11b-BUV395 (BD Bioscience; 565976), Ly6C-BUV737 (BD Bioscience; 755201), Ly6G-APC (BD Bioscience; 560599) and CD14-PE (BD Bioscience; 569968) for a further 25 mins on ice. Cells were washed in PBS supplemented with 2% FCS. Samples were acquired using a BD FACSymphony machine and analysed with FlowJo v10.10.0 (BD Life Sciences).

**Defining genes with cell type-restricted expression**

The restrictedness of gene expression was calculated by determining the number of clusters expressing a given gene. A cluster was deemed to express a gene if 1 or more UMI for that gene was detected in at least 25% cells within the cluster.

**Cell-cell interaction analysis**

Putative intercellular interactions between clusters were identified via ligand-receptor analyses using CellPhoneDB v5.0.1[6]. Following Seurat clustering analysis, gene symbols were converted to HGNC symbols using the biomaRt package (version 2.64.0)[7]. CellPhoneDB analysis was then performed using the "statistical_analysis_method" with default settings. A complete table of results is available in Supplementary Table 4. Within this process, all interactions are ascribed a directionality indicating which cell type is the "sender" cell and which cell type is the "receiver cell".

To identify meaningful signalling pathways and processes associated with the identified ligands and receptors, we interrogated the ReactomeDB database of biological processes [8] for enriched terms using the "enrichPathway" function in the ReactomePA package (version 1.52.0)[9]. All ligands and receptors involved in interactions between a given pair of cell types were used as input, with all genes present in the sequencing dataset used as the background universe. Enriched terms were annotated according to their root term within the ReactomeDB database and the level that they appear within that classification (root term = level 1). To minimise overlapping and redundant terms, Jaccard similarity values were calculated between each pair of terms based on the genes contributing to each term. Terms with a Jaccard similarity score of 0.8 and above were considered redundant and collapsed into a single term, with the term at level 3 selected for visualisation. Data were visualised using the ggplot2 (version 4.0.0) and viridis (version 0.6.5) R packages[10].

**Reconstruction of cell differentiation trajectories**

To infer the progression of cells across multiple differentiation stages and order them in pseudo-time, the algorithms implemented in the Monocle package (version 2) were used[11]. The top 1000 significantly differentially expressed genes were selected as the ordering genes for the trajectory reconstruction, using the nonlinear reconstruction algorithm DDRTree.

**Comparison between mouse and human scRNA-seq datasets**

To compare scRNA-seq datasets we performed label transfer analysis with Seurat (version 5.3.0)[12], using the annotated mouse scRNA-seq dataset as the reference and the human scRNA-seq dataset as the query. A shared set of 3000 variable genes was identified using SelectIntegrationFeatures across both datasets. Transfer anchors were computed using FindTransferAnchors with the first 20 principal components. Cell-type annotations from the

mouse reference were then transferred to the query datasets using TransferData, and prediction scores were added to the metadata. The prediction scores are derived from a softmax-like normalisation of label transfer anchors and reflect the maximum probability assigned to a transferred label for a given query cell. A prediction score of **>** 0.5 identifies high-confidence cell type predictions[12].

**Identification of transcription factors regulating gene programs in cell clusters**

To identify the transcription factors and their downstream target genes (regulons), the package pySCENIC (version 0.11.2) was used[13]. Gene regulatory network inference was performed with GRNBoost2, co-expression modules and potential direct targets of transcription factors were identified and then filtered using cisTarget. Regulon activity on single cells was scored using AUCell. The analysis was performed according to the standard SCENIC workflow.

**Single-nucleus ATAC-seq (snATAC-seq)**

Endosteal cells were isolated from femora and tibiae of 9-week-old male *C57BL/6J* mice as described above. Single-cell suspensions were washed with PBS containing 1% BSA and pelleted (500 × g, 5 min, 4 °C). Cells were lysed in 100 $\mu$L custom lysis buffer (0.0075% digitonin, 0.05 mg/mL protease inhibitor (Millipore Sigma, 114298668001), 20 mM Tris-HCl [pH 7.4], 150 mM NaCl, 3 mM $MgCl_2$, 2% BSA) on ice for 1–2 min with gentle pipetting to facilitate lysis. Nuclei were washed with washing buffer (0.025 mg/mL protease inhibitor, 20 mM Tris-HCl [pH 7.4], 150 mM NaCl, 3 mM $MgCl_2$, 1% BSA) and pelleted (300 × g, 8 min, 4°C). Single-nucleus ATAC-seq (snATAC-seq) was performed using the 10x Genomics Chromium Single Cell ATAC v2 platform, following the manufacturer's protocol. Briefly, nuclei were counted using a LUNA counter and haemocytometer to assess concentration and nuclear integrity. Nuclei were diluted to the recommended loading concentration and transposed using the Tn5 transposase in the ATAC transposition mix. Following transposition, gel bead-in-emulsions (GEMs) were generated using the Chromium Controller, enabling barcoding of accessible chromatin fragments in individual nuclei. Post-GEM amplification and library construction were carried out according to the 10x Genomics protocol. Final libraries were quantified using Qubit and assessed for fragment distribution using the Agilent Bioanalyzer or Tapestation. Sequencing was performed on an Illumina NovaSeq 6000 with paired-end 50 bp reads, targeting a depth of ~25,000–50,000 fragments per nucleus. snATAC-seq services were provided by the Garvan Genomics Platform at the Garvan Institute of Medical Research.

Raw snATAC-seq sequencing data were processed using Cell Ranger ATAC (v2.1.0, 10x Genomics) for demultiplexing, alignment to the reference genome, barcode filtering, and peak calling. A median of 12,108 high-quality fragments per cell were detected, with 82.3% of fragments demonstrating high-quality overlap with peaks. The resulting fragment files were imported into R (v4.5.1) and analysed using the Signac (v1.15.0) and Seurat (v5.3.0) packages. Low-quality nuclei were filtered based on nucleosome signal, TSS enrichment score, and fragment count thresholds to remove likely debris or doublets. Peaks were quantified using the FeatureMatrix function, and term frequency–inverse document frequency (TF-IDF) normalization was applied, followed by dimensionality reduction using singular value decomposition (SVD) on the top components. Clustering was performed using the shared nearest neighbour (SNN) graph approach, and UMAP was used for two-dimensional visualization. Gene activity scores were computed by aggregating chromatin accessibility in gene bodies and promoter regions and integrated with matched scRNA-seq data using label transfer via canonical correlation analysis (CCA), when applicable. Differential accessibility analysis between clusters or conditions was performed using logistic regression implemented in FindMarkers with fragment counts as covariates. Enriched transcription factor binding motifs within differentially accessible peak regions were identified using the FindMotifs function with default settings.

**Identification of genes within the gene programs that affect the skeleton**

Genes from gene programs associated with biological processes important in the skeleton were identified using a curated list of gene ontology (GO) biological processes[14] directly related to the skeleton[15]. Briefly, this list was constructed by filtering GO term descriptions using bone-related keywords. Genes associated with any of the 116 manually curated skeletal biological processes were then identified. This resulted in a final list of 663 genes. This list may be found in Supplementary Table 3.

Similarly, genes that cause a significant skeletal phenotype when mutated in mice were identified from the Mouse Genome Informatics (MGI) database[16]. Mouse gene ids (mgi_ids) with “abnormal skeletal phenotypes” were extracted using the mouse phenotype identifier MP:0005390 (date of access: 22$^{nd}$ September 2023). Genes with alternative gene symbols or typographical errors were manually corrected and the list filtered for protein coding genes. This resulted in a final list of 2811 genes. This list may be found in Supplementary Table 3.

**Hypergeometric over-representation testing**

To determine whether gene programs or lists were enriched for a specific subset of genes, over-representation analyses were performed using Fisher's Exact Test under the hypergeometric distribution. Briefly, this test determines whether the number of genes of interest that are present within a given list of genes (successes in sample) is higher than would be expected by chance based on the total number of genes of interest within the background population (successes in population). For each test, a *P* value was calculated corresponding to the strength of evidence to reject the null hypothesis of no enrichment and adjusted for multiple testing via the Bonferroni correction method.

Parameters and details of *P* value adjustments for each over-representation test are outlined in Supplementary Table 12. For consistency, human Ensembl IDs were used as inputs for each test, using the biomaRt R package (version 2.64.0)[17] to convert from other gene identifiers where appropriate. All tests were performed using the RITAN package (version 1.24.0) in R[18]. Results are visualised with bar plots, bubble plots and FeaturePlots generated using the ggplot2 and Seurat R packages. Dark blue shades indicate significant enrichments ($P \leq 0.05$ after Bonferroni correction); light blue shades indicate nominally significant enrichments ($P \leq 0.05$ before Bonferroni correction and $P \geq 0.05$ after Bonferroni correction).

**Identifying gene programs enriched for monogenic skeletal disorder genes**

To investigate whether skeletal disorder-causing genes were enriched among gene programs of bone and marrow cells, the current International Skeletal Dysplasia Society (ISDS) Nosology and Classification of Genetic Skeletal Dysplasias database was used[19]. This database encompasses 771 rare monogenic skeletal disorders categorised into 41 disorder groups (based on shared clinical, radiographic and molecular phenotypes), with pathogenic variants in 552 protein-coding genes or chromosomal aberrations attributed as causative. Genes with alternative gene symbols or typographical errors were manually corrected. The list was filtered for protein coding genes and genes that mapped to corresponding mouse orthologs using the biomaRt package in R (version 2.64.0)[17]. This resulted in a final list of 528 genes which can be found in Supplementary Table 3.

Hypergeometric tests were performed and visualised as described above. Network plots were generated to allow for visual interpretation of enrichment analysis by depicting linkage between cells and the genes they expressed. Network plots were visualised using Cytoscape software (version 3.10.0)[20].

**Quality control of ultrasound derived bone mineral density in the UK-Biobank Study**

Between 2006 and 2010, the UK Biobank Study recruited 502,647 individuals aged between 37 and 76 years (99.5% were 40-69 years) located across the UK. The Northwest Multi-Centre Research Ethics Committee approved the UK Biobank Study, and informed consent was obtained from all participants. Quantitative ultrasound (QUS) assessment of calcanei was conducted on UK Biobank Study participants using a Sahara Clinical Bone Sonometer [Hologic Corporation (Bedford, Massachusetts, USA)]. Details of the complete QUS protocol is publicly available on the UK Biobank data showcase. Estimated heel bone mineral density [eBMD, (g/cm2)] was derived as a linear combination of two QUS parameters: speed of sound [SOS, (meters/second)] and bone ultrasound attenuation [BUA, (decibels/megahertz)] using methods described in Morris *et al*[21]. A total of 481,380 valid measures for eBMD passed QC (264,757 females and 216,623 males).

**Identifying study participants with European ancestry.**

UK Biobank Study participants with high quality genotyping data (N=486,445) were projected onto the first 20 ancestry informative principal components (PCs) estimated from 1000 Genomes Project Phase 3 (1KG) individuals using GCTA version 1.93.2. PCA was based on a curated set of 38,512 LD-pruned HapMap3 (HM3) [20] bi-allelic SNPs that were shared between the 1KG and UK Biobank genotyped datasets [i.e. Minor allele frequency (MAF) > 1%, minor allele count (MAC) > 5, genotyping call rate > 95%, Hardy-Weinberg $P > 1\times10^{-6}$, and 13 regions with excessive linkage disequilibrium excluded]. The first 20 ancestry informative principal components of UK Biobank and 1KG individuals were projected into 3 components using Uniform Manifold Approximation and Projection (UMAP) using the following parameters: min_dist=0.000001, n_components=3, n_neighbors=35, random_state=10293082. Parameters were defined through supervised clustering by iterating over different parameter combinations and selecting parameters that clustered 1KG individuals with the same ancestry. UK Biobank Study participants that clustered with 1KG European ancestry individuals were identified by visual inspection. These individuals were then mapped back to PCA plots to ensure they co-localised with 1KG European ancestry individuals. Based on this analysis a total of 461,920 UK Biobank Study participants were deemed to have European ancestry.

**Genome-wide association analysis**

A maximum of 448,010 European individuals (245,157 females and 202,853 males) with genotype and valid eBMD measures were available for analysis. As compared with a recent UK Biobank eBMD GWAS conducted by Morris and colleagues[21], our study included 21,186 more individuals for the analysis of autosomal variants, and 85,085 more individuals for analysis of the sex chromosomes. The larger sample size was attributable to including individuals with European ancestry, as opposed to British ancestry analysed by Morris. Our study also included related individuals in the analysis of the X-chromosome, where Morris only analysed unrelated individuals due to software limitations. We also analysed the 3rd version of UK Biobank imputed genetic data that had ~10M more autosomal genetic variants and ~730k more X chromosome variants than version 2 analysed by Morris.

Autosomal genetic variants and variants on the X and pseudoautosomal regions were tested for associations with eBMD assuming an additive allelic effect. A linear mixed non-infinitesimal model implemented in BOLT-LMM v2.3.4 was used to account for population structure and cryptic relatedness. Prior to GWAS eBMD had been transformed using a ranked based inverse normal transformation (mean=0, sd=1) and the following covariates were included as fixed effects in all models: age, sex, genotyping array, and ancestry informative principal components 1 to 20. Analysis was restricted to 20,910,639 high-quality imputed variants on the autosomal chromosomes, and 774,996 high-quality imputed variants on X-chromosome with a MAF > 0.05%, and imputation info score > 0.3.A Manhattan plot summarising genome-wide associations was generated using ggplot2 library within R[10].

The genomic inflation factor ($\lambda_{GC}$) was used to quantify the false positive rate and extent to which GWAS test statistics were inflated due to latent confounding. $\lambda_{GC}$ was calculated as a ratio of the median of the empirically observed distribution of $chi^2$ test statistics to the expected median (i.e., 0.456). The contribution of population stratification and other potential latent sources of bias to genomic inflation was quantified using stratified linkage disequilibrium score regression (LDSR) implemented in LDSC (version v1.0.1) in conjunction with pre-computed LD-Scores from European individuals from the 1000 Genomes project, assuming the BaselineLD model. LDSR analysis was limited to HapMap3 variants that passed QC using default software settings, supplemented with the --chisq-max 5000 flag to prevent disproportionate numbers of SNPs with small *P* values being excluded because of the software not being able to interpret double-precision floating-point number formats. The LDSC attenuation ratio statistic (RPS) was modest [$LDSC_{RPS}$=0.02 (0.0195)], suggesting that

polygenicity was the main driver of the observed inflation of test statistic (i.e. Genomic inflation factor $\lambda_{GC} = 2.27$). The high SNP heritability of eBMD ($h2_{SNP}$=0.39 se=0.027) and large sample size of the study resulted in the LDSC intercept exceeding 1 ($LDSR_I$=1.08 se = 0.073).

SNPTracker (version 1.0) was used to update reference SNP cluster IDs (rsID) and base pair positions from Hg19 to GRCh38h using dbSNP release 151[22]. This resulted in a final dataset containing 21,628,442 variants. SnpEff 5.1d[23] was used in conjunction with GRCh38.p14 databases to assess the putative impact of all genetic variants and classify them as either high, moderate, or low impact. High impact variants are assumed to have high (disruptive) impact in the protein, causing protein truncation, loss of function or triggering nonsense mediated decay. Moderate impact variants are non-disruptive, but likely to change protein effectiveness. Low impact variants are assumed to be mostly harmless or unlikely to change protein behaviour. All high impact variants were cross referenced using Variant Effect Predictor (VEP) to confirm that SNP alleles and predictions were consistent[24].

**Fine mapping associated loci**

Statistically independent genetic variants robustly associated with eBMD were identified using approximate conditional and joint association analysis (COJO)[25] implemented in GCTA (version 1.94.1)[26]. The genome-wide significance threshold derived previously by Kemp and colleagues was applied ($P < 6.6\times10^{-9}$)[27]. Genetic variants with high collinearity (multiple regression $R^2 > 0.8$) were ignored and those situated more than 10 mega bases (MB) away were assumed to be in complete linkage equilibrium. To model patterns of linkage disequilibrium (LD) between genetic variants in GWAS, a reference sample of ~50,000 unrelated European ancestry individuals from the UK Biobank Study was used[26,28]. The LD reference sample of unrelated individuals was identified using KING (kinship coefficient <0.044)[29] implemented in PLINK (version 1.9)[30]. The following variant level QC was applied to the resulting dataset, and variants were excluded if they deviated from Hardy Weinberg Proportions ($P < 1\times10^{-6}$), had a missing call rate less than 95%, were rare [(MAF) < 0.01%, and had a minor allele count (MAC) > 1]. The proportion of phenotypic variance explained by statistically independent association signals was calculated by summing the variance explained by primary and secondary signals using the formula described in Morris *et al*[21]:

$$2p(1 - p)\beta j^2$$

where p is the effect allele frequency, and $\beta j$ is the joint and conditional effect of the allele on a standardized phenotype (mean = 0, variance = 1).

**Annotation of independent association signals**

Statistically independent variants reaching genome-wide significance were binned to 'loci' using a 1 MB sliding window. Loci were deemed "novel" if they were located > 1 MB from any SNP reported to be associated with "bone density" in the NHGRI-EBI GWAS Catalog. For this annotation a reference list of BMD-associated SNP RSIDs meeting genome-wide significance ($P < 5\times10^{-8}$) was downloaded from the GWAS Catalog on 05/03/2024 using the term "EFO_000392". SNPs identified by studies using interaction models and/or multi-trait analyses were removed. The corresponding Hg38 co-ordinates of remaining SNPs were obtained using SnpTracker as previously described, and bedtools (version 2.29.2)[31] was used to estimate the minimum distances between each lead variant and the list of BMD associated variants. Lead association signals were also annotated to the closest protein coding gene using bedtools in conjunction with the Ensemble Genes 105 dataset that was downloaded from BioMart and based on genome-build GRCh38.p14[17].

The closest protein coding gene to each lead variant were then followed up in the ISDS 2023 Nosology and Classification of Genetic Skeletal Diseases and further annotated if they caused monogenic skeletal disorders in humans when mutated[19].

**MAGMA gene-based tests of association**

Gene-based tests of association were conducted using MAGMA software (version 1.10) in conjunction with GWAS summary statistics of variants with imputation quality score > 0.6 and MAF > 0.05%. Patterns of linkage disequilibrium (LD) were modelled with a reference sample of ~50,000 unrelated European ancestry individuals from the UK Biobank Study. Genetic variants were annotated to protein coding genes based on Ensembl gene version 105 GRCh38. Variants were annotated to a gene if they were mapped ≤ 2kb upstream of the transcriptional start site and ≤ 1kb downstream of the stop site. A multi-model approach that combined the summary association results from 2 gene analysis models was used to derive an aggregate P value ($P_{\text{Multi}}$) corresponding to the overall strength of evidence of association between each protein coding gene and eBMD. This multi-model approach was chosen as it yielded a more even distribution of statistical power and sensitivity over a wider range of different genetic architectures. The two gene-analysis models included in the multi-model were the 'SNP-wise mean' model that calculates the mean of $\chi2$ statistics for all genetic variants annotated to a gene, as well as the 'SNP-wise top' model that uses the calculates the $\chi2$ statistic for the lead genetic variant annotated to a gene. The threshold to declare

statistical significance was determined using Bonferroni correction as follows: error rate of one test / number of genes tested = 0.05 / 19,695 = $P < 2.54\times10^{-6}$.

**Enrichment analysis involving eBMD associated genes**

Hypergeometric tests were performed and visualised as described above to investigate whether rare skeletal disorder causing genes were over-represented among the set of protein coding genes located closest to independent association signals, and separately the set of genes associated with eBMD as defined by MAGMA gene-based tests of association (Supplementary Table 7). Further hypergeometric tests were then used to determine whether enrichment was attributable to genes that caused individual skeletal dysplasia groups. The ISDS Nosology database was processed as outlined above with one alteration: the list was not filtered for genes with mouse orthologs. This resulted in a final list of 533 genes.

**Identification of gene programs enriched for eBMD-associated genes**

Competitive gene set analysis (GSA) was used to determine whether the gene-programs of different bone and marrow cells isolated from mice were enriched for eBMD associated genes. This was achieved by testing whether the set of genes making up the gene-program of a cell type was on average more strongly associated with eBMD, as compared with all other protein coding genes that were not in the gene-program under investigation. GSA accounted for several confounding factors including gene size, gene density and the inverse of the mean minor allele count in the gene, as well the log value of these three factors. The threshold to declare statistical significance was determined using Bonferroni correction as follows: Error rate of a single test / number of gene programs tested = 0.05 / 34 = $P < 1.5\times10^{-3}$. GSA was also conducted on gene programs of the 16 non-haematopoietic cell sub-clusters. A *Bonferroni*-corrected significance threshold of 0.05 / 16 = $P < 3.1\times10^{-3}$ was used to identify enriched cell sub-types.

Post-hoc permutation analysis was conducted using R-scripts supplied with the MAGMA software. Exhaustive pairwise conditional analysis was also conducted on all enriched cell types to account for genes that were shared between gene programs of enriched cell types and cell-sub-clusters. A conditional *P* value < 0.05 was used to reject the null-hypothesis no-enrichment after correcting for shared genes.

GSA was also used to determine whether the gene-programs of different bone and marrow cells isolated from humans were enriched for eBMD associated genes. See Supplementary Table 7 for more information regarding significance thresholds and correcting for multiple testing.

**Pulse rate as a negative control to for our gene enrichment workflow**

We hypothesised that the cellular and genetic determinants of pulse rate were unlikely to be shared with those that regulate BMD. After conducting the analysis of eBMD, pulse rate was selected as a negative control for our study. We analysed pulse rate in the same set of individuals from the UK Biobank Study using the same workflow described above and compared results of enrichment analyses.

**Identification of gene programs enriched for genes that cause abnormal bone structure when mutated in mice**

Hypergeometric tests were performed and visualised as described above to investigate whether genes that regulate bone structural integrity were over-represented among gene programs of cell types and non-haematopoietic sub-clusters that were enriched in previous analyses (Supplementary Table 3). Genes that cause a significant bone structural phenotype when mutated in mice were identified from the Mouse Genome Informatics (MGI) database[16]. Mouse gene ids (mgi_ids) with “abnormal bone structure” phenotypes were extracted using the mouse phenotype identifier MP:0003795 (date of access: 21st May 2024). Genes with alternative gene symbols or typographical errors were manually corrected. The list was filtered for protein coding genes and genes that mapped to corresponding human orthologs using the biomaRt package in R[17]. This resulted in a final list of 1347 genes. This list may be found in Supplementary Table 3.

To determine whether genes that were more highly expressed in each gene program were more strongly enriched for genes that regulate bone structural integrity, we allocated genes to 6 nested groups based on the magnitude of fold-change in gene expression and repeated the gene-set enrichment analysis using all genes from each group, and then using only eBMD- associated genes from each group. The same methodology was applied to determine if gene programs of non-haematopoietic cell sub-clusters were enriched for genes that regulate bone structural integrity.

**Non-osseous tissue gene expression analysis**

To interrogate expression of genes across tissues in mice, the TabulaMuris database was used[32]. Processed scRNA-seq data was downloaded for each of the SMART-seq FACS-based datasets from FigShare (https://figshare.com/articles/dataset/Single-cell_RNA-seq_data_from_Smart-seq2_sequencing_of_FACS_sorted_cells/5715040).

To interrogate expression of genes across tissues in human, the TabulaSapiens database was used[33]. Processed scRNA-seq data were downloaded from FigShare (https://figshare.com/articles/dataset/Tabula_Sapiens_v2/27921984) and converted to Seurat objects using the "convertFormat" function from the sceasy package in R (version 0.0.7)[34]. Datasets were filtered to include only cells analysed via SMART-seq.

Each tissue was analysed individually. Cell clusters with <10 cells were excluded from analysis. Remaining clusters were manually assigned to a cell group (e.g. epithelial, mesenchymal, immune etc) using the Human Protein Atlas classification system (https://www.proteinatlas.org/humanproteome/single+cell+type)[35]. The mean raw expression of selected exemplar genes within each cluster was then calculated and visualised as dotplots using the ggplot2 R package (version 4.0.0)[36]. Mean expression values and cluster cell group assignments can be found in Supplementary Table 10.

**Genetically modified mice**

Animal experiments were performed and reported in accordance with ARRIVE guidelines[37]. All mouse skeletal phenotyping studies were undertaken under licence at Imperial College (project licence PPL70/8785 and PP1540664) and the Wellcome Trust Sanger Institute Mouse Genetics Project as part of the International Mouse Phenotyping Consortium and licenced by the UK Home Office (PPLs 80/2485 and P77453634) in accordance with the 1986 Animals (Scientific Procedures) Act and the recommendations of the Weatherall report. Animal experiments were approved by the Sanger or Imperial College Hammersmith Campus Animal Welfare Ethical Review Bodies (AWERB) as appropriate.

***Pls3* knockout mouse line**

C57BL/6N mice carrying a *Pls3*$^{tm1a(EUCOMM)Wtsi}$ knockout first allele (MGI:104807) were obtained from the Wellcome Trust Sanger Institute EMMA mouse repository and re-derived from frozen embryos. Genotyping was performed according to supplier's protocols. The *Tm1a* knockout allele contains an internal ribosome entry site (IRES)-lacZ cassette in intron

3. The skeletal phenotype was studied in hemizygous male (*Pls3*$^{y/-}$) mice and compared to WT (*Pls3*$^{y/+}$) male littermates. Following sacrifice, skeletal tissues were placed immediately into 70% ethanol or fixed in 10% neutral buffered formalin for 24 hours prior to storage at 4°C in 70% ethanol until analysis. All studies were approved by the Imperial College London AWERB and performed in accordance with the UK Animal (Scientific Procedures) Act 1986, the ARRIVE guidelines and EU Directive 2010/63/EU. All analyses were performed blind to sample genotype identification.

**Wild Type and mutant mice generated by the International Mouse Phenotyping Consortium**

Samples from 16-week old wild-type (WT) and genetically-modified mice designed with deletion alleles on the C57BL/6Brd-*Tyr*$^{c\text{-}Brd}$, C57BL/6Dnk, and C57BL/6N backgrounds were generated as part of the Wellcome Trust Sanger Institute's (WTSI) Mouse Genetics Project (MGP), part of the International Mouse Phenotyping Consortium (IMPC; http://www.mousephenotype.org). Details on back-crossing status, weight, health status, administered drug and procedures, husbandry and specific conditions (including housing, food, temperature and cage conditions) have been reported previously[38,39]. All mice generated by the WTSI MGP underwent a broad primary phenotype screen using consortium-wide protocols available at the IMPC portal (www.mousephenotype.org/impress). This includes body length, x-ray skeletal survey and biochemical measures of mineral metabolism performed between 14-16 weeks of age.

**Origins of Bone and Cartilage Disease bone phenotyping pipeline**

Female mice from all WTSI MGP lines included in this study were sacrificed at 16 weeks of age, with the lower limb and tail fixed in 70% ethanol and subsequently phenotyped by the Origins of Bone and Cartilage Disease programme (OBCD) at Imperial College London. All samples were anonymised and randomly assigned to batches of 75 samples for rapid throughput analysis in a blinded and unselected fashion. The mouse line and genotype of each sample was only unblinded once all analyses had been completed for a batch. At least 5 contemporaneous WT samples were included in each batch to allow the identification of any systematic batch errors. The OBCD skeletal phenotyping programme determined 19 parameters of bone mass and strength[21,27,40]. The key phenotype definitions are as follow:

• Structural phenotypes – Significant difference in bone structural parameters measured by digital X-ray microradiography or micro-computerised tomography.

•	Functional phenotypes – Significant differences in mechanical strength parameters measured by biomechanical testing
•	Structural and functional phenotypes – Significant differences in at least one structural and one functional parameter
•	Bone quality phenotypes – lines with outlier functional phenotypes that did not correlate with bone mineral content
•	Mahalanobis phenotypes – lines that were significant outliers due to smaller differences in multiple skeletal parameters.

**Digital x-ray microradiography**

Soft tissue was removed from the fixed bones and digital X-ray images recorded at a 10µm resolution using a Faxitron MX20 variable kV point projection x-ray source and digital imaging system (Qados, Cross Technologies plc, Sandhurst, Berkshire, UK) operating at 26kV, 15s, and 5x magnification. The magnification was calibrated by imaging a digital micrometer. For each sample the cleaned lower limb and caudal vertebrae Ca6 and Ca7 were imaged in frame with 3 standards; a 1mm diameter steel wire, a 1mm diameter spectrographically pure aluminium wire, and a 1mm diameter polyester fibre. Relative bone mineral content, a two-dimensional parameter similar to areal bone mineral density, and bone lengths were determined as previously described[41]. Briefly, 2368x2340 16-bit DICOM images were converted to 8-bit Tiff images in ImageJ, the grey levels for the polyester and steel standards were used to stretch each image across the 256 grey levels with polyester at grey level 0 and steel at grey level 255 using macros described in[42]. Increasing gradations of mineralisation density were represented in 16 equal intervals by applying a pseudocolour lookup table to each image. For each sample the median grey level (0-255) of the femur and caudal vertebrae 6 and 7 was calculated by determining the number of pixels of each grey level in the stretched X-ray image. Lengths were determined using ImageJ 1.44 software. The results for caudal vertebrae 6 and 7 were averaged. For male *Pls3*$^{y/-}$ mice, cleaned lower limb, caudal vertebrae Ca6 and Ca7, and lumbar vertebrae L5 were imaged at 10*µ*m pixel resolution using a Faxitron UltraFocus digital radiography system (Faxitron Bioptics LLC, Arizona, USA).

**Micro-computerised tomography (*µ*CT)**

A Scanco *µ*CT50 (Scanco medical, Zurich, Switzerland) was used to determine the three-dimensional cortical and trabecular structural parameters of femurs. Samples were scanned at 70kV, 200µA, with a 0.5mm aluminium filter, 1 second integration time, no averaging, and

images captured every 0.36◦ though 180◦ rotation. The Scanco Medical software suite (μCT Tomography v6.4-2/Open VMS) was used for reconstructions, ROI selection and analyses. 10μm voxel resolution scans of a 1.5 mm long region of mid-shaft cortical bone were used to calculate cortical bone parameters (Cortical thickness Ct.Th, internal endosteal diameter ID, and Bone Mineral Density BMD). The ROI was centred at 56% along the length of the femur, distal to the femoral head, to provide a circular cross section avoiding the linea aspera. Trabecular parameters (Trabecular bone volume per tissue volume BV/TV, trabecular number Tb.N, Trabecular thickness Tb.Th, and trabecular spacing Tb.Sp) were calculated from 5μm voxel resolution scans with an ROI consisting of a 1mm long region of the trabecular compartment beginning 100μm proximal to the distal femoral growth plate. For male *Pls3*$^{y/-}$ mice femurs and lumbar vertebrae L5 were imaged. The entire volume of the body of the L5 vertebra was included in the trabecular analysis, with contouring used to exclude the thin layer of cortical bone. Trabecular bone BMD was not included because of the potential for partial volume artifacts due to the broad range of trabecular thickness relative to voxel resolution. Furthermore, cortical porosity (Ct.Po) and canal diameter (Ca.Dm) were determined in a 1 μm voxel resolution scan of a 500 μm ROI centred in the midshaft 56% along the length of the femur distal to the femoral head[15].

Additional cortical vascular and cortical osteocyte lacuna analysis were performed in femur samples from P112 male mice. A 250 μm ROI centered in the midshaft, 56% along the length of the femur distal to the femoral head, was scanned at 1 μm voxel resolution and analysed using previously optimized segmentation thresholds[15]. DICOM images were processed using Fiji and lacunae rendered using its Volume Viewer (https://imagej.nih.gov/ij/plugins/ volume-viewer.html) or Drishti-3.2. Vascular canals and osteocyte lacunae parameters were analysed using BoneJ Particle Analyzer[43]. Particles with a volume >2000 $\mu m^3$ were designated vascular channels and particles between 100 and 2000μm3 were designated osteocyte lacunae[44]. Vascular canal area (Va.Ca.V/BV) and number (VaA.Ca.N/BV) per bone volume, and mean vascular canal volume (Mean Va.Ca.V) in mutants were compared to WT controls. The distribution of vascular canal volumes was compared to WT by randomly selecting the maximum equivalent number vascular canals from each sample (90 per sample) and performing Kolmogorov-Smirnov analysis (GraphPad Prism version 10). Similarly cortical microporosity (Ct.μPo), osteocyte lacunae number per bone volume (Lc.N/BV), mean lacuna volume (Mean Lc.V) in mutants were compared to WT. The distribution of Lacuna volumes was compared to WT by randomly selecting the maximum equivalent number vascular canals from each sample (6275 per sample) and

performing Kolmogorov-Smirnov analysis. Differences in volume distributions between mutant and WT were considered valid if significant in all 10 permutations performed.

**Biomechanical Testing**

An Instron 5543 materials testing load frame (Instron Limited, High Wycombe, UK) was used to perform destructive 3-point bend tests and 2-point compression tests respectively on femurs and caudal vertebrae Ca6 and Ca7 (100N load cell for femurs and 500N load cell for vertebrae) (Instron Limited, High Wycombe, UK) as described[27,45]. Femurs were positioned horizontally with the anterior surface upwards between two custom mount points with rounded ends and a total span of 8mm. Individual vertebrae were bonded in vertical alignment to a custom anvil support using cyanoacrylate glue. Load was applied vertically to the mid-shaft of the femur, or evenly across the vertebral end plate with a constant rate of displacement of 0.03mm/second until femoral fracture, or ~1 mm of displacement had occurred in the vertebra. Biomechanical properties were calculated by plotting load displacement curves for each biomechanical test and determining yield, maximum and, for femurs only, fracture loads. Stiffness was calculated from the slope of the linear portion of the load displacement curve using the "least squares" method. Percentage energy dissipated at fracture load was calculated, as previously described, by subtracting the elastic stored energy at fracture from the total work energy at fracture[40]. The results for caudal vertebrae 6 and 7 were averaged for each parameter. For male *Pls3*$^{y/-}$ mice, adjoining spinous processes were removed from lumbar vertebrae and the vertebral body bonded in vertical alignment to a custom anvil support using cyanoacrylate glue and load was applied vertically at a constant rate of displacement of 0.03 mm/s until approximately 0.5 mm of displacement had occurred. Vertebral yield load, maximum load, and stiffness were derived from load displacement curves.

**Whole-mount skeletal staining**

P1 neonates were prepared and stained with Alizarin red (mineralised bone) and Alcian blue (cartilage) by standard techniques[46]. Whole mount preparations were imaged in glycerol using a Leica MZ75 binocular microscope, Leica KL1500 light source, and Leica DFC320 digital camera (Leica Microsystems Ltd).

**Growth plate histomorphometry**

Tibias were fixed for 24 hours in 10% neutral buffered formalin, decalcified in 10% EDTA pH 7.4 and decalcification was confirmed by X-ray microradiography. Samples were embedded

in paraffin and 5μm sections stained with Alcian blue (cartilage) and van Gieson (osteoid)[47]. Images were obtained using a Leica DM LB2 microscope and DFC320 camera. The width of the proximal tibial growth plate reserve, proliferative, and hypertrophic zones were determined in at least four locations for each section using ImageJ (http://rsb.info.nih.gov/ij/).

**Osteoclast static histomorphometry**

Proximal humeri were fixed for 24 hours in 10% neutral buffered formalin, decalcified in 10% EDTA pH 7.4 and decalcification was confirmed by X-ray microradiography. Samples were embedded in paraffin and 5μm sections stained for tartrate-resistant acid phosphatase (TRAP) (osteoclasts) and counter stained with aniline blue (bone) [48]. Histomorphometry analysis was performed with TrapHisto software (https://www.liverpool.ac.uk/ageing-and-chronic-disease/bone-hist/trap-hist/) in a 750x750 μm area of measurement commencing 250 μm distal to the proximal humeral growth plate[48,49].

**Osteoblast dynamic histomorphometry**

Mice were double-labelled with 15 mg/kg calcein (Sigma C0875) by tail-vein injection of calcein (2.5 μg/mL phosphate-buffered saline) at six and two days prior to collection at P70. Femurs and lumbar vertebrae were embedded in methacrylate and midcoronal block faces were cut and polished to an optically flat surface. To quantify osteoblastic bone formation parameters fluorescent calcein labelling was imaged using confocal autofluorescence scanning light microscopy (CSLM). A Leica SP5 scanning confocal microscope at 488 nm excitation with x40/1.25 objective[50]. Montages of images containing trabecular and cortical endosteal bone surfaces were analysed using ImageJ to determine total bone surfaces, calcein-labelled surfaces and the separation between calcein double-labels according to the American Society for Bone and Mineral Research system of nomenclature[49]. Femoral trabecular bone was analysed in a 1.5x1.5 mm region of interest (ROI) commencing 500 μm proximal to the distal femoral growth plate, and cortical endosteal bone surfaces were analysed 2 mm below the growth plate for a length of 1 mm. Lumbar vertebral trabecular bone was analysed from the entire inner region of a vertebra body from region L1-3 excluding the peripheral cortical bone.

**CD31 Immunohistochemistry**

Femurs, from P21 male mice, were fixed for 24 hours in 10% neutral buffered formalin, decalcified in 10% EDTA pH 7.4 and decalcification was confirmed by X-ray microradiography. Samples were embedded in paraffin and platelet endothelial cell adhesion

molecule (PECAM-1, CD31) immunohistochemistry was performed using 5μm longitudinal midline sections. PECAM-1 antigen retrieval was performed in citrate buffer pH 6 at 60°C for 2h. The primary rabbit anti-CD31 antibody ab182981 (Abcam; Cambridge UK) was diluted 1:200. Visualization was performed using Leica PowerVision Poly-HRP anti-Rabbit IHC Detection System (Leica Biosystems PV6113) according to the manufacturer's instructions. Mouse lung sections were used as a positive control tissue and no primary antibody controls were also included. Sections were counterstained with haematoxylin and imaged using a Leica DM LB2 microscope, Leica Flexacam C1 camera and LAS X software. Images were captured at a resolution of 2016 pixels/mm and full femur montages generated. A 2cm long mid femur cortical bone ROI was defined, and the two cortical ROI images from each sample randomized. ImageJ was calibrated and each cortical ROI image was analysis blind to determine, total cortical area, number of vascular canals, and area of each vascular canal. Individual images were subsequently unblinded and the mean values for the two cortical ROIs form each femur calculated and mutants compared to WT controls.

**Iodine Contrast Enhanced BSE-SEM (ICE-BSE-SEM)**

Formalin fixed femurs, from P70 male mice, were embedded in polymethyl methacrylate (PMMA), polished to an optically flat finish, and stained with potassium iodide. Stock Lugol's Iodine solution (Pro-Lab Diagnostics) was diluted with an equal volume of absolute ethanol and pipetted directly onto the block surface. After 15 min, blocks were washed with distilled water and air dried. Blocks were carbon coated and imaged by SEM (Tescan UK, Cambridge, UK) at high vacuum with a 4-quadrant back-scattered electron detector (Deben,UK) (Male, n=6 per genotype at P70). Images were captured at 20 kV, 0.4 nA and montages of each sample generated at a resolution of 819 pixels/mm. Some higher resolution images were also acquired at 2560 pixels/mm. A 3 cm long cortical bone ROI was defined starting 1cm above the distal femoral growth plate and the two cortical ROI images from each sample randomized. ImageJ was calibrated and each cortical ROI image was analysed blind to determine total cortical area, number of vascular canals, and area of vascular canals. Individual images were subsequently unblinded and the mean values for the two cortical ROIs from each femur calculated and compared to WT controls.

**Serum analysis**

Serum was obtained by centrifugation of blood obtained by terminal cardiac puncture. Serum bone resorption (C-terminal telopeptides of type 1 collagen (CTX)) and bone formation (N-terminal propeptide of type I procollagen (P1NP)) were determined by ELISA at P70

(Immunodiagnostic Systems Ltd, Boldon, Tyne & Wear, UK; AC-06F1, AC-33F1). The inhibitors of Wnt-mediated osteoblastic bone formation Dickkopf related protein-1 (DKK-1) and Sclerostin (SOST) were determined by ELISA at P183 (R&D Systems Europe Ltd, Abingdon, Oxfordshire, UK; MKK100, MSST00).

**OBCD bone phenotyping pipeline quantification and statistical analysis**

***Determination of Wild Type Reference Ranges***

To determine if the KO lines had an abnormal skeletal phenotype, skeletal parameters were compared to those of one of two WT cohorts depending on their genetic background. 918 KO lines were compared to the reference range of the primary WT cohort composed of 320 C57BL/6N and C57BL/6NTac mice. 132 lines were evaluated against the reference range of a secondary WT cohort composed of 80 C57BL/6Brd-$Tyr^{c\text{-}Brd}$ and C57BL/6Dnk mice. Frequency distribution of datasets was assessed by D'Agostino and Pearson test. For the primary WT cohort, the normally distributed parameters were: Femoral Bone Mineral Content, Trabecular Bone Volume per Tissue Volume, Trabecular Number, Cortical Thickness, Cortical Internal Diameter, Femur Yield Load, Maximum Load, and Stiffness, Vertebral Bone Mineral Content, Length, Yield Load, Maximum Load and Stiffness. Non-normally distributed parameters were: Femur Length, Trabecular Thickness, Trabecular Spacing, Cortical Bone Mineral Density, Femur Fracture Load and Toughness (Energy dissipated at fracture). For the secondary WT cohort, the normally distributed parameters were: Femoral Bone Mineral Content, Length, Trabecular Number, Trabecular Thickness Cortical Internal Diameter, Femur Yield Load, Maximum Load, Fracture Load and Stiffness, Vertebral Bone Mineral Content, Length, Yield Load, Maximum Load and Stiffness. Non-normally distributed parameters were: Femur Trabecular Bone Volume per Tissue Volume, Trabecular Spacing, Cortical Thickness, Cortical Bone Mineral Density, and Femur Toughness (Energy dissipated at fracture). Comparisons of WT primary and secondary cohort values with unpaired Student's *t*-test or Kolmogorov-Smirnov tests demonstrated statistically significant differences among the two genetic background strains. All mice analysed during this study are females of age 15.3-16.7 weeks. Statistical analyses and plots for the WT cohorts have been performed using GraphPad Prism 9.

***Reference Range Analysis and Permutation Testing of Outliers***

The Reference Range Analysis involved two distinct approaches to identify KO lines with outlier skeletal phenotypes. Firstly, a KO line was considered as an outlier if the average value of any of the 19 parameters was outside of the WT reference range defined as 2

standard deviations above or below the mean (normally distributed parameters), or outside the 2.5–97.5th percentiles (non-normally distributed parameters). Secondly, for each parameter of each KO line we used permutation testing (100,000 permutations using Julia (https://juliastats.org/) to determine the probability of selecting an equal number of WT samples, from the appropriate cohort, with a parameter value as or more divergent than that observed in the KO line from the WT mean/median. The *P* value was determined by dividing the number times the WT sample's parameter was as or more divergent than that of the KO line by 100,000. Significance thresholds were calculated by applying a Bonferroni correction for the number of effective tests to a $5 \times 10^{-2}$ significance threshold. We determined the number of effective tests by obtaining the eigenvalues of the correlation matrix of the 19 skeletal parameters of the WT baselines using the eigen function in Julia. We then estimated the number of effective tests (Neff) as 11.4 for the primary genetic background and 10.7 for the secondary genetic background using the formula:

$$Neff = N - \sum_{\lambda} I(\lambda > 1) * (\lambda - 1)$$

Where N = 19 as the number of the skeletal parameters and λ indicates the eigenvalues. The outlier *P*-value significance threshold was $4.37 \times 10^{-3}$ for the KO lines on the primary genetic background and $4.69 \times 10^{-3}$ for the KO lines on the secondary genetic background.

***Mahalanobis distance outliers***

To ensure that significant abnormal phenotypes resulting from simultaneous but smaller variances in any of the 19 parameters were not overlooked, we performed a multivariate analysis defining the Squared Robust Mahalanobis distances (MDi2) for each skeletal sample. Only samples with values for all the 19 phenotype parameters were evaluated. The MD¬i2 were computed using the heplots R package (https://friendly.github.io/heplots/reference/Mahalanobis.html) with the minimum volume ellipsoid method for the robust estimate of the centre. We tested for multivariate normality by performing a Mardia's test combining kurtosis and skewness tests (MVN R package) and by visually inspecting the Chi-Square Q-Q plots. Under the assumption of multivariate normality, the distribution of MD¬i2 is approximately chi-squared with p degrees of freedom, with p determined by the number of variables (19). A sample was designated outlier if its MDi2 > χ2p; 0.975 (i.e. MDi2 > 32.852, determined in Microsoft Excel with formula CHISQ.INV(0.975,19)). A KO line was designated a Mahalanobis Outlier if >50% of the KO lines' samples were outliers by Mahalanobis analysis.

***Bone quality outliers***

Using the results from primary or secondary WT cohorts, linear regression analysis was performed comparing femur BMC to each femur strength parameters, and vertebra BMC to each vertebra strength parameters. Lines of best fit, $R^2$, and *P* values were calculated. 95% prediction intervals were determined for all parameter pairs where the linear regression *P*-values was <0.05. The results for KO lines were compared to the 95% prediction intervals. A KO line was designated a Bone Quality Outlier if the mean/median value of any of its parameter pairs were not contained within the WT 95% prediction interval for that parameter pair.

**Statistical analysis of male *Pls3*$^{y/-}$ samples**

Data from hemizygous male *Pls3*$^{y/-}$ mice were compared to male WT littermates by an unpaired two-tailed Student's *t*-test. *P* values < 0.05 were considered statistically significant. The Kolmogorov-Smirnov test was used to compare cumulative frequency distributions of BMC[40,51].

**Zebrafish husbandry**

All zebrafish experimentation adhered to the guidelines of the animal ethics committee of the University of Queensland (Permit 2022/AE000091). Zebrafish transgenic line utilized in this study is *Tg(kdrl:EGFP)*$^{s843}$ [52]. Embryos were obtained through natural paired matings and were incubated at 28°C in a dark-phase incubator. Embryos were maintained in 10 cm petri dishes containing 1X E3 media (5 mM NaCl, 0.17 mM KCl, 0.33 mM $CaCl_2$, 0.33 mM $MgSO_4$) at a maximum density of n=60. At 24 hours post-fertilisation (hpf), all embryos were changed to E3 media supplemented with 0.0003% phenylthiourea (PTU) to prevent pigmentation.

**Genome editing and genotyping of *pls3* mutants in zebrafish**

To generate $G_0$ mosaic crispants, three crRNAs targeting *pls3* were designed using IDT Alt-R Custom Cas9 Design Tool. Sequences of the three crRNAs are included in Supplementary Table 13.

gRNAs were prepared according to the IDT synthesis protocol. In brief, 0.4 μL of each 100 μM crRNA was mixed with 1.2 μL of 100 μM tracrRNA and 0.95 μL of Nuclease-free Duplex Buffer. The mix was incubated at 95°C for 5 minutes and cooled down to room temperature for 3 minutes. Ribonucleoprotein injection mix was prepared with 0.45 μL Alt-R™ S.p. Cas9

Nuclease V3 (100 μg; IDT, 1081058), 3 μL annealed gRNAs cocktail, 2.25 μL 1M KCl, 0.75 μL phenol red and 1.05 μL UltraPure Water, followed by incubating at 37°C for an hour. Zebrafish embryos were injected at the one-cell-stage with ~1 nL of ribonucleoprotein injection mix. Both uninjected control and injected embryos were grown up to 72 hpf for live imaging.
Following live imaging, DNA was extracted from uninjected control and injected embryos, and genotyped to determine cutting efficiency of the gRNAs. Primers sequences for amplification of amplicons flanking crRNA target sites are included in Supplementary Table 13.

**Confocal imaging**
Live zebrafish embryos were mounted laterally in 1% low-melting point agarose and imaged on a Zeiss LSM 710 confocal microscope using a PlnApo 20x/0.8 DICII objective. Image analysis was conducted using Image J. ISV luminal diameter was quantified by application of a Vasometrics macro[53].

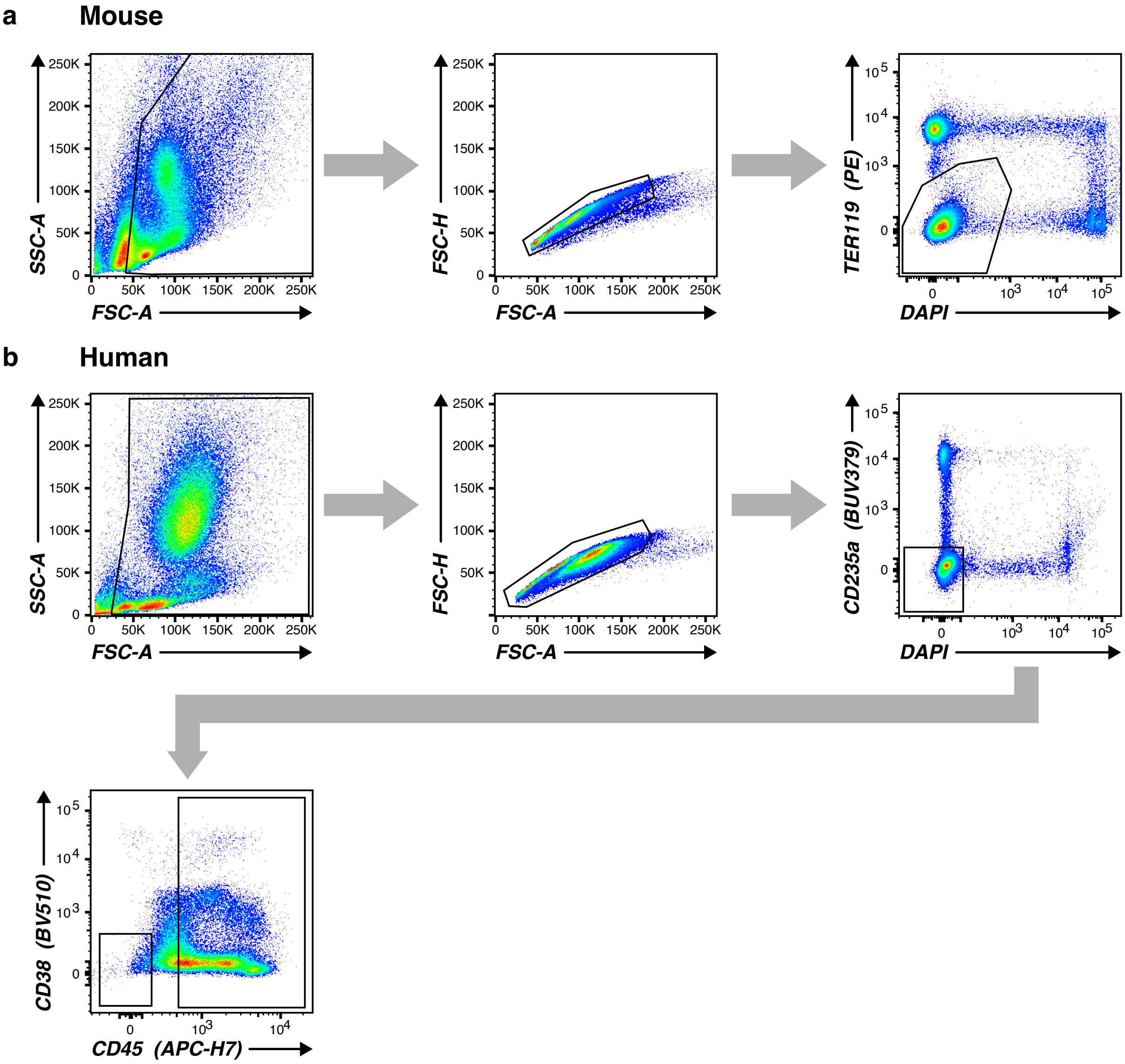


**Supplementary Note 3. FACS gating to collect live non-erythroid cells from mouse and human bones for scRNA-seq.** (a) Gating strategy for collecting live (DAPI-) and non-erythroid cells (TER119-) cells from mouse endosteal bone and marrow cells to generate scRNA-seq data in Figure 1. (b) Gating strategy for collecting live (DAPI-) non-erythroid cells (CD235a-), which were then gated based on CD45- (non-haematopoietic cells) and CD45+ (haematopoietic cells) from human femoral head bone. Non-haematopoietic and haematopoietic cells were pooled in roughly 1:1 ratio for scRNA-seq analysis. This gating strategy was used to generate data in Figure 8.

## References


1. Menezes, S. *et al.* The Heterogeneity of Ly6C(hi) Monocytes Controls Their Differentiation into iNOS(+) Macrophages or Monocyte-Derived Dendritic Cells. *Immunity* **45**, 1205-1218 (2016).
2. Xie, X. *et al.* Single-cell transcriptome profiling reveals neutrophil heterogeneity in homeostasis and infection. *Nat Immunol* **21**, 1119-1133 (2020).
3. Hao, Y. *et al.* Integrated analysis of multimodal single-cell data. *Cell* **184**, 3573-3587.e29 (2021).
4. Lee, R.D. *et al.* Single-cell analysis identifies dynamic gene expression networks that govern B cell development and transformation. *Nat Commun* **12**, 6843 (2021).
5. Korsunsky, I. *et al.* Fast, sensitive and accurate integration of single-cell data with Harmony. *Nat Methods* **16**, 1289-1296 (2019).
6. Troulé, K. *et al.* CellPhoneDB v5: inferring cell–cell communication from single-cell multiomics data. *Nat Protoc* (2025).
7. Drost, H.G. & Paszkowski, J. Biomartr: genomic data retrieval with R. *Bioinformatics* **33**, 1216-1217 (2017).
8. Milacic, M. *et al.* The Reactome Pathway Knowledgebase 2024. *Nucleic Acids Res* **52**, D672-D678 (2024).
9. Yu, G. & He, Q.-Y. ReactomePA: an R/Bioconductor package for reactome pathway analysis and visualization. *Mol BioSyst* **12**, 477-479 (2016).
10. Wickham, H. ggplot2: elegant graphics for data analysis. (Springer-Verlag, New York, 2016).
11. Trapnell, C. *et al.* The dynamics and regulators of cell fate decisions are revealed by pseudotemporal ordering of single cells. *Nat Biotechnol* **32**, 381-386 (2014).
12. Stuart, T. *et al.* Comprehensive Integration of Single-Cell Data. *Cell* **177**, 1888-1902.e21 (2019).
13. Aibar, S. *et al.* SCENIC: single-cell regulatory network inference and clustering. *Nat Methods* **14**, 1083-1086 (2017).
14. Ashburner, M. *et al.* Gene Ontology: tool for the unification of biology. *Nat Genet* **25**, 25-29 (2000).
15. Youlten, S.E. *et al.* Osteocyte transcriptome mapping identifies a molecular landscape controlling skeletal homeostasis and susceptibility to skeletal disease. *Nat Commun* **12**, 2444 (2021).
16. Smith, C.L., Blake, J.A., Kadin, J.A., Richardson, J.E. & Bult, C.J. Mouse Genome Database (MGD)-2018: knowledgebase for the laboratory mouse. *Nucleic Acids Res* **46**, D836-d842 (2018).
17. Durinck, S., Spellman, P.T., Birney, E. & Huber, W. Mapping identifiers for the integration of genomic datasets with the R/Bioconductor package biomaRt. *Nat Protoc* **4**, 1184-1191 (2009).
18. Zimmerman, M.T., Kabat, B., Grill, D.E., Kennedy, R.B. & Poland, G.A. RITAN: rapid integration of term annotation and network resources. *PeerJ* **7**(2019).
19. Unger, S. *et al.* Nosology of genetic skeletal disorders: 2023 revision. *Am J of Med Genet A* **191**, 1164-1209 (2023).
20. Shannon, P. *et al.* Cytoscape: a software environment for integrated models of biomolecular interaction networks. *Genome Res* **13**, 2498-504 (2003).
21. Morris, J.A. *et al.* An atlas of genetic influences on osteoporosis in humans and mice. *Nat Genet* **51**, 258-266 (2019).
22. Deng, J.E., Sham, P.C. & Li, M.X. SNPTracker: A Swift Tool for Comprehensive Tracking and Unifying dbSNP rs IDs and Genomic Coordinates of Massive Sequence Variants. *G3 (Bethesda)* **6**, 205-7 (2015).

23. Cingolani, P. *et al.* A program for annotating and predicting the effects of single nucleotide polymorphisms, SnpEff: SNPs in the genome of Drosophila melanogaster strain w1118; iso-2; iso-3. *Fly (Austin)* **6**, 80-92 (2012).
24. McLaren, W. *et al.* The Ensembl Variant Effect Predictor. *Genome Biol* **17**, 122 (2016).
25. Yang, J. *et al.* Conditional and joint multiple-SNP analysis of GWAS summary statistics identifies additional variants influencing complex traits. *Nat Genet* **44**, 369-75, s1-3 (2012).
26. Yang, J., Lee, S.H., Goddard, M.E. & Visscher, P.M. GCTA: a tool for genome-wide complex trait analysis. *Am J Hum Genet* **88**, 76-82 (2011).
27. Kemp, J.P. *et al.* Identification of 153 new loci associated with heel bone mineral density and functional involvement of GPC6 in osteoporosis. *Nat Genet* **49**, 1468-1475 (2017).
28. Bycroft, C. *et al.* The UK Biobank resource with deep phenotyping and genomic data. *Nature* **562**, 203-209 (2018).
29. Manichaikul, A. *et al.* Robust relationship inference in genome-wide association studies. *Bioinformatics* **26**, 2867-73 (2010).
30. Purcell, S. *et al.* PLINK: a tool set for whole-genome association and population-based linkage analyses. *Am J Hum Genet* **81**, 559-75 (2007).
31. Quinlan, A.R. & Hall, I.M. BEDTools: a flexible suite of utilities for comparing genomic features. *Bioinformatics* **26**, 841-2 (2010).
32. Schaum, N. *et al.* Single-cell transcriptomics of 20 mouse organs creates a Tabula Muris. *Nature* **562**, 367-372 (2018).
33. Consortium, T.T.S. & Quake, S.R. Tabula Sapiens reveals transcription factor expression, senescence effects, and sex-specific features in cell types from 28 human organs and tissues. *bioRxiv*, 2024.12.03.626516 (2025).
34. Huang, N. sceasy: A package to help convert different single-cell data formats to each other. 0.0.7 edn (2025).
35. Uhlén, M. *et al.* Tissue-based map of the human proteome. *Science* **347**, 1260419 (2015).
36. Kolde, R. & Kolde, M.R. Package ‘pheatmap’. *R package* **1**, 790 (2015).
37. Kilkenny, C., Browne, W.J., Cuthill, I.C., Emerson, M. & Altman, D.G. Improving bioscience research reporting: the ARRIVE guidelines for reporting animal research. *PLoS Biol* **8**, e1000412 (2010).
38. Skarnes, W.C. *et al.* A conditional knockout resource for the genome-wide study of mouse gene function. *Nature* **474**, 337-42 (2011).
39. White, J.K. *et al.* Genome-wide generation and systematic phenotyping of knockout mice reveals new roles for many genes. *Cell* **154**, 452-64 (2013).
40. Bassett, J.H. *et al.* Rapid-throughput skeletal phenotyping of 100 knockout mice identifies 9 new genes that determine bone strength. *PLoS Genet* **8**, e1002858 (2012).
41. Butterfield, N.C., Logan, J.G., Waung, J., Williams, G.R. & Bassett, J.H.D. Quantitative X-Ray Imaging of Mouse Bone by Faxitron. *Methods Mol Biol* **1914**, 559-569 (2019).
42. Butterfield, N.C. *et al.* Accelerating functional gene discovery in osteoarthritis. *Nat Commun* **12**, 467 (2021).
43. Doube, M. *et al.* BoneJ: Free and extensible bone image analysis in ImageJ. *Bone* **47**, 1076-1079 (2010).
44. Hemmatian, H. *et al.* Age-related changes in female mouse cortical bone microporosity. *Bone* **113**, 1-8 (2018).
45. Esapa, C.T. *et al.* Bone Mineral Content and Density. *Curr Protoc Mouse Biol* **2**, 365-400 (2012).
46. Rigueur, D. & Lyons, K.M. Whole-mount skeletal staining. *Methods Mol Biol* **1130**, 113-121 (2014).

47. Bassett, J.H. *et al.* Mice lacking the calcineurin inhibitor Rcan2 have an isolated defect of osteoblast function. *Endocrinology* **153**, 3537-48 (2012).
48. van 't Hof, R.J., Rose, L., Bassonga, E. & Daroszewska, A. Open source software for semi-automated histomorphometry of bone resorption and formation parameters. *Bone* **99**, 69-79 (2017).
49. Dempster, D.W. *et al.* Standardized nomenclature, symbols, and units for bone histomorphometry: a 2012 update of the report of the ASBMR Histomorphometry Nomenclature Committee. *J Bone Miner Res* **28**, 2-17 (2013).
50. Bassett, J.H. *et al.* Thyrostimulin Regulates Osteoblastic Bone Formation During Early Skeletal Development. *Endocrinology* **156**, 3098-113 (2015).
51. Bassett, J.H., van der Spek, A., Gogakos, A. & Williams, G.R. Quantitative X-ray imaging of rodent bone by Faxitron. *Methods Mol Biol* **816**, 499-506 (2012).
52. Beis, D. *et al.* Genetic and cellular analyses of zebrafish atrioventricular cushion and valve development. *Development* **132**, 4193-204 (2005).
53. McDowell, K.P., Berthiaume, A.A., Tieu, T., Hartmann, D.A. & Shih, A.Y. VasoMetrics: unbiased spatiotemporal analysis of microvascular diameter in multi-photon imaging applications. *Quant Imaging Med Surg* **11**, 969-982 (2021).
