# Supplementary material for: Multiscale analysis and functional validation of the cellular and genetic determinants of skeletal disease
Source: Nat Genet. 2026 Jul 10;58(7):1530–45. doi: 10.1038/s41588-026-02640-9 (PMC13364720; doi:10.1038/s41588-026-02640-9)
Supplement: Supplementary file 2 — Reporting Summary [file 41588_2026_2640_MOESM2_ESM.pdf]

nature portfolio

Corresponding author(s): Dr. John Kemp

Last updated by author(s): Feb 10, 2026

Reporting Summary

Nature Portfolio wishes to improve the reproducibility of the work that we publish. This form provides structure for consistency and transparency in reporting. For further information on Nature Portfolio policies, see our Editorial Policies and the Editorial Policy Checklist.

Statistics

For all statistical analyses, confirm that the following items are present in the figure legend, table legend, main text, or Methods section.

n/a	Confirmed	
☐	☒	The exact sample size (*n*) for each experimental group/condition, given as a discrete number and unit of measurement
☐	☒	A statement on whether measurements were taken from distinct samples or whether the same sample was measured repeatedly
☐	☒	The statistical test(s) used AND whether they are one- or two-sided<br>*Only common tests should be described solely by name; describe more complex techniques in the Methods section.*
☐	☒	A description of all covariates tested
☐	☒	A description of any assumptions or corrections, such as tests of normality and adjustment for multiple comparisons
☐	☒	A full description of the statistical parameters including central tendency (e.g. means) or other basic estimates (e.g. regression coefficient) AND variation (e.g. standard deviation) or associated estimates of uncertainty (e.g. confidence intervals)
☐	☒	For null hypothesis testing, the test statistic (e.g. *F*, *t*, *r*) with confidence intervals, effect sizes, degrees of freedom and *P* value noted<br>*Give P values as exact values whenever suitable.*
☒	☐	For Bayesian analysis, information on the choice of priors and Markov chain Monte Carlo settings
☒	☐	For hierarchical and complex designs, identification of the appropriate level for tests and full reporting of outcomes
☐	☒	Estimates of effect sizes (e.g. Cohen's *d*, Pearson's *r*), indicating how they were calculated

Our web collection on statistics for biologists contains articles on many of the points above.

Software and code

Policy information about availability of computer code

Data collection

1. Collection of cells for single cell RNAseq (scRNA-seq) and single nucleus ATACseq (snATAC-seq)
Processing of scRNA-seq raw data using Cell Ranger versions 2 to 7
Processing of snATAC-seq raw data using Cell Ranger version 2

2. Data from the UK Biobank Study were downloaded via their FTP protocols

3. Human protein coding gene annotations ENSEMBLE Gene v105 (GRCh38)
https://asia.ensembl.org/biomart/

4. Genomic co-ordinates and rsids for GRCh38 dbSNP b151
https://ftp.ncbi.nih.gov/snp/organisms/human_9606_b151_GRCh38p7/database/organism_data/

5. Published BMD SNP associations from NGRI GWAS Catalog
https://www.ebi.ac.uk/gwas/efotraits/EFO_0003923

6. Nosology of genetic skeletal disorders: 2023 Revision
DOI: 10.1002/ajmg.a.63132

7. Mouse Genome Informatics Data
Phenotype ontology terms (http://www.informatics.jax.org/vocab/mp_ontology/)
Genes associated with ontology terms (http://www.informatics.jax.org/marker/)

8. For mouse functional phenotyping experiments in the Origins of Bone and Cartilage Diseases (OBCD) study:
NRecon (Bruker, http://bruker-microct.com/products/downloads.html)
CTAn (Bruker, http://bruker-microct.com/products/downloads.html)
Scanco (v6.4-2)
Xming 6.9.0.31 (Colin Harrison; http://www.straightrunning.com/XmingNotes/)
PuTTY 0.62 (Simon Tatham; https://www.putty.org)
Instron Bluehill Universal (v4.23) (https://www.instron.com/en/products/materials-testing-software/bluehill-universal)
Drishti-2 (v2.6.1, https://github.com/nci/drishti, RRID:SCR_017999)
Aperio Imagescope (Leica, https://leicabiosystems.com/digital-pathology/manage/aperio-imagescope)
ImageJ (https://imagej.net, RRID:SCR_003070)
TrapHisto (https://www.liverpool.ac.uk/ageing-and-chronic disease/bone-hist/trap-hist/)

Data analysis

1. scRNA-seq analysis was performed using Seurat (versions 2-5), Monocle 2 (version 2.2.0), pySCENIC (version 0.11.2) and CellPhoneDB (version 5.0.1)
Graphs for scRNA-seq analysis were plotted using Seurat and ggplot2 (version 3.4.2)
Human orthologs of mouse genes were annotated using bioMaRt (version 2.56.1)

2. snATAC-seq analysis was performed using Signac (version 1.15.0), Seurat (version 5.3.0), JASPAR2020 (version 0.99.10) and presto (version 1.0.0)
Graphs for snATAC-seq analysis were plotted using Seurat and ggplot2 (version 3.4.2)

3. Genetic association and enrichment analyses
BOLT-LMM (version 2.3.4), GCTA (version 1.93.2), LDSC (version 1.0.1), BGENIX, R, RStudio, SNPTracker (version 1.0), SnpEff (version 5.1d), VEP, Plink (version1.9), Plink (version 2.0), Bedtools (version 2.29.2), Bash, Gawk, MAGMA (version 1.10),
GraphPad Prism 10, RITAN (version 1.24.0), Cytoscape (version 3.10.0), KING (implemented in Plink version 1.9).

4. Statistical analyses and plots for the wild type baselines in the OBCD phenotyping study were performed and generated using GraphPad Prism 9 and 10 (GraphPad Software Inc; RRID:SCR_002798)

All single-cell, spatial transcriptomic and MAGMA analyses were performed using standard, publicly available packages. The custom R scripts are available at (doi.org/10.5281/zenodo.18529624) and upon reasonable request.

For manuscripts utilizing custom algorithms or software that are central to the research but not yet described in published literature, software must be made available to editors and reviewers. We strongly encourage code deposition in a community repository (e.g. GitHub). See the Nature Portfolio guidelines for submitting code & software for further information.

Data

Policy information about availability of data

All manuscripts must include a data availability statement. This statement should provide the following information, where applicable:

- Accession codes, unique identifiers, or web links for publicly available datasets
- A description of any restrictions on data availability
- For clinical datasets or third party data, please ensure that the statement adheres to our policy

Source data is available in Supplementary Tables, Source Data section and in online repositories. The raw scRNAseq and snATAC-seq data has been deposited on the Gene Expression Omnibus (GEO) database under the accession number GSE317069.

Genome-wide association summary result statistics is available via the NHGRI-EBI GWAS Catalog (https://www.ebi.ac.uk/gwas/) with the accession number GCST90726625.

Spatial transcriptomics data of human bone from Yip et al (2025) were accessed from the Gene Expression Omnibus under accession number GSE299207.

Research involving human participants, their data, or biological material

Policy information about studies with human participants or human data. See also policy information about sex, gender (identity/presentation), and sexual orientation and race, ethnicity and racism.

Reporting on sex and gender

Analyses of human genetic data include both males and females. Genetically inferred sex was utilized as a covariable to adjust for differences in estimated bone mineral density

Analysis of human scRNA-seq data include both males and females.

Reporting on race, ethnicity, or other socially relevant groupings

Our study did not report on socially relevant groupings. Instead genetically inferred ancestry was used to control for population stratification.

Population characteristics

Descriptive statistics of UK-Biobank Study participants are presented in Supplementary Table 7.

	Description of human research participants for scRNA-seq analysis are summarised in Supplementary Table 11 to include age, gender and primary diagnosis (reason for elective surgery).
Recruitment	Details of subject recruitment to the UK-Biobank Study are described in the methods, and available from the UK-Biobank website (https://www.ukbiobank.ac.uk/)<br><br>Femoral head bone samples were obtained from patients with osteoarthritis undergoing total hip arthroplasty at St. Vincent's Hospital Sydney. All participants provided written informed consent, and the study was conducted with approval from the St. Vincent's Hospital Human Research Ethics Committee (2022/ETH00475).
Ethics oversight	Our study has been approved by an appropriate Institutional Review Boards. The UK-Biobank Study obtained appropriate ethics approval as described in the methods and available from the UK-Biobank website (https://www.ukbiobank.ac.uk/).<br><br>Human bone samples for scRNA-seq were collected with approval from the St. Vincent's Hospital Sydney Human Research Ethics Committee (2022/ETH00475).

Note that full information on the approval of the study protocol must also be provided in the manuscript.

Field-specific reporting

Please select the one below that is the best fit for your research. If you are not sure, read the appropriate sections before making your selection.

☒ Life sciences ☐ Behavioural & social sciences ☐ Ecological, evolutionary & environmental sciences

For a reference copy of the document with all sections, see nature.com/documents/nr-reporting-summary-flat.pdf

Life sciences study design

All studies must disclose on these points even when the disclosure is negative.

Sample size	1. For scRNA-seq endosteal bone cells and marrow cells were isolated and captured using 10x Genomics from femurs of 5 groups (endosteal) and 4 groups (marrow) of mice. Each group consisted of 5 mice. No power calculation was performed to pre-determine the sample size. Cells from each of the 4-5 groups of mice were captured with a 10x Genomics run, yielding between 5636-14881 cells each. In total 133,942 cells were included in the analysis. This number was adequate to detect rare populations, such as early osteocytes with 35 cells detected or 0.026% of all cells analysed.<br><br>2. For genetic association analyses. Data from 448,010 participants of the UK Biobank Study were selected based on stringent quality control criteria. Participants were selected if they had high-quality quantitative heel ultrasound and genotyping data, and if their genetically inferred ancestry was predominantly European.<br><br>3. The coefficient of variation for each of the OBCD skeletal phenotyping parameters were precisely defined using 400 WT mouse samples from two different genetic backgrounds. Power calculations indicate an 80% power to detect outlier phenotype of greater or equal to 2 s.d. with a sample size of n=2 for lines in the OBCD screen.<br><br>Power calculations using WT data together with the magnitude of the skeletal phenotype identified female Pls3-/- mice in the OBCD screen (n=2) demonstrated that n=7-8 was required for the comparison of Pls3y/- mice and WT littermates.<br><br>10 other KO lines have been selected from the OBCD screen for further validation by rederivation into independent colonies. Analysis of mice from these colonies have successfully replicated the skeletal phenotypes from the screen. This includes four lines that have been published (Gpc6, Daam2, Cadm1, Sparc).
Data exclusions	1. For scRNA-seq all cells with less than 300 distinct genes observed, or cells with more than 10% of genes mitochondrial genes were removed from analysis as described in methods.<br><br>2. For genetic association analyses, we excluded participants if they were missing outcomes and covariates pertinent to the association testing. This is because participants lacking covariates for association testing cannot be fit properly to our mixed-model approach. We also manually filtered for obvious outliers by observing the distributions of the data and removing individuals far exceeding the tail ends of the data. SNPs were excluded from analysis in accordance with our QC criteria which are described in methods.<br><br>3. In the OBCD mouse knockout studies, data for all mice were included, including those with and without skeletal phenotypes
Replication	Experiments were conducted using multiple independent biological replicates as outlines in the 'Sample size' section above, and in results and methods section of the manuscript.<br>1. For scRNA-seq, the captures were conducted using 4 (marrow) or 5 (endosteal bone) independent biological replicates as outlined in results and methods. All annotated cell types and sub-types were replicated and identified in these independent biological replicates.<br><br>2. For genetic association analyses, associations with estimated bone mineral density (eBMD) could not be replicated as independent cohorts with eBMD and genetics data are too small to conduct well powered replication studies. To accommodate this our reporting criteria was adjusted accordingly, and we used a more stringent GWAS significance threshold of 6.6x10^-9.<br><br>3. In the mouse and zebrafish knockout studies, the exact number of biological replicates examined for each knockout line is specified in results.

The OBCD skeletal phenotyping screen has been developed, validated using skeletal samples from 400 WT mice of two different genetic backgrounds with continuous WT sample calibration over >10 years. X-ray microradiography and micro-CT equipment was also calibrated weekly over this period. Skeletal parameters were determined once for each skeletal sample and not replicated.
Abnormalities identified in female Pls3-/- mice in the OBCD screen (n=2) were replicated in male and female Pls3 KO mice rederived at Imperial College.

Abnormalities of the skeletal vasculature identified in Pls3y/- mice by CD31 immunohistochemistry were replicated and validated using two different analytical methods Iodine contrast enhanced BSE-SEM and high-resolution micro-computerised tomography (µCT)

10 other KO lines have been selected from OBCD screen for further validation by rederivation into independent colonies. Analysis of mice from these colonies have successfully replicated the skeletal phenotypes. This includes four lines that have been published (Gpc6, Daam2, Cadm1, Sparc).

Randomization

1. For scRNAseq samples were collected in 4-5 groups of 5 mice (one 10x Genomics capture for each group of mice). No significant batch effect was detected between the 4-5 independent runs.

2. Genetic association analyses: Participants were recruited at various sites through the UK without any selection criteria. We do adjust for genotyping chip in our association studies, as this assignment was not random.

3. For the OBCD rapid throughput phenotyping of knockout mouse lines - Following the principles of the 3Rs mice were assigned to age groups on the basis of their date of birth. Oldest cohorts first youngest cohorts being the final litters. There were no experimental groups in the skeletal studies.

Blinding

1. Not relevant in the scRNA-seq analysis.

2. Not relevant to our genetic association analyses as the participants represent the general population of the UK.

3. In all skeletal phenotyping analyses investigators were blinded to genotype until statistical comparisons were undertaken

Reporting for specific materials, systems and methods

We require information from authors about some types of materials, experimental systems and methods used in many studies. Here, indicate whether each material, system or method listed is relevant to your study. If you are not sure if a list item applies to your research, read the appropriate section before selecting a response.

Materials & experimental systems

n/a	Involved in the study	
☐	☒	Antibodies
☒	☐	Eukaryotic cell lines
☒	☐	Palaeontology and archaeology
☐	☒	Animals and other organisms
☐	☒	Clinical data
☒	☐	Dual use research of concern
☒	☐	Plants

Methods

n/a	Involved in the study	
☒	☐	ChIP-seq
☐	☒	Flow cytometry
☒	☐	MRI-based neuroimaging

Antibodies

Antibodies used

Fc block; clone 93; catalogue number 101302; Biolegend; 1ul per sample
TER119-PE; clone TER-119; catalogue number 116208; Biolegend; 1:100 dilution
CD235-BUV395; clone GA-R2; catalogue number 563810; BD Biosciences; 1:100 dilution
CD45-APC-H7; clone 2D1; catalogue number 641408; BD Biosciences; 1:100 dilution
ZombieNIR Viability stain; catalogue number 423106; Biolegend; 1:500 dilution
B220-BV510; clone RA3-6B2; catalogue number 103248; Biolegend; 1:200 dilution
TCRb-BV510; clone GL3; catalogue number 118131; Biolegend; 1:200 dilution
CD45-BV650; clone 30-F11; catalogue number 103151; Biolegend; 1:200 dilution
CD11b-BUV395; clone M1/70; catalogue number 565976; BD Biosciences; 1:200 dilution
Ly6C-BUV737; clone HK1.4.rMAb; catalogue number 755201; BD Biosciences; 1:200 dilution
Ly6G-APC; clone 1A8; catalogue number 560599; BD Biosciences; 1:200 dilution
CD14-PE; clone Sa14-2; catalogue number 569968; BD Biosciences; 1:200 dilution
CD31 primary antibody for IHC; clone EPR17259; catalogue number ab182981; 1:200 dilution

Validation

All antibodies used were commercially available and were validated by the manufacturers. No additional validation experiments were performed. Details on validation experiments can be found on the website for each of the catalogue numbers listed.

Animals and other research organisms

Policy information about studies involving animals; ARRIVE guidelines recommended for reporting animal research, and Sex and Gender in Research

Laboratory animals	1. For scRNA-seq and snATAC-seq experiments, cells were isolated from 9-10-week-old male C57BL/6J mice obtained from Australian Bioresources. Animal holding areas were maintained within a constant temperature range of 21±2 °C and 55±10% humidity. Animals were kept in individually ventilated cages in groups of 2-5 per cage, provided with environmental enrichment and had ad libitum access to sterilised water and a standard irradiated rodent chow.<br><br>2. Not relevant to our genetic association analyses<br><br>3. OBCD phenotyping study<br>Knockout mouse lines screened in the OBCD phenotyping pipeline were produced on a background of C57BL/6Brd-Tyrc-Brd, C57BL/6Dnk, and C57BL/6N mice, as part of the Wellcome Trust Sanger Institute's (WTSI) Mouse Genetics Project (MGP), part of the International Mouse Phenotyping Consortium (IMPC;http://www.mousephenotype.org). Phenotype analyses were performed on skeletal samples from 16-week-old female mice.<br><br>C57BL/6N mice carrying a Pls3 tm1a(EUCOMM)Wtsi knockout first allele (MGI:104807) were obtained from the Welcome Trust Sanger Institute EMMA mouse repository and re-derived from frozen embryos. Male hemizygous mice (Pls3y/-) were phenotyped at ages P21, P70 and P183.<br><br>Animal holding areas were maintained within a constant temperature range of 21±2 °C and 55±10% humidity. Animals were kept in individually ventilated cages in groups of 2-5 per cage, provided with environmental enrichment and had ad libitum access to sterilised water and a standard irradiated rodent chow.
Wild animals	n/a
Reporting on sex	The sex of the animals is detailed in results and methods sections.
Field-collected samples	n/a
Ethics oversight	1. All experiments and procedures involving mice for scRNA-seq and snATAC-seq were conducted in accordance with procedures and protocols approved by by the Garvan Institute of Medical Research Animal Ethics Committee (ARA16/01, ARA19/09 and ARA22/12).<br><br>2. Not relevant to our genetic association analyses<br><br>3. All OBCD mouse skeletal phenotyping studies were undertaken under licence at Imperial College (project licence PPL70/8785 and PP1540664) and the Wellcome Trust Sanger Institute Mouse Genetics Project as part of the International Mouse Phenotyping Consortium and licenced by the UK Home Office (PPLs 80/2485 and P77453634) in accordance with the 1986 Animals (Scientific Procedures) Act and the recommendations of the Weatherall report. Animal experiments were approved by the Sanger or Imperial College Hammersmith Campus Animal Welfare Ethical Review Bodies (AWERB) as appropriate.

Note that full information on the approval of the study protocol must also be provided in the manuscript.

Clinical data

Policy information about clinical studies

All manuscripts should comply with the ICMJE guidelines for publication of clinical research and a completed CONSORT checklist must be included with all submissions.

Clinical trial registration	n/a
Study protocol	n/a
Data collection	n/a
Outcomes	n/a

Plants

Seed stocks

n/a

Novel plant genotypes

n/a

Authentication

n/a

Flow Cytometry

Plots

Confirm that:

☒ The axis labels state the marker and fluorochrome used (e.g. CD4-FITC).

☒ The axis scales are clearly visible. Include numbers along axes only for bottom left plot of group (a 'group' is an analysis of identical markers).

☒ All plots are contour plots with outliers or pseudocolor plots.

☒ A numerical value for number of cells or percentage (with statistics) is provided.

Methodology

Sample preparation

To obtain endosteal compartment and bone marrow cells for scRNA-seq, mice were sacrificed via CO2 asphyxia. Femurs were harvested from 5 groups of 5 mice. Soft tissue and epiphysis were removed from the femurs before being separated into diaphysis and metaphysis. Marrow cells were collected by flushing the diaphysis with PBS. Marrow-depleted diaphyseal and metaphyseal bone were crushed and cut up gently, then cells that are adherent to the endosteal surface were removed by digestion using 2mg/ml of collagenase A and 2.5mg/ml of trypsin for 30 mins at 37ºC. After digestion, bone fragments were vortexed for 10s and the supernatant containing digested endosteal cells was filtered through a 100µm filter into collection tubes containing 10% fetal calf serum (FCS; Bovogen Biologicals). Marrow cells and endosteal cells were collected by centrifugation at 400x g for 5 mins and resuspended in 200µl PBS supplemented with 2% FCS prior to staining for FACS sorting. Cells were stained for relevant antibodies at 4ºC for 30 mins and rinsed with PBS supplemented with 2% FCS. Dead cells and debris were excluded by FSC, SSC and DAPI (ThermoFisher Scientific) or ZombieNIR.

Instrument

Cell sorting was done using BD Aria II and Aria III sorters. Flow validation experiments were performed using BD FACSymphony machine

Software

FlowJo (Version 10.10.0)

Cell population abundance

We were able to collect or sort more than enough (>100,000) live non-erythroid cells for each experiment.

Gating strategy

Gating strategy for scRNA-seq experiments are shown in Supplementary Note 3
Gating strategy for validation experiments are shown in Supplementary Figure 1

☒ Tick this box to confirm that a figure exemplifying the gating strategy is provided in the Supplementary Information.
