# Supplementary material for: Multiscale analysis and functional validation of the cellular and genetic determinants of skeletal disease
Source: Nat Genet. 2026 Jul 10;58(7):1530–45. doi: 10.1038/s41588-026-02640-9 (PMC13364720; doi:10.1038/s41588-026-02640-9)
Supplement: Supplementary file 3 — Peer Review File [file 41588_2026_2640_MOESM3_ESM.pdf]

nature portfolio

Peer Review File

Multiscale analysis and functional validation of the cellular and genetic determinants of skeletal disease

Corresponding Author: Dr John Kemp

A version of this paper was originally rejected for publication by Nature Genetics, however that decision was reconsidered after appeal by the authors.

Version 0:

Decision Letter:

7th Feb 2025

Dear John,

hope this email finds you well, and sorry for the delay in providing the reviews.

Your Article entitled "Multiscale analysis and functional validation of the cellular and genetic determinants of skeletal disease" has now been seen by 2 referees, whose comments are attached. While they find your work of potential interest, they have raised serious concerns which in our view are sufficiently important that they preclude publication of the work in Nature Genetics, at least in its present form.

Reviewer #1 appreciates the resource value but highlights some limitations of the study, i.e., the lack of a scATAC-seq dataset, and the missed opportunity to analyze adipose tissues and cell-cell communication. They are also concerned that two replicates for the mouse work are not sufficient to make strong claims.

Reviewer #2 acknowledges that the study provides some new resources and insights into skeletal diseases, but they think most results would require stronger mechanistic explorations. They also believe you should include adipocytes in your analysis.

Should further experimental data allow you to fully address these criticisms we would be willing to consider an appeal of our decision (unless, of course, something similar has by then been accepted at Nature Genetics or appeared elsewhere). Both reviewers have emphasized the importance of analyzing adipocyte tissue; therefore, we recommend closely examining this aspect.

The required new experiments and data include, but are not limited to those detailed here. We hope you understand that until we have read the revised manuscript in its entirety we cannot promise that it will be sent back for peer review.

If you are interested in attempting to revise this manuscript for submission to Nature Genetics in the future, please contact me to discuss a potential appeal. Otherwise, we hope that you find our referees' comments helpful when preparing your manuscript for resubmission elsewhere.

Thank you and sorry again for the delay.

Best wishes,
Chiara

Chiara Anania, PhD
Associate Editor
Nature Genetics
https://orcid.org/0000-0003-1549-4157

Referee expertise:

Referee #1: bone biology, mouse model

Referee #2: genetics, osteoporosis

Referee #3:

Reviewers' Comments:

Reviewer #1 (Remarks to the Author):

A. Summary of the key results

This study started with a scRNASeq analysis of mouse long bones, using bone marrow and diaphyseal and metaphyseal endosteal bone as compartment sources. The authors identified gene expression sets for hematopoietic and nonhematopoeitic cells, the latter derived primarily from the two endosteal compartments. They annotated the distinct gene profiles, which in part matched known markers of the respective cell types (osteoblasts, chondrocytes, endothelial cells, etc.) and went into the analysis of the nonhematopoietic cell types in detail. They predicted potential transcriptional regulators with the Scenic database and annotated the majority of genes not known to be associated with the skeleton.
In a next step, the authors integrated the cell type specific genes with those known for monogenic skeletal diseases. Not so surprisingly, within osteoblasts, chondrocytes, and osteoclasts the expected associated monogenetic disorder associated genes were annotated, confirming that those disease genes are indeed expressed in these cell types. Strikingly, some genes associated with disorders, e.g. osteoclerosis genes, were found rather in osteoblast progenitor cells and early osteocytes, which was a priori not expected. Surprisingly, genes associated with B cells and monocytes were associated with dwarfism, which is rather linked to chondrocyte function. Thismay reflect the cell cycle in general as the authors noted, but also shows the limitation of the interpretation of this approach.
The authors subsequently presented a novel GWAS from the UK Biobank to identify variants of endosteal bone mineral density. This includes fine mapping that identify gene loci that in part are associated with rare genetic skeletal disorders, but also novel gene variants.
Human orthologues identified by the GWAS were found to be expressed in murine nonhematopoietic cell types, particular osteoblasts, chonrdocyte, endothlial cells, and VSMC, thereby linking GWAS candidate genes to expression in these murine cells.
Of these 1,347 protein-coding genes were found to cause abnormal bone structure in the respective mutant mice in the MGI database. Fine mapping showed that genes in particular associated with eBMD in humans were highly enriched as bone penotype causing mutations in the MGI database, which was not only caused by genes causing rare skeletal dysplasia.
The genes annotated to osteoblasts, chondrocytes, endothlial cells, osteoclasts and VSMC were subsequently analyzed by an analysis platform OBCD. Here, effects of loss-of-functions in part heterozygous on the skeleton, with roughly half showing structural and/or functional bone phenotypes. The authors show some analyses in detail, in particular Pls3y / - showing a loss of trabecular and cortical bone. Since specific bone cell parameters were not changed, they conclude that Pls3 function in multiple cell types might be involved in maintaining bone structure. They analyze knockout mice for genes expressed predominantly in either cell cluster of the aforementioned nonhematopoietic cells and show that they in part have structural or functional (e.g. mechanical) impairments. Finally, an online interactive platform is presented to search for genes involved in bone cell scRNASeq data sets, eBMD human phenotypes and phenotype in knockout mice.

B. Originality and significance

This is an intriguing resource combining scRNASeq data with human genetic traits and loss of function studies in more than 1000 mouse lines to characterize genes and genetic factors involved in bone diseases. This very comprehensive translational approach to link data from a model organism to human phenotypes in a systematic manner is of high value for the basic scientific understanding of bone integrity and also for the identification of novel drug targets. While this is novel in its holistic approach, many single results are in part expected, by revealing known genes associated with skelelal biology and if not associated with skeletal diseases associating them with bone phenotypes in the MGI databases. However, this confirms the validity of the approach taken here and underscores the valuable resource for the research community that had been generated. This study is well suited for Nature Genetics, given some further analyses are under taken.

C. Data & methodology: validity of approach, quality of data, quality of presentation

The methodology reflects state of the art handling or scRNASeq data including annotation of canoncial marker genes to annotate the UMAP cluster. The data was reanalyzed by the student of the reviewer in part. The quality indicates a high viability of the cells, which is not trivial for digested bone tissue. The re-analysis revealed a slightly reduced amount of clusters, which might be due to a different Seurat version used, but overall the conclusions are compatible. The suggestions of involved transcription factors by the Scenic algorithm is an approximation but would benefit from a scATAC Seq for validity. Therefore, the data should be presented as more suggestive as currently stated. The GWAS analyses I personally

cannot judge technically but appear plausible. The presentation of the data is appropriate, some details could be more comprehensively described, but overall, however, the complex data are excellently presented.

D. Appropriate use of statistics and treatment of uncertainties

A challenge is the low number of mutant mice used in the assessment of bone parameters. While this is intrinsic by testing so many mutants, n = 2, despite having a large n for wt mice, this remains to be improved and the data taken with caution. In the detailed analysis the group size is slightly higher (n=4 for p21 and PLS KO), but still too low for conclusions. At later ages of PLS3 KO mice (P183) the group size is appropriate.

E. Conclusions: Robustness, Validity, Reliability

Overall the conclusions are robust, valid and justified. As mentioned above, a missing scATAC Seq might challenge the notion of involved transcription factors.
I have one major concern with the rationale from concluding of cell type specific expression towards the cell-autonomous role of the genes in the loss of function data. In particular, for the PLS3 gene, which was given as an example, the lack of clasical bone parameter differences for respective cell types does not mean that multiple cell types in bone are involved; it could also mean that none of the cell types are affected by PLS3 deficiciency and maybe a role in tissues systemically regulating bone mass (neuronal, endocrine system, skeletal muscle) might be more important.

F. Suggested improvements: experiments, data for possible revision

1. The study misses one great opportunity with this scRNASeq data set, which is revealing and validating cross-talk between the cell types which could explain phenotypes. Here many valuable tools are available (e.g. Cellphone, but more sophisticated Scriabin). These analysis might help to clarify some questions and riddles of genes functions either involved in multiple cell types, or in compartments like endothelial cells/VMSC directly impacting bone forming function by osteoblasts. These analyses would greatly improve the study.
2. Recently, multiomic analyses of human embryonic development had been published (https://www.nature.com/articles/s41586-024-08189-z). The comparison of human orthologue cell clusters there with the cell clusters observed in this study would be of great interest to see the translational potential and its limits in this approach.
3. In Figure 1 it is intriguing that mature neutrophils and CD14+ monocytes were detected in the endosteal compartment, but not in the bone marrow compartment. Is this a technical issue of neutrophil survival after prepration or a biological effect? Staining for CXCL2 + PMN and CD14+ cells could solve this problem.
4. The beauty of the study was that different bone regions were taken for analysis. I wonder whether there were exclusive clusters for either diaphysis or metaphysis. A metaphysial and diaphysial pseudotime analysis would provide very interesting information, since sufficient cells for analysis are available.
5. Technically, I am noticing that cells from bone marrow adipose tissue were not captured, is that due to preparative issues? This limitation of cell collection should be clearly mentioned as a limitation.
6. Endothelial cells and vascular cells exhibit a strong enrichment of genes associated with Bent bone dysplasia and genes associated with Protglycan core protein disorder (Fig. 2b), which is surprising. Can the authors draw a hypothesis from this, and are there functional data from the mouse knockout models that backup these data?
7. The authors claim that examples that are restricted for certain cell types such as Ptprd for osteoblasts. But Ptprd is highly enriched in different regions of the brain (Biogps.org and other resources). Therefore, it shows that a global knockout could hit other functions of other systemic acting organs (central nervous system, endocrine system, muscle, skin) that indirectly affect bone mass. Unfortunately, this possibility is largely ignored in the presentation of the results and in the discussion section. For drug use this could mean that side effects are still possible, or hitting organs outside the bone is essential to prevent skeletal diseases. The authors should, therefore, refine their rationale. In the ideal case, conditional cell lineage-restricted loss of function mouse models should elucidate this. This could be done for one or another example, but I agree that this is not suitable for large throughput.
8. In Fig. 5 the OBCD program should be better explained, what is precisely meant with structural, function, strucutral/function, quality only, Mahaionobis only phenotypes. The reference to literature is not sufficient.
9. In the method section, the versions of R packages should be explicitly mentioned.

G. References
The study clearly mentions previous work. The publication of the human bone cell atlas was published in November 2024, so it was not present during the submission. However, the data from this study should now be used for the revision (see above).
H. Clarity and context: lucidity of abstract/summary, appropriateness of abstract, introduction, and conclusions
This study is excellently and clearly written. Some conclusions should be revised (see above).

Reviewer #2 (Remarks to the Author):

The manuscript by Chai et al. combined scRNA-seq of C57BL/6J mice bone, multiscale functional enrichment analysis and gene knockout experiment in mice model to systematically prioritize effector genes related to skeletal diseases. Skeletal

supportive functional evidences include gene programs defined in non-haematopoietic cell types or osteoclasts, genes whose KO could result in abnormal skeletal phenotypes in mouse models (MGI or ORBD) as well as updated eBMD GWAS fine-mapping in UK Biobank. Although the manuscript provided some new resources or insights into skeletal diseases, most of the results are hypothetical which lacks deep mechanistical explorations. In addition, there are no evident technical breakthrough or conceptual innovation compared with other published human scRNA-seq or scATAC-seq studies. was My main comments are shown as follows:

1.Although the whole analyses are extensive and elaborate, most findings are collected from published data (MGI, ORBD), more in-depth experimental validation are needed to refine key therapeutic genes or pathways for skeletal diseases. In addition, several previous human scRNA-seq or scATAC-seq (Nature 2023/2024, Cell Research 2021) studies have been published. In contrast, no further technical breakthrough or conceptual innovation was accomplished in this paper (such as spatiotemporal omics or single-cell Hi-C). All analyses were based on predefined gene programs, which might have bias and lacks cross-sectional analysis compared to other public skeletal scRNA-seq or scATAC-seq data.

2.The gene program analysis in this paper was limited to non-haematopoietic cell types and osteoclasts, ignoring the potential important role of other cells in bone development. This work describes it as defining the entire cellular landscape of the endosteal bone compartment, where adipocytes are abundantly present in both bone marrow and bone tissue. However, despite this extensive exploration, no explicit description or definition of adipocytes was included in the analyses presented. Advancements now enable the extraction of individual adipocytes, including the ability to isolate the nucleus specifically to capture adipocytes.

3.The Pls3y gene is widely expressed in various cell types, such as chondrocyte, endothelial cell, vascular SMCs and osteoblast. Figure 6 displays the phenotypes of Pls3y knockout mice. However, the specific mechanism (pathway) by which this gene regulates its function in different cell types to affect bone phenotypes remains unclear, and further research is needed to elucidate the underlying mechanisms.

4.Long bones and calvarial bone have different developmental patterns, and the authors also need to consider whether there are differences in the specific cellular composition of their endosteal compartment.

5.On the whole, this paper is too long containing too many figures and descriptive/redundant results. It is highly suggested to simplify the topic and highlight the key points.

Version 1:

Decision Letter:

IMPORTANT: Please note the reference number: NG-A67136R-Z Kemp. This number must be quoted whenever you communicate with us regarding this paper.

3rd Jun 2025

Dear Dr. Kemp,

Thank you for your message asking us to reconsider our decision on your manuscript "Multiscale analysis and functional validation of the cellular and genetic determinants of skeletal disease". I have now discussed the points of your letter with my colleagues, and we think that you have some valid points. We therefore invite you to revise your manuscript along the lines that you propose.

When preparing a revision, please ensure that it fully complies with our editorial requirements for format and style; details can be found in the Guide to Authors on our website (http://www.nature.com/ng/).

Please be sure that your manuscript is accompanied by a separate letter detailing the changes you have made and your response to the points raised. At this stage we will need you to upload:
1) a copy of the manuscript in MS Word .docx format.
2) The Editorial Policy Checklist:
https://www.nature.com/documents/nr-editorial-policy-checklist.pdf
3) The Reporting Summary:
https://www.nature.com/documents/nr-reporting-summary.pdf
(Here you can read about the role of the Reporting Summary in reproducible science:
https://www.nature.com/news/announcement-towards-greater-reproducibility-for-life-sciences-research-in-nature-1.22062)

Please use the link below to be taken directly to the site and view and revise your manuscript:

Link Redacted

With kind wishes,
Chiara

Chiara Anania, PhD
Associate Editor
Nature Genetics
https://orcid.org/0000-0003-1549-4157

Version 2:

Decision Letter:

6th Nov 2025

Dear Dr Kemp,

Your Article, "Multiscale analysis and functional validation of the cellular and genetic determinants of skeletal disease" has now been seen by 2 referees. You will see from their comments below that while they find your work of interest, some important points are raised. We are interested in the possibility of publishing your study in Nature Genetics, but would like to consider your response to these concerns in the form of a revised manuscript before we make a final decision on publication.

To guide the scope of the revisions, the editors discuss the referee reports in detail within the team, including with the chief editor, with a view to identifying key priorities that should be addressed in revision and sometimes overruling referee requests that are deemed beyond the scope of the current study. We hope that you will find the prioritized set of referee points to be useful when revising your study. Please do not hesitate to get in touch if you would like to discuss these issues further.

We therefore invite you to revise your manuscript taking into account all reviewer and editor comments. Please highlight all changes in the manuscript text file. At this stage we will need you to upload a copy of the manuscript in MS Word .docx or similar editable format.

We are committed to providing a fair and constructive peer-review process. Do not hesitate to contact us if there are specific requests from the reviewers that you believe are technically impossible or unlikely to yield a meaningful outcome.

When revising your manuscript:

*1) Include a “Response to referees” document detailing, point-by-point, how you addressed each referee comment. If no action was taken to address a point, you must provide a compelling argument. This response will be sent back to the referees along with the revised manuscript.

*2) If you have not done so already please begin to revise your manuscript so that it conforms to our Article format instructions, available
here.
Refer also to any guidelines provided in this letter.

*3) Include a revised version of any required Reporting Summary: https://www.nature.com/documents/nr-reporting-summary.pdf
It will be available to referees (and, potentially, statisticians) to aid in their evaluation if the manuscript goes back for peer review.

A revised checklist is essential for re-review of the paper.

Please be aware of our guidelines on digital image standards.

EXTENDED DATA FIGURES

When re-submitting your manuscript, please ensure that any supplementary figures and tables that are crucial to the manuscript's conclusions are converted into Extended Data figures and tables to increase visibility of these data. Extended Data figures and tables are online-only (present in the online PDF and full-text HTML versions of the paper), peer-reviewed display items that provide essential background to the article but are not included in the main article due to space constraints. A maximum of ten Extended Data display items (figures and tables) is permitted.

Please use the link below to submit your revised manuscript and related files:

Link Redacted

Note: This URL links to your confidential home page and associated information about manuscripts you may have submitted, or that you are reviewing for us. If you wish to forward this email to co-authors, please delete the link to your homepage.

We hope to receive your revised manuscript within four to eight weeks. If you cannot send it within this time, please let us know.

Please do not hesitate to contact me if you have any questions or would like to discuss these revisions further.

Nature Genetics is committed to improving transparency in authorship. As part of our efforts in this direction, we are now requesting that all authors identified as 'corresponding author' on published papers create and link their Open Researcher and Contributor Identifier (ORCID) with their account on the Manuscript Tracking System (MTS), prior to acceptance. ORCID helps the scientific community achieve unambiguous attribution of all scholarly contributions. You can create and link your ORCID from the home page of the MTS by clicking on 'Modify my Springer Nature account'. For more information please visit please visit www.springernature.com/orcid.

We look forward to seeing the revised manuscript and thank you for the opportunity to review your work.

Sincerely,

Cong Zhao, PhD
Research Cross-Journal Editorial Team
Nature Genetics
https://orcid.org/0009-0008-5833-6662

Referee expertise:

Referee #1: bone biology, mouse model

Referee #2: genetics, osteoporosis

Reviewers' Comments:

Reviewer #1 (Remarks to the Author):

Overall I am impressed how the manuscript improved and the authors performed a tremendous effort to address the reviewer's comments and questions. The authors added much additional valuable information for the field including possible cell-cell type interaction data, snATAC Seq data and worked hard to find a mechanistic interpretation of specific loss of function models including introducing Zebra fish models in addition. The cross-talk of endothelial osteoblast interaction as a pivotal driver for endosteal homeostasis is intriguing and exciting. Integrating the human single cell RNASeq Data from a novel analysis is exciting as well.

I have therefore only minor remaining issues to be addressed and are rather related to the presentation of the data:

I appreciate the effort to perform snATAC Seq for the different cell types.
Here I think it is important to show the snATAC-Seq within the main figure and also take the top scoring TFs for interpretation as presentation into the results system, because this is likely the experimental evidence. Despite there is overlap it is

important to note that the major TF combination differ dramatically from the SCENIC prediction (TFAP2A, EBF2, KLF15 etc.) and preference should be given to these top scoring TFs also in the description of line 248 ff..

I was impressed by the introduction of data spatial omics of human bone into the paper in Extended Fig. 9. It would be easier to interprete the results also be displaying the H.E. histology of the cell type distributions.

Reviewer #2 (Remarks to the Author):

The authors have now provided new scRNA-seq, scATAC-seq and spatial-transcriptomic data to strengthen their conclusions, and have added experimental validation in mouse and zebrafish models. They also clarified how their work advances beyond previously published skeletal single-cell studies. Overall, the manuscript had been substantially improved. Nevertheless, several points still require clarification or revision before publication.
1. Pls3 is expressed in various bone cells such as osteoblasts, endothelial cells and VSMCs. The results showed that the deletion of Pls3 led to changes in bone vasculature and some bone phenotypes (Figure 5f-i), this is consistent with the strong expression of Pls3 in VSMCs and endothelial cells (Figure 5a), but it cannot lead to a rigorous conclusion that bone vascular changes are the cause of the bone phenotype in Pls3 knockout mice. It is recommended to use conditional knockout mice or other experiments for in-depth verification.

2. The author described in Line 149 that a new and now largest GWAS of eBMD was used, but when studying the mouse phenotype, other parameters excluding BMD of mouse lumbar vertebrae detected by Micro-CT were given in Fig5b. Was BMD affected? In the Figure 6, the BMD data of the Ptprd-/-, Cbx6-/-, Nherf2-/-, and Agap1-/- mice were not shown either? Why did the long bone BMD of Pls3 knockout mice not change compared with that of WT mice in Figure 5c?

3. Figure 6 shows Skeletal phenotypes of female Ptprd-/-, Cbx6-/-, Nherf2-/-, and Agap1-/- mice. Are there any differences in the mouse phenotypes between female and male? Were gender differences taken into account when analyzing the scRNA-seq data earlier?

Version 3:

Decision Letter:

Our ref: NG-A67136R2

1st Dec 2025

Dear Dr. Kemp,

Thank you for submitting your revised manuscript "Multiscale analysis and functional validation of the cellular and genetic determinants of skeletal disease" (NG-A67136R2). It has now been seen by the original referees and their comments are below. The reviewers find that the paper has improved in revision, and therefore we'll be happy in principle to publish it in Nature Genetics, pending minor revisions to satisfy the referees' final requests and to comply with our editorial and formatting guidelines.

If the current version of your manuscript is in a PDF format, please email us a copy of the file in an editable format (Microsoft Word or LaTex)-- we can not proceed with PDFs at this stage.

We are now performing detailed checks on your paper and will send you a checklist detailing our editorial and formatting requirements soon. Please do not upload the final materials and make any revisions until you receive this additional information from us.

Thank you again for your interest in Nature Genetics Please do not hesitate to contact me if you have any questions.

Sincerely,

Cong Zhao, PhD
Research Cross-Journal Editorial Team
Nature Genetics
https://orcid.org/0009-0008-5833-6662

Editorial comments:

Further revisions include:
Author list: Corresponding author needs an email on the title page. For the IFMRS Big Data Working Group, please assign the consortium authors numbered affiliations that are continuous with the numbering of the affiliations from the main author list (rather than starting over at 1).

Word counts: Ensure main text, results subheadings, methods and figure legends comply with NG's standards (Main text~4,000 words; Results subheadings~59 words; Methods ~3,000 words; Main Figure legends & Extended Data Figure legends & Supplementary Figure legends ~250 words)
References: Split them for main text and for methods.
Data availability & code availability: All codes, scRNA-seq raw data, snATAC-seq raw data, GWAS summary result statistics and other statistic data should be released immediately. For code availability, please deposit the code in a DOI-minting repository (in addition to GitHub).
Supplementary information: Upload Supplementary table 2-9 as one excel file (one Supplementary Table with multiple tabs); move all primer/crRNA sequences into one tab within Supplementary Table; shorten Extended Data Figure legends & Supplementary Figure legends; move excess texts of methods to Supplementary Note; Supplementary Figures and Supplementary table 1 should be combined into 1 PDF
Define all elements in legend and text (abbreviations, acronyms, colors, symbols, lines etc.). Check gene/protein nomenclature in all figures and legends. Check all graphs have clearly labelled axes. Check that all legends have a title and that all figure panels have a legend. Check that all display items are cited correctly in the text (in the right order). Check and confirm statistics of figures and raw data.

Reviewer #1 (Remarks to the Author):

The authors addressed all my concerns and I congratulate them to this important and exciting study.

Reviewer #2 (Remarks to the Author):

Although this study lacked direct evidence from conditional mouse models due to time constraints and missed some phenotypic measurements owing to technical limitations, the robustness of the data and the extent of the experimental work overall support the main conclusions. Therefore, I recommend acceptance for publication.

Open Access This Peer Review File is licensed under a Creative Commons Attribution 4.0 International License, which permits use, sharing, adaptation, distribution and reproduction in any medium or format, as long as you give appropriate credit to the original author(s) and the source, provide a link to the Creative Commons license, and indicate if changes were made.
In cases where reviewers are anonymous, credit should be given to 'Anonymous Referee' and the source.
The images or other third party material in this Peer Review File are included in the article's Creative Commons license, unless indicated otherwise in a credit line to the material. If material is not included in the article's Creative Commons license and your intended use is not permitted by statutory regulation or exceeds the permitted use, you will need to obtain permission directly from the copyright holder.
To view a copy of this license, visit https://creativecommons.org/licenses/by/4.0/

Point-by-Point Response to Reviewer's Comment on the manuscript entitled 'Multiscale analysis and functional validation of the cellular and genetic determinants of skeletal disease' (NG-A67136R-Z Kemp).

Detailed responses to the editorial and reviewer's comments are highlighted in blue and proposed changes to the manuscript in *red*.

Response to Editorial Comments

We appreciate your thoughtful suggestions about the key points to address:

1. Reviewer #1 appreciates the resource value but highlights some limitations of the study, i.e., the lack of a scATAC-seq dataset, and the missed opportunity to analyze adipose tissues and cell-cell communication. They are also concerned that two replicates for the mouse work are not sufficient to make strong claims.

We have now included a series of new datasets, including a new murine snATAC-seq dataset to support our transcription factor analysis, a new human scRNA-seq dataset to validate our murine data and illustrate the translational potential and spatial transcriptomic data to analyse bone marrow adipocytes. We have also included an analysis of cell-cell communication and provided a clear explanation and evidence of the statistical robustness of the mouse analyses that support our conclusions.

2. Reviewer #2 acknowledges that the study provides some new resources and insights into skeletal diseases, but they think most results would require stronger mechanistic explorations. They also believe you should include adipocytes in your analysis.

We have now included data on bone marrow adipocytes. We have also included further mechanistic insights, including data showing that vascular changes account for bone phenotype in *Pls3* knockout mice. We also validated these findings further by showing that deletion of *pls3* in zebrafish causes an abnormal vascular phenotype.

Further details on these experiments and the approaches to address the individual comments raised by the reviewers are detailed below:

Response to Reviewers' Comments:

Response to Reviewer 1:

We appreciate the reviewer's careful comments and the view that *"Overall the conclusions are robust, valid and justified.... "* and *"..this study is excellently and clearly written ….."* . We were also pleased to note the reviewer had accessed the dataset, performed their own partial analysis and showed similar results, highlighting the quality of the data and the robustness of the analytical approach. Responses to specific comments are detailed below:

C. Data & methodology: validity of approach, quality of data, quality of presentation

The suggestions of involved transcription factors by the Scenic algorithm is an approximation but would benefit from a scATAC Seq for validity. Therefore, the data should be presented as more suggestive as currently stated.

We agree the SCENIC algorithm is an approximation. We have now generated a new snATAC-seq dataset and completed an analysis of these data. This has enabled cross-validation of the outcomes of the SCENIC analyses. Modifications to the manuscript include:

a. *Refinements to the interpretation of the SCENIC analysis in the Results section (page 7, line 248).*
b. *A description of the snATAC-seq method and analytical approach in the Methods section (page 24, line 804).*
c. *Inclusion of the snATAC-seq analysis in the Results section and in Supplementary Figure 3 (page 8, line 252).*

D. Appropriate use of statistics and treatment of uncertainties

A challenge is the low number of mutant mice used in the assessment of bone parameters. While this is intrinsic by testing so many mutants, n = 2, despite having a large n for wt mice, this remains to be improved and the data taken with caution.

We performed detailed structural and functional bone phenotype analysis of >1000 unselected knockout (KO) mouse lines as part of the Origins of Bone and Cartilage Disease (OBCD) program. This required development of a pipeline to identify robust phenotypes using minimal numbers of mice. Phenotyping was performed on 2-6 mice from *each* KO line and compared to a cohort of *320* wildtype littermates generated throughout the duration of the study. KO mouse lines with an average or median skeletal parameter >2 standard deviations from the mean or outside the 2.5–97.5th percentiles of the normal wildtype reference range were considered outliers and to have an abnormal bone phenotype. Outlier parameters were independently identified by permutation testing. This calculated the chance of selecting an equal number of WT samples, with an average or median parameter equal to or more divergent than that observed in the KO line. A significance threshold of $P<4.37\times10^{-3}$ was determined after correction for multiple testing. So far eleven KO lines identified by the OBCD screen with outlier skeletal phenotypes (ten identified using n=2 mice and one using n=6 mice) have been rederived as independent mouse colonies and all have been shown to have concordant abnormal skeletal phenotypes. Four of these have already been published (*Gpc6* – Kemp *et al*., *Nature Genetics* 2017, doi.org/10.1038/ng.3949, *Daam2* - Morris *et al*., *Nature Genetics* 2019, doi.org/10.1038/s41588-018-0302, *Cadm1* - Nakamura *et al*., *Plos One* 2017, doi.org/10.1371/journal.pone.0175632 & *Sparc* - Mansergh *et al*., *Physiological Genomics* 2007, doi.10.1152/physiolgenomics.00151.2007). *Pls3* is included in this manuscript and six are currently unpublished. We have modified the manuscript to highlight this new information and include appropriate caution. Modifications include:

a. *Explanation in the Methods section to better articulate the statistical approach (page 40, line 1340; page 41, line 1365).*
b. *Amending the Discussion section to highlight limitations of the study (page 18, line 596).*

E. Conclusions: Robustness, Validity, Reliability

Overall the conclusions are robust, valid and justified. As mentioned above, a missing scATAC Seq might challenge the notion of involved transcription factors. I have one major concern with the rationale from concluding of cell type specific expression towards the cell-autonomous role of the genes in the loss of function data. In particular, for the PLS3 gene, which was given as an example, the lack of clasical bone parameter differences for respective cell types does not mean that multiple cell types in bone are involved; it could also mean that none of the cell types are affected by PLS3 deficiciency and maybe a role in tissues systemically regulating bone mass (neuronal, endocrine system, skeletal muscle) might be more important.

We have generated new snATAC-seq data to investigate whether the transcription factors we identified in the SCENIC analysis regulate cell type specific gene programs (see point C above).

We also agree with the reviewer that even if a gene is restricted in expression to a single cell type in bone, we cannot exclude a functional role being mediated in a systemic manner via expression in an extra-skeletal tissue. To acknowledge this, we reported the extra-skeletal tissues in mice that express the exemplar genes in Supplementary Figure 6 and Supplementary Table 9. However, we appreciate that the implications were not addressed clearly and have modified the text to address this. We have also included expression of exemplar genes in extra-skeletal tissues in humans in a modification to Supplementary Figure 6 and Supplementary Table 9.

In the case of *Pls3* deletion, despite a robust skeletal phenotype there were no differences in bone resorption or formation parameters. Given the expression was highest in vascular smooth muscle cells

(VSMCs) and endothelial cells, we have undertaken further analyses, including developing new micro-CT methods, to examine the skeletal vasculature in *Pls*$^{y/-}$ mice. This analysis identified significant abnormalities in the cortical bone vasculature. To investigate this further we developed a new model of *pls3* deletion in zebrafish and showed deletion caused vascular abnormalities. This is consistent with the role for *PLS3* that has been proposed in human vascular disease (Pan *et al*., 2020, *Arteriosclerosis Thrombosis & Vascular Biology* 2020, DOI: 10.1161/ATVBAHA.119.313440). We would agree with the reviewer that the effect of *Pls3* deletion on the skeleton could still result from its actions in extra-skeletal tissues that systemically regulate bone mass. We have included this new mechanistic data, including the data from zebrafish and commented on the potential of local and systemic regulation of bone in the Discussion section. Modifications to the manuscript include:

a. *Alterations to the Methods section to describe the snATAC-seq method (page 24, line 804).*
b. *snATAC-seq data in the Results section and in Supplementary Figure 3 (page 8, line 252).*
c. *A revised Supplementary Figure 6 and Supplementary Table 9 to include extra-skeletal expression of exemplar genes in both mouse and human tissues.*
d. *New data in the Results section on the impact of Pls3 deletion on the bone vasculature in mice and vasculature in zebrafish and inclusion of data in Figure 5 and Supplementary Figure 9 (page 14, line 467).*
e. *Modifications to the Discussion section to comment on local vs systemic control of bone (page 17, line 580).*

F. Suggested experiments

1. The study misses one great opportunity with this scRNASeq data set, which is revealing and validating cross-talk between the cell types which could explain phenotypes. Here many valuable tools are available (e.g. Cellphone, but more sophisticated Scriabin). These analysis might help to clarify some questions and riddles of genes functions either involved in multiple cell types, or in compartments like endothelial cells/VMSC directly impacting bone forming function by osteoblasts. These analyses would greatly improve the study.

We thank the reviewer for this suggestion. We have now undertaken ligand/receptor analysis, using the CellTalkDB database (Shao et al., *Briefings in Bioinformatics* 2021, doi.org/10.1093/bib/bbaa269) and the Cellphone tool, to examine the cross talk between cell types. Our analysis of the entire dataset shows the some of the strongest interactions occur between cells of the osteoblast lineage and endothelial cells and VSMCs. Further interrogation of the data also identified many of the key molecular pathways predicted to mediate interactions between these cell types. Together this supports the suggestion that endothelial cells and VSMCs can interact directly with cells of the osteoblast lineage to regulate bone formation in the endosteal compartment. As suggested by the reviewer this may help explain how genes that are expressed in endothelial cells and VSMCs, including *Pls3*, *Nherf2* and *Cbx6* can regulate bone mass. We agree with the reviewer that this new data strengthens the manuscript significantly and have include this in the Methods, Results and Discussion sections. Revisions to the manuscript include:

a. *Ligand/receptor cross talk analysis in Figure 1 and a new Extended Data Figure 2.*
b. *Modifications to the Methods (page 23, line 757), Results (page 7, line 218) and Discussion (page 17, line 582) sections to describe the findings and discuss the implications.*

2. Recently, multiomic analyses of human embryonic development had been published (https://www.nature.com/articles/s41586-024-08189-z). The comparison of human orthologue cell clusters there with the cell clusters observed in this study would be of great interest to see the translational potential and its limits in this approach.

We agree and have generated a new dataset of cells isolated from adult human femoral bone. We have compared this with our mouse data and demonstrate remarkable similarities. The major cell types, including all bone cells, endothelial cells and VSMC and their gene programs are conserved between mouse and adult human samples. Furthermore, enrichment analysis shows the same cell clusters are

enriched with genes that cause rare skeletal diseases and/or are associated with eBMD in both murine and human datasets highlighting the translational potential of our approach.

As suggested by the reviewer we have also analysed the human embryonic joint and cranium development dataset (To et al., *Nature*, 2024, doi.org/10.1038/s41586-024-08189-z). This dataset contained >45 clusters of non-haemopoietic cells reflecting the complex and specialised processes that occur during joint and cranial development. Comparison of this dataset with our endosteal bone dataset showed that some cell populations, including osteoblasts and osteocytes had similar gene programs and were enriched with genes associated with eBMD. However other cell types, including endothelial cells, VSMCs and cells from earlier stages in the osteoblast lineage had gene programs that differed between datasets and were not enriched with genes associated with eBMD. This may be due to differences in tissue type, the cranium forming through intramembranous ossification, whereas the two articulated bones in the joint forming through endochondral ossification. Alternatively, this may reflect tissues sampled from different locations or developmental stage. This highlights the importance of considering tissue location and age when analysing single cells.

We included the new adult human data set and a detailed comparison with the murine data in the revised manuscript. We have also highlighted the comparison with the published human embryonic development dataset as in the results section. Revisions to the manuscript include:

a. *Comparison of the murine data with the new adult human dataset in the Results section, in Figure 7 and Supplementary Table 2 (page 15, line 509).*
b. *Enrichment analysis for genes that cause rare skeletal disorders and/or are associated with eBMD in adult human cells in the Results section and Figure 7 and Supplementary Tables 4 and 5 (page 15, line 518).*
c. *Commentary on our unpublished comparison of recent embryonic bone data with our new adult bone data in the Results section (page 15, line 521).*
d. *Commentary on areas of consistency between murine and human data in Results (page 15, line 512), and limitations comparing different tissue types and anatomical sites in Discussion (page 18, lines 596 and 606).*

3. In Figure 1 it is intriguing that mature neutrophils and CD14+ monocytes were detected in the endosteal compartment, but not in the bone marrow compartment. Is this a technical issue of neutrophil survival after prepration or a biological effect? Staining for CXCL2 + PMN and CD14+ cells could solve this problem.

We agree this is intriguing. Our single cell RNA-seq data show these cells were present in the bone marrow compartment, although they were highly enriched in the endosteal compartment. As suggested by the reviewer we have now performed a flow cytometric analysis. This confirmed that CD14+ neutrophils and monocytes are present in both compartments and there was a significant increase in CD14+ neutrophils and monocytes in the endosteal compartment when compared to the marrow compartment. This suggests the endosteal compartment is distinct from the marrow compartment. This may reflect the different biological activities that occur in these different locations. We have included this new data in the Results and Discussion sections. Modification to the revised manuscript include:

a. *New flow cytometric data showing enrichment of CD14+ neutrophil and monocyte populations in the endosteal compartment relative to bone marrow compartments in Supplementary Figure 1.*
b. *Modifications to the Results section (page 5, line 181) to describe FACS data and Discussion section to highlight the specialised location of the endosteal compartment in bone (page 17, line 567).*

4. The beauty of the study was that different bone regions were taken for analysis. I wonder whether there were exclusive clusters for either diaphysis or metaphysis. A metaphysial and diaphysial pseudotime analysis would provide very interesting information, since sufficient cells for analysis are available.

The two endosteal compartments of the diaphysis and metaphysis were similar. The only exclusive cluster was chondrocytes which were present in the metaphysis but not the diaphysis. There were also significantly more pre-osteoblasts in the diaphysis than the metaphysis. A trajectory analysis (pseudotime analysis) of cells of the osteogenic lineage from the two endosteal compartments showed the transition of cells from mesenchymal stromal cells through to osteoprogenitors, pre-osteoblasts, mature osteoblasts and then early osteocytes in both the diaphysis and metaphysis. In the metaphysis, but not diaphysis, cells branch to form resting, pre-hypertrophic and hypertrophic chondrocytes. We have included this analysis in the revised manuscript. Modification to the manuscript include:

a. *Alterations to the Results section and inclusion of a new Supplementary Figure 2 to illustrate that chondrocytes are found exclusively in the endosteal metaphyseal compartment (page 8, line 263).*
b. *Refinements to the Results section and Supplementary Figure 2 to show that pre-osteoblasts are enriched in the endosteal diaphyseal compartment (page 7, line 241).*
c. *Trajectory analysis of the two endosteal compartments in Extended Data Fig 3i.*
d. *Modification to the Discussion section to refer to the differences in cell populations between the different endosteal compartments (page 17, line 567).*

5. Technically, I am noticing that cells from bone marrow adipose tissue were not captured, is that due to preparative issues? This limitation of cell collection should be clearly mentioned as a limitation.

Bone marrow adipocytes were not captured in our dataset for two reasons. First, adipocytes are often not present in significant numbers in the long bones of younger animals (Tratwal *et al., Frontiers Endocrinology* 2020, 11, 480, doi: 10.3389/fendo.2020.00480). We confirmed this in the present study by histological analysis of sections of the femur of 9-10-week-old mice. We performed the same analysis in 26- and 68-week-old mice. This showed few adipocytes in the endosteal and bone marrow compartments at all ages. This was confirmed further using osmium tetroxide staining and micro-CT analysis of mice of different ages (unpublished data). Secondly, the size and fragility of adipocytes make them difficult to isolate for scRNA-seq analysis. The dataset does contain populations of cells that express *Adipoq* (Adiponectin), *Apoe* (Apolipoprotein E) and *Lpl* (Lipoprotein lipase) suggesting that adipocyte precursors are present. We also performed single nucleus-RNA-seq (snRNA-seq) analysis of 26- and 68- week-old mice (Fig 1. below), which confirmed the histological analysis.

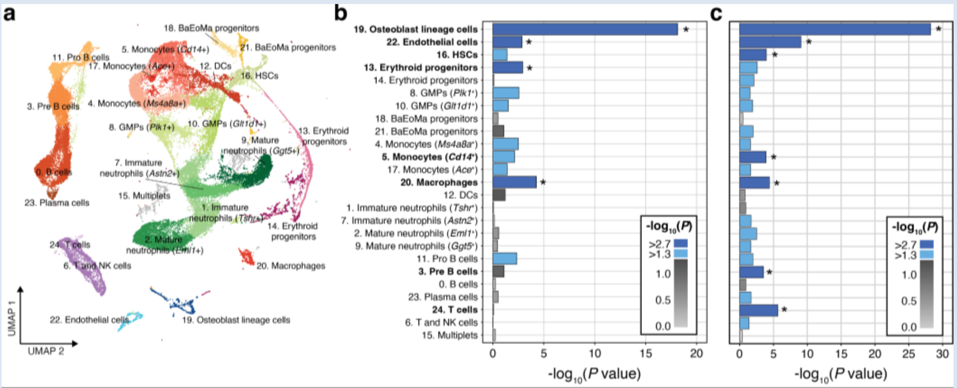

Fig 1. (a) UMAP of snRNA-seq data from nuclei of cells isolated from the endosteal compartment from mouse bone showing 25 cell clusters. Bar plots show enrichment of (b) monogenic skeletal disorder causative genes and (c) eBMD-associated genes in gene programs of endosteal cell types identified by snRNA-seq.

To address these limitations, we performed a spatial transcriptomic analysis of human bone samples. This identified a distinct population of adipocytes. Analysis of these cells showed they were not enriched with genes that cause rare skeletal disorders or are associated with eBMD. We have included the analysis of this spatial transcriptomic data in the revised manuscript. Analysis of the snRNA-seq data showed strong concordance with our scRNA-seq data. Furthermore, enrichment analysis confirmed that nuclei from cells

of the osteoblast lineage and endothelial cells were enriched with genes that cause monogenic skeletal disorders and are associated with eBMD (Fig. 1). Given the concordance and to avoid duplication we have shared the data with the reviewers but have not included this data in the revised manuscript. Modification to the manuscript include

a. *Inclusion of the adipocyte data in the Results (page 16, lines 531 and 536), Discussion (page 18, line 603) sections and in Extended Data Figure 9.*
b. *Commentary on technical limitations in the Discussion sections (page 18, line 598).*

6. Endothelial cells and vascular cells exhibit a strong enrichment of genes associated with Bent bone dysplasia and genes associated with Protglycan core protein disorder (Fig. 2b), which is surprising. Can the authors draw a hypothesis from this, and are there functional data from the mouse knockout models that backup these data?

We agree this was an interesting observation. Detailed analysis has suggested this enrichment may be due to causal genes for these disorders being shared across cell types. For example, the genes that encode biglycan (*Bgn*) and perlecan (*Hspg2*), mutations in which cause proteoglycan core protein disorders, are expressed in endothelial cells, VSMCs, osteoblasts and chondrocytes (see manuscript Fig 2). The skeletal phenotypes in these disorders could be mediated via these cell types. Analysis of mice with deletions of these genes show they also have extra-skeletal phenotypes. For example, mice with deletions in *Hspg2* have vascular abnormalities (Chang *et al* Cardiovascular Research **107**(1): 20-31). This would support the hypothesis that expression of these disease-causing genes in endothelial and VSMCs may result in vascular changes that could contribute to the phenotype in bone. We have now commented on these possibilities. Modification to the manuscript include:

a. *Highlighting the enrichment of genes associated with these disorders in endothelial and VSMC in the Results section (page 9, line 305).*
b. *Commenting on the potential of shared genes accounting for enrichment in the Results section (page 9, line 308).*
c. *Commentary on the impact of shared genes in enrichment analysis in the discussion (page 18, line 610).*

7. The authors claim that examples that are restricted for certain cell types such as Ptprd for osteoblasts. But Ptprd is highly enriched in different regions of the brain (Biogps.org and other resources). Therefore, it shows that a global knockout could hit other functions of other systemic acting organs (central nervous system, endocrine system, muscle, skin) that indirectly affect bone nervous system, endocrine system, muscle, skin) that indirectly affect bone mass. Unfortunately, this possibility is largely ignored in the presentation of the results and in the discussion section. For drug use this could mean that side effects are still possible, or hitting organs outside the bone is essential to prevent skeletal diseases. The authors should, therefore, refine their rationale. In the ideal case, conditional cell lineage-restricted loss of function mouse models should elucidate this. This could be done for one or another example, but I agree that this is not suitable for large throughput.

We agree with the reviewer. The genes we selected as exemplars were restricted to specific cell types in bone; however, we acknowledge they may also be expressed in cells outside of bone. For each exemplar gene we did show the non-skeletal cells/tissues that express these genes in Supplementary Figure 6 and Supplementary Table 9. Nevertheless, we appreciate these data and their implications were not discussed in detail. We have extended this analysis to include expression patterns of both murine and human exemplar genes.

We also agree that lineage restricted models would be useful, however, these lines are not suitable for the large throughput analysis undertaken in this study. Generating further new KO lines at the scale required would take a considerable time and is beyond the scope of this paper. We propose to acknowledge this and include this in the limitation section in the Discussion section. Modification to the manuscript include:

a. *Reference to expression of exemplar genes in cells outside of bone in the Results section (page 14, line 491).*
b. *Alterations to Supplementary Figure 6 to include expression of the human genes in tissues outside of bone, including PTPRD expression in brain.*
c. *Changes to the Discussion section to discuss the implication of expression of genes outside of bone on the mechanism of action in (page 17, line 580).*
d. *Acknowledging the value of conditional cell lineage restricted models in future studies in the Discussion section (page 18, line 618).*

8. *In Fig. 5 the OBCD program should be better explained, what is precisely meant with structural, function, structral/function, quality only, Mahaionobis only phenotypes. The reference to literature is not sufficient.*

We appreciate the OBCD program needs clearer explanation. We also appreciate the precise meaning of the bone phenotypes need to be clearly articulated when first identified in the Results section. This includes mouse lines with:

- *Structural phenotypes* – Significant difference in bone structural parameters measured by digital X-ray microradiography or micro-computerised tomography.
- *Functional phenotypes* –Significant differences in mechanical strength parameters measured by biomechanical testing
- *Structural and functional phenotypes* – Significant differences in at least one structural and one functional parameter
- *Bone quality phenotypes* – lines with outlier functional phenotypes that did not correlate with bone mineral content
- *Mahalanobis phenotypes* – lines that were significant outliers due to smaller differences in multiple skeletal parameters.

We have included clearer explanations of the different phenotypes in Supplementary Table 1 and Methods section and when first used in the Results section. Modification to the manuscript include:

a. *Changes to the Supplementary Table 1 and Methods section to improve explanation of the OBCD program and phenotyping pipeline and the precise meaning of the different phenotypes (page 35, line 1163).*
b. *Clarification in the Results section to explain the different bone phenotypes when first mentioned (page 12, line 410).*

9. *In the method section, the versions of R packages should be explicitly mentioned.*

We agree this is important. Versions were included in the *Nature Genetics* submission form but were not always included in the Methods section. We have ensured versions of all software packages are included in the Methods section. Modification to the manuscript include:

a. *Addition of versions of each software package used in the Methods section.*

G. References

The study clearly mentions previous work. The publication of the human bone cell atlas was published in November 2024, so it was not present during the submission. However, the data from this study should now be used for the revision (see above).

We thank the reviewer for highlighting this opportunity to update our results in light of To *et al., Nature* 2024 (doi.org/10.1038/s41586-024-08189-z), which was published after our submission. Whilst the data is generated from human embryonic joint and cranium development rather than adult bone we agree it would be important to include in a revised manuscript. As outlined under point 2 above, we have included reference to our new adult data and compared this with data generated during embryonic development. Modification to the manuscript include:

a. *Inclusion of the new adult human dataset and comparison with the mouse data in the Results section, in Figure 7 and Supplementary Table 2 (page 15, line 509).*

b. *Amending the Results and Discussion sections to include commentary on areas of consistency between murine and human data (page 15, lines 512, 518) and limitations comparing different tissue types and anatomical sites (page 18, line 596).*

H. Clarity and context: lucidity of abstract/summary, appropriateness of abstract, introduction, and conclusions

This study is excellently and clearly written. Some conclusions should be revised (see above).

As outlined above we have modified the Result and Discussion sections to address the comments raised and revise the conclusions. Modification to the manuscript include:

a. *Alterations to the Results and Discussion sections to revise our conclusions in line with the responses to the reviewer's comments.*

Response to Reviewer 2:

We appreciate the reviewer's thoughtful comments and the recognition that the analyses are "extensive and elaborate". Responses to specific comments are detailed below:

1. Although the whole analyses are extensive and elaborate, most findings are collected from published data (MGI, ORBD), more in-depth experimental validation are needed to refine key therapeutic genes or pathways for skeletal diseases. In addition, several previous human scRNA-seq or scATAC-seq (Nature 2023/2024, Cell Research 2021) studies have been published. In contrast, no further technical breakthrough or conceptual innovation was accomplished in this paper (such as spatiotemporal omics or single-cell Hi-C). All analyses were based on predefined gene programs, which might have bias and lacks cross-sectional analysis compared to other public skeletal scRNA-seq or scATAC-seq data.

The studies described in the present manuscript were built on novel single cell datasets generated 'in house' mapping different bone compartments in mice, and a new expanded analysis of UK Biobank data which includes >21K more individuals and >10 million more genetic variants than previously analysed. We utilised the publicly available MGI database to support high level experimental validation of gene programs only. This was complemented with a new analysis of >1000 unselected mouse lines with single gene deletions from our OBCD program. This is a significant extension to the original report of 100 lines (Bassett *et al*., *Plos Genetics* 2012, journal.pgen.1002858) and the 596 lines reported in 2019 (Morris *et al*., *Nature Genetics* 2019, doi.org/10.1038/s41588-018-0302). This new analysis enabled the functional validation of 43 genes that were restricted in expression to the gene programs of cells associated with skeletal disease. We appreciate that the novelty of some of these datasets, our approach of integrating the analysis of these datasets and the link to experimental validation may not have been clear in the manuscript. We have clarified this in the revised manuscript.

We also acknowledge that human datasets have recently been reported. The datasets highlighted by the reviewer were generated from tissues taken from limb buds, joints and cranium during embryonic development rather than adult human bone (To et al., *Nature* 2024, doi.org/10.1038/s41586-024-08189-z & He et al., *Cell Research* 2021, doi.org/10.1038/s41422-021-00467-z). This makes comparison with cells isolated from the endosteal bone compartment in the current study and commentary on adult bone homeostasis and disease difficult. To address this challenge, we have generated a new human scRNA-seq dataset from adult human femoral bone to enable more appropriate cross-sectional comparison. This analysis shows strong similarities in the cell types and gene programs that control cells in bone in both mice and humans. This has also revealed strong enrichment for genes associated with eBMD in the same cell types in both mice and humans. This cross-sectional and orthogonal analyses support our approach and our conclusions. The cross-species conservation also enables future detailed studies in mice that are not possible in humans and helps further define the genes that are most relevant to human skeletal health. We have included this new human scRNA-seq dataset and a comparison with the murine data in the revised manuscript. We have also commented on comparisons to the embryonic datasets highlighted by the reviewer.

We thank the reviewer for suggesting the inclusion of new approaches. In parallel with the adult human dataset outlined above, we have included a new snATAC-seq dataset from murine bone to validate our our transcription factor analysis, and spatial transcriptomic data to analyse bone marrow adipocytes in humans. The inclusion of these new datasets and analyses has further strengthen the findings of the manuscript. We have also undertaken a snRNA-seq analysis, which further validated our enrichment analysis of our scRNA-seq data. The reviewer has highlighted the importance of focusing the manuscript, so we have not included the snRNA-seq data in the revised paper. However, we have shared this with reviewers (see reviewer 1, point 5).

Lastly the reviewer noted that there may be biases in our approach. We agree that with all experimental systems there are potential biases. To mitigate this we have integrated independent, complementary approaches throughout our analysis. This now includes: 1. Using agnostic approaches to identify cell populations in our datasets; 2. Utilising adult human datasets to validate findings generated in our murine analysis; 3. Using different methodologies to cross validate findings, e.g. flow cytometric analysis to confirm scRNA-seq findings, micro-CT methods to examine the effects of gene deletion on bone vasculature, and a new zebrafish model to validate vascular phenotypes; 4. Undertaking functional analysis of exemplar genes selected from gene programs identified through enrichment analysis. Together these orthogonal analyses show compelling similarities across datasets and across species. Coupling this with detailed functional analysis increases the confidence in the conclusions drawn.

We recognise the importance of articulating this approach and acknowledging there are limitations. We have modified the manuscript to ensure this is made clear throughout and to better articulate the limitations in the Discussion section. Modification to the manuscript include:

a. *Clarification of which datasets are novel throughout the Methods and Results sections.*
b. *New adult human scRNA-seq data in Figure 7 and Supplementary Tables 2, 4 and 5, and changes to the Methods (page 19, line 651) and Results (page 15, line 509) sections to describe this data.*
c. *Reference to the embryonic datasets in the Result section (page 15, line 521).*
d. *New snATAC-seq data in Supplementary Figure 3 and Supplementary Table 4, and changes to the Methods (page 24, line 804) and Results (page 8, line 252) sections to describe this data.*
e. *Modifications to the Discussion sections to highlight potential limitations and approaches to mitigation (page 18, line 596).*

2. The gene program analysis in this paper was limited to non-haematopoietic cell types and osteoclasts, ignoring the potential important role of other cells in bone development. This work describes it as defining the entire cellular landscape of the endosteal bone compartment, where adipocytes are abundantly present in both bone marrow and bone tissue. However, despite this extensive exploration, no explicit description or definition of adipocytes was included in the analyses presented. Advancements now enable the extraction of individual adipocytes, including the ability to isolate the nucleus specifically to capture adipocytes.

The aim of this study was to identify the cell types and genes that regulate bone. An important goal is to help identify and prioritise genes for future study as therapeutic targets in human disease. Of the 34 cell types analysed, non-haemopoietic cells, including cells of the osteoblast lineage, chondrocytes, endothelial cells and VSMCs, and osteoclast were those most strongly enriched with genes associated with skeletal disorders suggesting that many of the genes that control their function are likely to regulate the skeleton. Analysis of our new human dataset confirmed the enrichment of genes associated with skeletal diseases in non-haematopoietic cells. For this reason, we prioritised genes expressed in these cell types for functional validation.

We appreciate the reviewer's comments about adipocytes. These cells were not present in our analysis for several reasons. Adipocytes are not present in significant numbers in younger animals (Tratwal *et al*., Frontiers Endocrinology 2020, 11, 480, doi: 10.3389/fendo.2020.00480). We confirmed this by histological analysis of sections of the femur of mice. We also confirmed that adipocytes were rare even

in 68-week-old mice of this strain. Secondly, the size and fragility of adipocytes make them difficult to isolate for scRNA-seq analysis, suggesting alternative techniques are required. As suggested by the reviewer we performed snRNA-seq analysis of cells from bone marrow and endosteal compartments of mice at different ages. This analysis confirmed the absence of adipocytes. However, we have performed a spatial transcriptomic analysis of human bone biopsies. This identified a population of adipocytes. Unlike osteoblast, endothelial cells and VSMCs, the adipocyte gene program was not enriched with genes that cause rare skeletal disease or are associated with eBMD. We have included this spatial transcriptomic analysis in the revised manuscript. Modification to the manuscript include:

a. *Clarification of the rationale behind the gene programs selected for detailed functional analysis in the Results section (page 11, line 384).*
b. *Inclusion of the spatial transcriptomic analysis of human bone biopsies the Results section (page 16, line 529).*

3. The Pls3y gene is widely expressed in various cell types, such as chondrocyte, endothelial cell, vascular SMCs and osteoblast. Figure 6 displays the phenotypes of Pls3y knockout mice. However, the specific mechanism (pathway) by which this gene regulates its function in different cell types to affect bone phenotypes remains unclear, and further research is needed to elucidate the underlying mechanisms.

We agree with the reviewer. *Pls3* was expressed in multiple cells in bone, including osteoblasts, endothelial cells and VSMC. Analysis of *Pls*$^{y/-}$ mice showed they have a robust skeletal phenotype, but the phenotype was not mediated by changes in bone formation or bone resorption. We have therefore undertaken further detailed analysis of the cellular mechanisms. This has shown that deletion of *Pls3* is associated with changes in bone vasculature (Fig. 5f-i) which is consistent with strong expression of *Pls3* in VSMCs and endothelial cells (Fig.5a in revised manuscript). We also confirmed this vascular phenotype in a new zebrafish model of *pls3* deletion. This finding provides a potential cellular mechanism to explain the reduced bone mass and strength following *Pls3* deletion (see reviewer 1, point E for additional details). We agree that further research in future studies is required to identify the precise molecular pathways in VSMCs and endothelial cells responsible for this effect. We have included this new mechanistic data in the revised manuscript. Modification to the manuscript include:

a. *New data on the impact of Pls3 gene deletion on the vasculature in the Results section, Figure 5f-i and Supplementary Figure 9 (page 14, line 467).*
b. *Commentary on the cellular mechanisms accounting for the bone phenotype in the Discussion sections (page 17, line 580).*

4. Long bones and calvarial bone have different developmental patterns, and the authors also need to consider whether there are differences in the specific cellular composition of their endosteal compartment.

The reviewer has raised an interesting point. Analysis of the recently reported human embryonic joint and cranium development dataset has shown differences in cellular composition between bone types (To et al., *Nature* 2024, doi.org/10.1038/s41586-024-08189-z). Studies of osteoblastic cells isolated from calvaria and long bones have shown they express similar gene programs (Ayturk et al., *Journal of Bone & Mineral Research* 2020, doi: 10.1002/jbmr.4052). We have shown that gene programs of osteocytes isolated from different bones are also remarkably similar, although there are differences in some specific gene programs in cells isolated from different bones (Youlten et al., *Nature Communications* 2021, doi.org/10.1038/s41467-021-22517-1). This raises the possibility that the cellular composition of the endosteal compartment may differ between bone types and for some cell types, specific gene programs may also differ. This is an important idea. We have modified the Discussion section to highlight this point and to cite appropriate evidence. Modification to the manuscript include:

a. *Revisions to the Discussion section to comment on this point (page 18, line 606).*

5. On the whole, this paper is too long containing too many figures and descriptive/redundant results. It is highly suggested to simplify the topic and highlight the key points.

We agree that the manuscript contains a lot of data. The purpose was to triangulate methods and datasets to support the robustness of the data and increase confidence in our conclusions; however, we acknowledge that this requires explanation in the text which makes the paper long. We have reviewed the narrative and the figures and have streamlined the text to highlight key points. This has enabled; 1. inclusion of the new data and analyses requested by the reviewers and 2. ensuring the manuscript has a clearer message. We have also moved methodological explanations to the Methods section and Supplementary Notes. Together this has shortened the manuscript reducing word count by ~17%. Modification to the manuscript include:

a. *Streamlining the Results and Discussion sections to reduce redundancy and simplify the message.*
a. *Revising the Figures.*

Point-by-Point Response to Reviewer's Comment on the manuscript entitled 'Multiscale analysis and functional validation of the cellular and genetic determinants of skeletal disease' (NG-A67136R1).

Detailed responses to the reviewer's comments are highlighted in blue and proposed changes to the manuscript in *red*.

Response to Reviewer 1:

We appreciate the reviewer was impressed with the manuscript and acknowledged the efforts to address the reviewer's comments and questions. We were also pleased that the reviewer found the new endothelial osteoblast interaction studies and human single cell RNA-seq data analysis exciting. Responses to minor remaining issues are detailed below:

1. "I appreciate the effort to perform snATAC Seq for the different cell types. Here I think it is important to show the snATAC-Seq within the main figure and also take the top scoring TFs for interpretation as presentation into the results system, because this is likely the experimental evidence. Despite there is overlap it is important to note that the major TF combination differ dramatically from the SCENIC prediction (TFAP2A, EBF2, KLF15 etc.) and preference should be given to these top scoring TFs also in the description of line 248 ff".

We agree the snATAC-seq is a more direct experimental measure of chromatin accessibility and transcription factor binding sites and should be highlighted. Analysis of the snATAC-seq dataset, including the top scoring transcription factors, are now described in the Results section. Details of the snATAC-seq data have been moved into a revised figure that is now Extended Data Fig 4. This was preferred to moving to Fig 1., which would require major changes to the flow of the manuscript. These data are reported alongside the list of transcription factors that occur in both snATAC-seq and SCENIC analyses. Modifications to the manuscript include:

a. *Inclusion of the snATAC-seq data, including top scoring transcription factors, in the Results section (page 7, line 247).*
b. *A revised summary of the snATAC-seq data in Extended Data Fig 4.*

2. " I was impressed by the introduction of data spatial omics of human bone into the paper in Extended Fig. 9. It would be easier to interprete the results also be displaying the H.E. histology of the cell type distributions".

We agree with the reviewer. We have now included images of H&E-stained histological sections of each of the bone samples in Extended Data Fig. 9 which has become Extended Data Fig 10 in the revised manuscript. Modifications to the manuscript include:

a. *Inclusion of histology panels in Extended Data Fig. 10.*

Response to Reviewer 2:

We appreciate the reviewer's thoughtful comments and the request for clarification. Responses to remaining points are detailed below:

1. "Pls3 is expressed in various bone cells such as osteoblasts, endothelial cells and VSMCs. The results showed that the deletion of Pls3 led to changes in bone vasculature and some bone phenotypes (Figure 5f-i), this is consistent with the strong expression of Pls3 in VSMCs and endothelial cells (Figure 5a), but it cannot lead to a rigorous conclusion that bone vascular changes are the cause of the bone phenotype in Pls3 knockout mice. It is recommended to use conditional knockout mice or other experiments for in-depth verification".

We agree with the reviewer's important point. In the revised manuscript we were cautious in our conclusions regarding the cellular mechanism responsible for the phenotype in *Pls3* knockout mice. We

stated in line 479 "that the severe phenotype of X-linked osteoporosis **might** result from abnormalities in the bone vasculature, rather than a defect in cells of any one individual bone cell lineage". We have now reiterated this caution in the Discussion section. We also agree that analysis of mice with conditional deletion of *Pls3* would help to confirm the suggested role of VSMCs in the pathogenesis of X-linked juvenile osteoporosis. This was highlighted in the revised manuscript. We have now modified the discussion to further clarify this point and suggest this would be an appropriate approach for *Pls3*. Importantly, the appropriate mouse lines for such conditional studies are not available and would take well over a year to generate, establish and analyse. Consequently, such studies are not possible in the 8-week timeline requested for the revision. Modification to the manuscript include:

a. *Modifications to the Discussion section (page 18, line 586).*
b. *Modifications to the Discussion section (page 19, line 620).*

2. "The author described in Line 149 that a new and now largest GWAS of eBMD was used, but when studying the mouse phenotype, other parameters excluding BMD of mouse lumbar vertebrae detected by Micro-CT were given in Fig5b. Was BMD affected? In the Figure 6, the BMD data of the Ptprd-/-, Cbx6-/-, Nherf2-/-, and Agap1-/- mice were not shown either? Why did the long bone BMD of Pls3 knockout mice not change compared with that of WT mice in Figure 5c? verification".

We appreciate the reviewers comment and agree the need for clarification:

i. In all mice we measure relative bone mineral content (rBMC) by digital x-ray microradiography, a two dimensional parameter similar to areal bone mineral density (aBMD) measured by DEXA. Relative BMC benefits from the fact that skeletal samples are imaged at a higher resolution (10µm voxel resolution) and in the absence of soft tissues. This parameter is highly reproducible and has a coefficient of variation of just 2%. We have modified the Methods section to explain the similarity.
ii. In all mice microCT analysis was also performed. Trabecular bone BMD from vertebrae and femur was not included in this analysis because although analysis was performed at 5µm voxel resolution there are many trabeculae with a thickness of 20-30µm, which result in partial volume artifacts and inaccurate BMD estimation. In contrast, cortical thickness was consistently 180-220µm and scanned at 10µm voxel resolution, which mitigated the possibility of significant partial volume artifacts and provides confidence in cortical BMD measurements. For these reasons only cortical BMD measures were included. We have modified the Methods section accordingly.
iii. In Fig 5 deletion of *Pls3* resulted in decreased vertebral rBMC. Trabecular BMD was not reported due to the technical limitations described in (ii). Relative BMC was also decreased in the femurs of both male and female *Pls3* knockout mice. Cortical BMD, measured by microCT was not altered in *Pls3* knockout mice. The reduction in overall rBMC in both male and female *Pls3* mutant mice is predominantly accounted for by the significant reduction in cortical thickness and trabecular thickness rather than any major difference in BMD (Figure 5b-c, Supp Figure 7a-d). This also accounts for the reduction in bone strength observed in both male and female *Pls3* mutant mice.
iv. In Fig 6 trabecular BMD was not included for the technical reasons described in (ii).

Modification to the manuscript include:

a. *Highlighting the similarities between rBMC and aBMD in the Methods section (page 36, line 1184).*
b. *Explanation of the rationale for including cortical BMD but not trabecular BMD in the Methods section (page 37, line 1214).*

3. "Figure 6 shows Skeletal phenotypes of female Ptprd-/-, Cbx6-/-, Nherf2-/-, and Agap1-/- mice. Are there any differences in the mouse phenotypes between female and male? Were gender differences taken into account when analyzing the scRNA-seq data earlier?".

We thank the reviewer for their question. The Origins of Bone and Cartilage Disease program bone phenotyping pipeline was only performed on females as skeletal samples from the corresponding male mice were used for joint phenotyping. Therefore, bone phenotyping in male *Ptprd*$^{-/-}$, *Cbx6*$^{-/-}$, *Nherf2*$^{-/-}$, and

Agap1$^{-/-}$ mice was not undertaken and, unfortunately, the potential for sexual dimorphism cannot be explored. We specified that female mice were examined in the Results (line 407) and Methods (line 1156) sections; however, we were not explicit in saying that male were not included. We have modified the methods section to make this clear. The scRNA-seq analysis was performed on male mice as bones were generally larger, whereas the scRNA-seq analysis of adult human bone samples included both male and female samples. Analysis of these data demonstrated broad similarities between males and females. We have modified the Discussion section accordingly.
Modification to the manuscript include:

a. *Clarifying that female and not male Ptprd*$^{-/-}$*, Cbx6*$^{-/-}$*, Nherf2*$^{-/-}$*, and Agap1*$^{-/-}$ *mice underwent bone phenotyping in the Methods section (page 35, line 1157).*
b. *Modifications to the Discussion section to include reference to comparisons between sexes in future studies (page 19, line 620).*
